# Activatable self-reporting cyclization reaction for in-cell synthesis of cyclocyanines

**DOI:** 10.1039/d5sc05408g

**Published:** 2025-11-12

**Authors:** He Hang, Xia Wang, Zhaobin Wang, Chi Meng, Qihang Sun, Ziyue Yang, Fude Feng

**Affiliations:** a MOE Key Laboratory of High Performance Polymer Materials and Technology, Department of Polymer Science and Engineering, School of Chemistry and Chemical Engineering, Nanjing University Nanjing 210023 P. R. China fengfd@nju.edu.cn

## Abstract

Generally, fluorescence “turn-on” click reactions require special activation triggers and heavily rely on preformed fluorophores. Herein, we reported an activatable self-reporting click-like reaction for one-pot synthesis of cyclic cyanines (CCClick) by clicking formaldehyde (FA) into dimeric Fischer bases, using biocompatible metal ions or complexes (*e.g.*, copper, iron, and hemin) as an activator. CCClick involves FA-specific dehydration cyclization and metal-catalyzing oxidative dehydrogenation. Hemin-activated CCClick is flexible with Fischer base structures, satisfies the strict requirements for in-cell synthesis of bioimaging molecules and provides a convenient chemical tool for disease diagnosis.

## Abstract 2

A formaldehyde-participating click-like cyclization method for one-pot synthesis of cyclic cyanines was established using dimeric Fischer bases. The in-cell cyanine synthesis was activated by hemin in the mitochondria of cancer cells.

## Graphical abstract

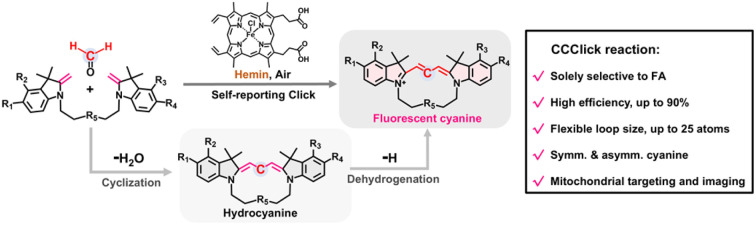

## Introduction

Click reactions can efficiently link molecules or substituents quickly at specific sites with minimal byproducts under mild conditions and have received tremendous attention in diverse fields such as chemical biology, biomedicine and theranostics.^1^ Click-type cyclization reactions have been developed as a convenient and reliable chemical tool to prepare macrocycles (*e.g.*, cyclic peptides), trigger drug release or modulate physicochemical properties of the parent molecules.^1–3^ However, to visualize targets in living cells by imaging techniques, latent fluorophores that are activatable by special mechanisms (*e.g.*, electron transfer and energy transfer) are needed.^4^ In-cell “clicking” targets to produce fluorophores can potentially boost the simplicity of molecular architecture.

**Fig. 1:**
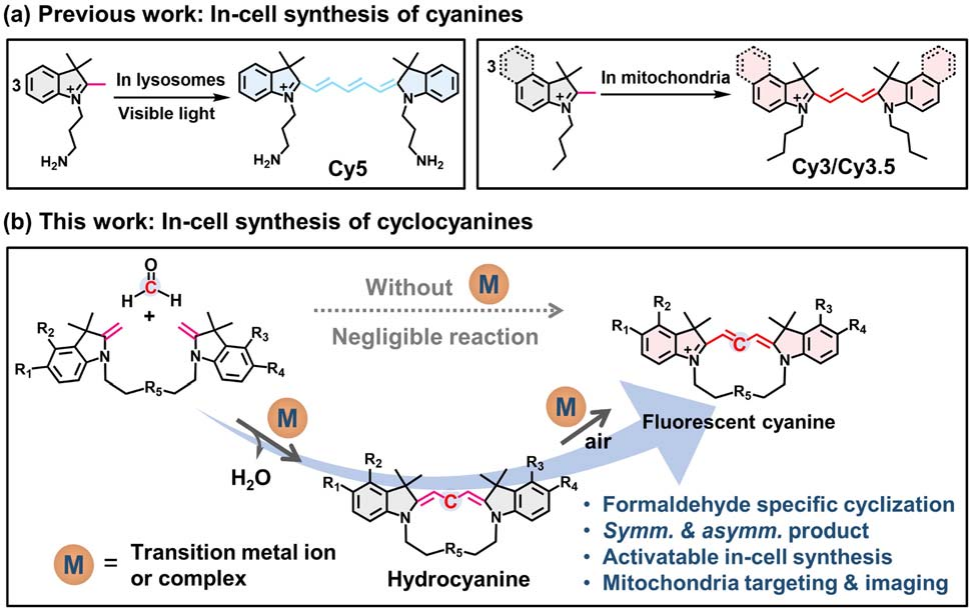
(a) Representative click-type cyclization reactions. (b) The activatable CCClick reaction of Fischer base dimers and FA involves two steps: Knoevenagel dehydration cyclization and oxidative dehydrogenation.

Polymethine cyanines are a class of bright and biofriendly fluorophores, widely used in chemo-/bio-detections, molecular labeling and imaging, disease diagnosis and therapeutics.^5^ Generally, trimethine cyanines (Cy3) are prepared from heterocycles by reactions with condensing agents under harsh conditions,^6^ which is not compatible with intracellular environments. Our previous studies revealed that indolium salts with a reactive exocyclic methyl group could be converted to pentamethine and trimethine cyanines in the specific organelles of living cells upon exposure to visible light (or reactive oxygen species (ROS) and free radicals) (Fig. 1a).^7^ The 2*H*-indolium salts and their Fischer base (FB) forms are well-documented nitrogen heterocycles for Cy3 synthesis. However, the condensation of the 2*H*-indolium salts with aldehydes *via* a classical Knoevenagel reaction often requires high temperature and alkaline conditions and proceeds at slow rates and low efficiency.^8,9^ To date, click-like chemistry for the efficient cyanine synthesis within living cells, particularly in target organelles, remains unavailable due to the difficult controllability in the complicated cellular environments.

Herein, we explored a transition metal ion (*e.g.*, copper and iron) or hemin activated click-type oxidative cyclization reaction for the synthesis of cyclic cyanines (CCClick) in open air at 37 °C (Fig. 1b). One-pot stoichiometric clicking of formaldehyde (FA) by homogenous or heterogenous Fischer base dimers includes two steps: Knoevenagel dehydration cyclization and oxidative dehydrogenation. The overall conversion is rapid, complete, clean, flexible in loop size and heterocycle structure, with water as the only byproduct, and without the need for an additional organic base. Hemin-activated CCClick demonstrates exceptional efficiency in synthesizing intramolecular [1 + 1]-type cyclic cyanines with high yield and exceptional FA-selectivity among various aldehydes. The reaction displays remarkable tolerance to high substrate concentrations up to 10 mM. Of note, the method was successfully implemented to generate fluorescent self-reporting cyclic cyanines *in situ* in living cells. Intriguingly, CCClick can be significantly activated in the mitochondria of cancerous neuronal cells.

## Results and discussion

### Synthesis of precursors and cyclic cyanines

**Fig. 2:**
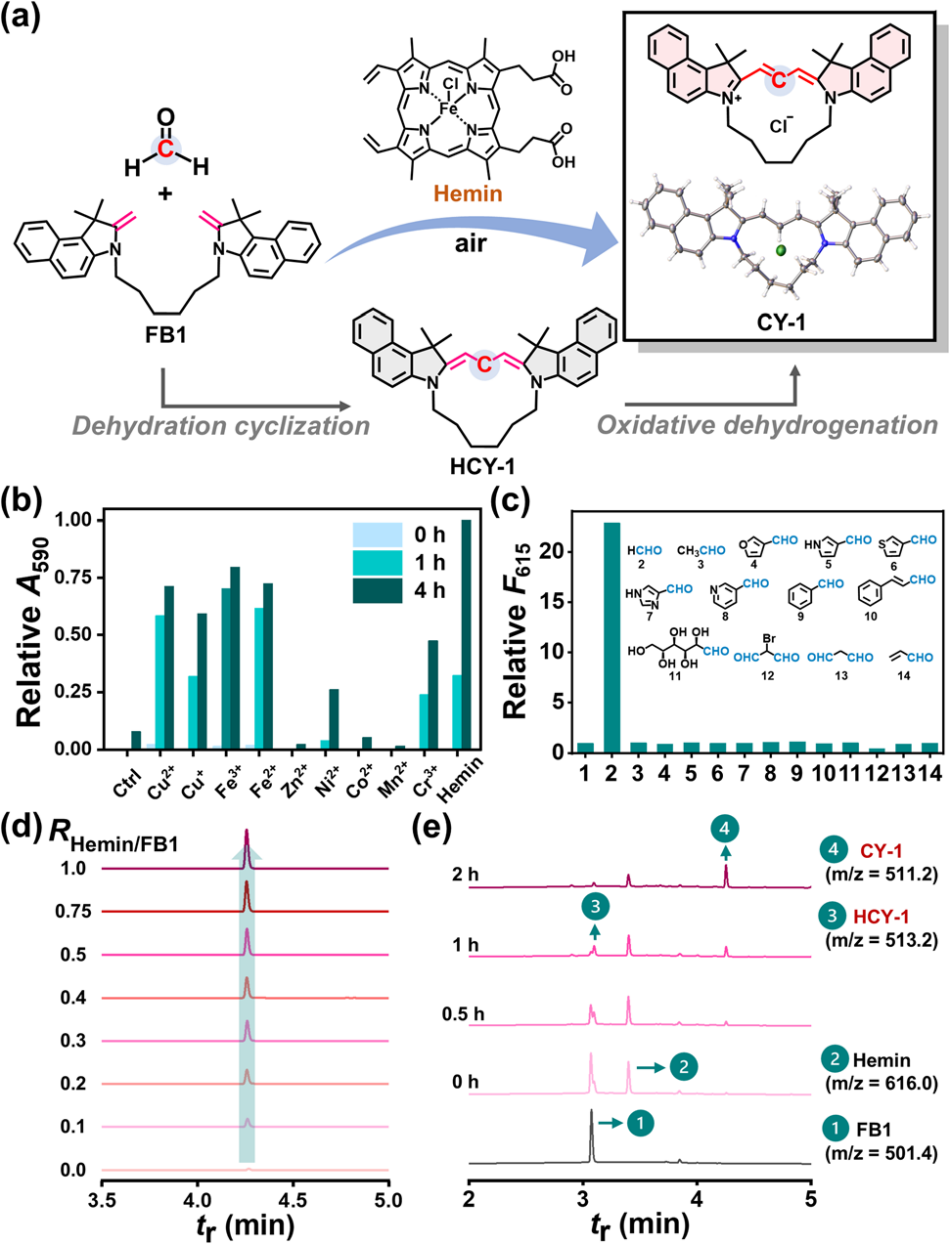
(a) Schematic presentation for FB1 to CY-1 conversion *via* hemin-activated CCClick. Inset: single crystal structure of CY-1 (CCDC deposition number: 2393670). (b) Relative *A*_590_ of solutions. The mixtures containing FB1 (1 mM), FA (10 mM) and various metal ions (0.5 mM) in DMF were incubated at 37 °C for 0–4 h and 33.3-fold diluted with methanol before UV-vis measurements. (c) Relative *F*_615_ of solutions after 333.3-fold dilution of the reaction mixtures with methanol. The reaction mixtures containing FB1 (1 mM), hemin (1 mM) and different aldehydes (10 mM) in DMF were incubated at 37 °C for 4 h. Chemical structures of various aldehydes were shown. (d) UPLC-MS analysis (590 nm). The reaction mixtures containing FB1 (1 mM), FA (10 mM) and varied concentrations of hemin (0–1 mM) were incubated at 37 °C for 0.5 h before UPLC-MS analysis. (e) UPLC-MS analysis (254 nm). The reaction mixtures containing FB1 (1 mM), FA (10 mM) and hemin (0.5 mM) were incubated at 37 °C for different times (0–2 h) before UPLC-MS analysis.

FBs bearing an exocyclic methylene group serve as the fundamental precursors in this study. A series of dimeric FBs (FB1–FB9, full synthetic route in Fig. S1) were synthesized from indolium salts, varied in the substituent on heterocycles, spacer length between heterocycles, spacer structure, and heterocycle symmetry, and characterized by NMR analysis (Fig. S2–S41). FB1 (Fig. 2a) was investigated as a model substrate in the presence of FA for a hemin-activated reaction in dimethylformamide (DMF) at 37 °C. The fluorescent product isolated was characterized by NMR and high-resolution mass spectroscopy (HRMS) (Fig. S42–S44), and identified as CY-1 by X-ray crystallographic analysis (Fig. 2a). CY-1 displayed maximum absorption and fluorescence emission at 590 nm and 615 nm in methanol, respectively (Fig. S45).

The reaction exposed to various metal ions was monitored according to the changes in absorption intensity at 590 nm (*A*_590_). The results of the metal-free experiment illustrated that air oxidation of FB1/FA to yield CY-1 was negligible (Fig. 2b). Copper and iron ions, regardless of their oxidative states, exhibited comparable activity to hemin, while superior to other metal ions.

Temperature-dependent experiments revealed that the hemin-catalyzed reaction achieved the highest yield up to 90% at 37 °C (Table S1). Notably, elevated temperature accelerated the reaction kinetics and the reaction was complete within 1 h at 70 °C, and hemin was superior to metal ions in facilitating the stoichiometric conversion of FB1 with FA to generate [1 + 1]-type cyclocyanines.

The Cy3 product can be used as a fluorescent reporter of the CCClick reaction. To assess the response selectivity of FA, we tested thirteen randomly selected aliphatic/aromatic aldehydes and compared the fluorescence intensity of the generated Cy3 product at 615 nm (*F*_615_). FA induced the fluorescence “turn on” response (Fig. 2c). In contrast, all of substituted aldehydes were unable to provoke a detectable fluorescent response. The results indicated the unique reactivity of FA in the CCClick reaction.

To check whether FB1 was unreactive to aldehydes or converted into non-fluorescent products, we tested acetaldehyde and benzaldehyde as exemplar aliphatic and aromatic aldehydes, respectively. The analysis using an ultra-performance liquid chromatograph (UPLC) equipped with mass and photo diode array (PDA) detectors clearly indicated that FB1 remained unchanged upon exposure to acetaldehyde and benzaldehyde under the same experimental conditions, which suggested the poor reactivity of FB1 to aldehydes except FA (Fig. S46). The excellent reaction selectivity to FA guarantees minimal interference from other endogenous aldehydes in the later cell studies.

The response of FB1 to FA was assessed by fluorescence spectroscopy. Kinetic analysis showed that under optimized conditions, the generation of cyanine CY-1 increased linearly with time within 2 h (Fig. S47a). A calibration curve was constructed by plotting the fluorescence intensity at 619 nm (*F*_619_) against FA concentration, yielding a linear fit with a slope of 1.21 (Fig. S47b). The standard deviation (*σ*) of *F*_619_ was determined to be 0.34 from five independent blank measurements in the absence of FA. The limit of detection (LOD) for FA was calculated to be 0.84 µM according to the formula LOD = 3*σ*/*S*, where *S* is the slope of the calibration curve.

The influence of environmental factors on the CCClick reaction with FB1 was also investigated. The reaction was unaffected by white light irradiation (20 mW cm^−2^, 4 h) (Fig. S48) and proceeded well in a broad pH range (pH 4.7–10.0) (Fig. S49) as compared to the typical FA detection method.^10^ Besides, the reaction was also well tolerant to the high concentration of glutathione (GSH, an endogenous antioxidant), with slightly reduced efficiency (by a factor of 18%) in the presence of 10 mM GSH (Fig. S50). The above results demonstrated that the CCClick reaction is compatible with environmental variables (*e.g.*, visible light, pH and GSH) and underscored its potential for applications in living systems.

The reaction activated at varied hemin concentrations was investigated by UPLC-MS. CY-1 was detected in the 590 nm channel with a retention time (*t*_r_) of 4.28 min and an *m*/*z* of 511.2 (Fig. 2d). An increase in hemin equivalents relative to FB1 (*R*_hemin/FB1_, 0–1.0) led to accelerated formation of CY-1 without the appearance of byproducts (*e.g.*, dimers or multimers) (Fig. 2d and S51). The changes in signals (254 nm channel) revealed more details of the reaction (*R*_hemin/FB1_, 0.5) (Fig. 2e): (1) FB1 consumption was nearly complete in 1 h; (2) the intermediate hydrogenated CY-1 (HCY-1, *t*_r_ 3.10 min; *m*/*z*, 513.2) (Fig. S52) was initially visible and almost disappeared after 2 h of reaction; (3) no FB1-FA adducts but HCY-1 were detected; (4) CY-1 production was blocked in the hemin-free control experiment (Fig. S53). Thus, two sequential steps were proposed in the reaction mechanism, including dehydration cyclization (*i.e.*, FA/FB1 → HCY-1) and oxidative dehydrogenation (*i.e.*, HCY-1 → CY-1), and accounted for the clean transformation.

### Reaction kinetics of hemin-activated CCClick

**Fig. 3:**
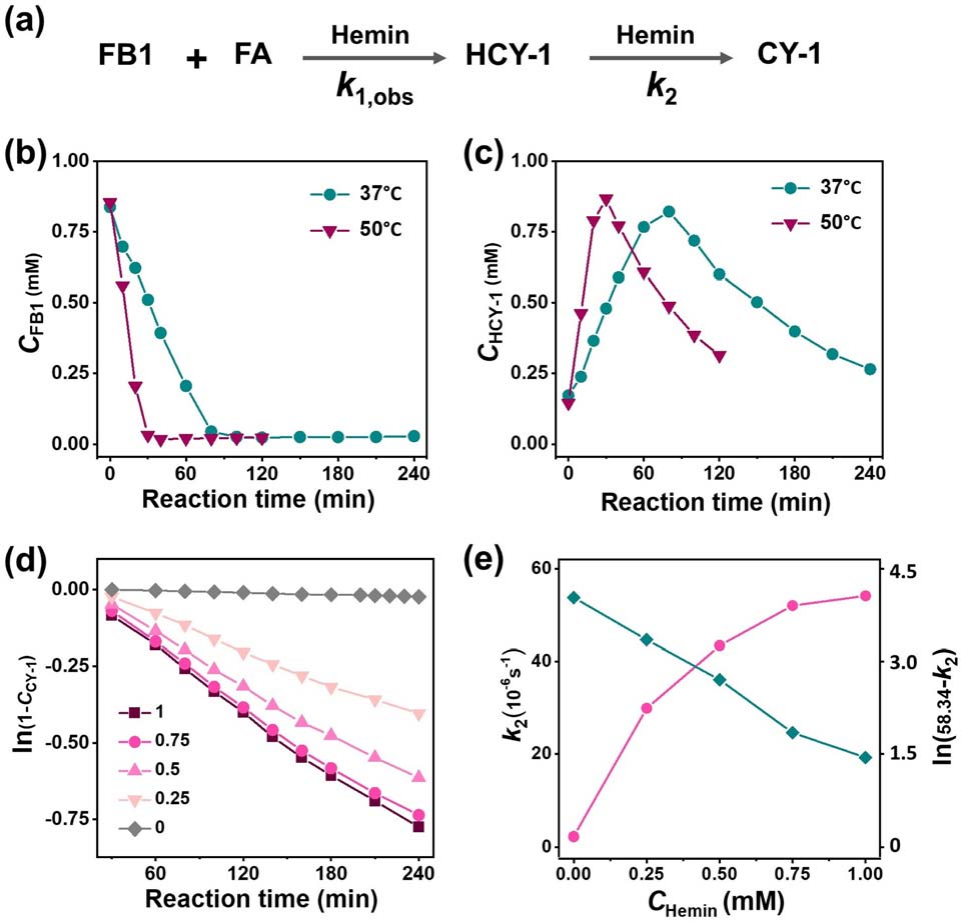
(a) Schematic diagram for two-step conversion of FB1 to CY-1. Concentration changes of (b) FB1 and (c) HCY-1 at different temperatures over a period of 0–4 h. The reaction mixture containing FB1 (1 mM), FA (10 mM) and hemin (0.5 mM) in DMF was incubated and characterized by UPLC (254 nm). (d) Plots of ln(1 − *C*_CY-1_) *versus* reaction time at various *C*_hemin_. The mixtures containing FB1 (1 mM), FA (10 mM) and different *C*_hemin_ (0–1 mM) in DMF were incubated at 37 °C and 33.3-fold diluted with methanol before UV-vis measurements. (e) Relationship between the reaction rate constant *k*_2_ and *C*_hemin_.

The rate constants for the two consecutive steps including dehydration cyclization and oxidative dehydrogenation (denoted as *k*_1_ and *k*_2_, respectively) were measured using UPLC and spectrophotometric analysis (Fig. 3a). Initially, the concentration of the reactant FB1 was monitored over time under pseudo first-order conditions with an excess of FA catalyzed by hemin at 37 °C (*R*_hemin/FB1_, 0.5) (Fig. 3b). The kinetic profile showed that the consumption of FB1 followed zero-order kinetics, suggesting that the dehydration cyclization step was independent of the reactant concentration (*k*_1,obs_, 1.7 × 10^−7^ M s^−1^). After complete FB1 depletion, the concentration of the intermediate HCY-1 decayed according to first-order kinetics (*k*_2_, 4.3 × 10^−5^ s^−1^), which corresponded to the subsequent exponential growth of CY-1 formation (Fig. 3c and d). Furthermore, by varying the reaction temperature, the kinetics data obtained under 50 °C conditions confirmed the consistency of the two-step mechanism relative to the above data acquired at 37 °C (Fig. 3b and c). The increased temperature resulted in higher observed rate constants for both steps (*k*_1,obs_, 4.7 × 10^−7^ M s^−1^; *k*_2_, 6.8 × 10^−5^ s^−1^), allowing for determination of the activation energies (*E*a_1,obs_, 67.1 kJ mol^−1^; *E*a_2_, 29.8 kJ mol^−1^).

To clarify the role of the activator (hemin), we investigated reaction kinetics of CCClick at different hemin concentrations (*C*_hemin_). The results confirmed that the rate constant *k*_2_ for the oxidative dehydrogenation step exhibited an exponential increase with *C*_hemin_ (Fig. 3e). This relationship was detailed in the following equation:*k*_2_ = 58.34 − 56.16 × exp(−*C*_hemin_/0.38)

According to the Arrhenius equation, the exponential increase in *k*_2_ suggested that hemin activates the CCClick reaction by lowering the activation energy. Taken together, the kinetic analysis revealed that hemin plays a dual role including (1) facilitating the formation of the intermediate HCY-1, which was relatively slow in the absence of hemin (*k*_1,obs_, 9.3 × 10^−8^ M s^−1^) (Fig. S54); (2) functioning as an efficient catalyst to substantially accelerate the oxidative dehydrogenation step that is inherently slow in its absence (*k*_2_, 2.2 × 10^−6^ s^−1^) (Fig. 3e).

The effect of iron forms was also evaluated in the cyclization reaction using UPLC analysis and compared with hemin. As seen in Fig. S54, in the presence of FeCl_3_, FB1 was completely consumed within 0.5 h, significantly faster than the case of hemin, implying that transition metals participate in the dehydration cyclization step. However, generation of a dimeric adduct took place with the increase in FeCl_3_ amount (*e.g.*, *R*_FeCl_3_/FB1_ > 0.5). The existence of a dimeric adduct detected at *t*_r_ 3.7 min and *m*/*z* 506.6 (Fig. S55 and 56) in the FeCl_3_-involved reaction suggested that hemin is superior to FeCl_3_ in suppressing the oligomeric reaction to obtain cyclocyanines, although at the cost of the reaction rate. Of note, the CCClick mediated by hemin proceeded well even using significant excess of FA in high concentrations (10 mM) (Fig. 2d), without formation of oligomeric products. Taken together, hemin is advantageous in controllability of cyclization reactions which is key to afford cyclocyanines, without strict requirements like high dilutions of reactants and a limited stoichiometry range of reaction components.

### Mechanism of hemin-activated CCClick

**Fig. 4:**
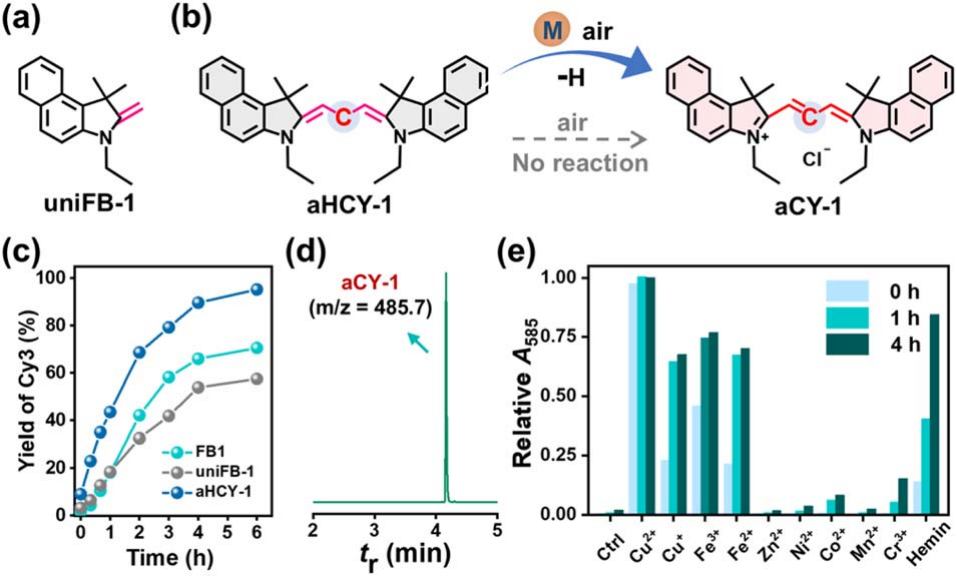
(a) Chemical structure of uniFB-1. (b) Schematic presentation for aHCY-1 to aCY-1 conversion. (c) Time-dependent conversion efficiency of hemin-activated reactions. (d) UPLC-MS analysis (585 nm) of a reaction mixture containing aHCY-1 (1 mM) and hemin (1 mM) at 37 °C for 6 h. (e) Relative *A*_585_ of reaction mixtures after dilution with methanol. Reaction mixtures contained aHCY-1 (1 mM) and various metal ions (0.5 mM) and were incubated in DMF at 37 °C.

Based on Fig. 2e and S53, we inferred that the exceptional selectivity of FA over other aldehydes was associated with the dehydration cyclization step. The effect of metal catalysts on the key intermediate (*i.e.*, hydrogenated Cy3) is also crucial for the understanding of overall transformation. aHCY-1 (Fig. S57–S58), an acyclic analogue of HCY-1, was prepared from uniFB-1 (Fig. 4a and b) and investigated instead of HCY-1 which was unavailable with high purity. Activated by hemin, aHCY-1 was gradually converted to aCY-1 (Fig. S59–S61) with efficiency up to 96% within 6 h without detectable byproducts, faster than the use of FB1 or uniFB-1 (Fig. 4c, d and S62). The oxidation process could be boosted by copper and iron ions as well as hemin (Fig. 4e), and surprisingly, could be instantly complete upon exposure to Cu^2+^ with near-unity efficiency, illustrating extreme reactivity of hydrogenated cyanines to Cu^2+^. As the control, aHCY-1 alone was unreacted in the absence of a metal catalyst.

Oxygen (O_2_) could affect the metal-involved oxidation process to some extent but was not an indispensable factor. In oxygenated DMF, the oxidation of aHCY-1 was accelerated, while under deoxygenated conditions the aCY-1 production remained at a moderate level (Fig. S63). Meanwhile, the noticeable outperformance of FB1 over uniFB-1 in cyanine generation underscores the critical effect of cyclization. Hence, we inferred that the metal catalyst plays important roles in both Knoevenagel dehydration cyclization and oxidative dehydrogenation steps. The effect of bio-friendly (and endogenously available) metal catalysts avoids the use of strong oxidants like 2,3-dichloro-5,6-dicyano-1,4-benzoquinone (DDQ) reported in other studies.^11^ Based on the above experimental evidence, we proposed a plausible reaction mechanism for the hemin-activated CCClick in Fig. 2a.

### Structure extension and spectral properties of cyclic cyanines

The CCClick reaction was investigated across diverse Fischer bases. Heterodimers FB2 and FB3 composed of indole and benzoindole moieties were selected as model precursors for generating asymmetrical cyclic cyanines. Setting the molar ratio at 1 : 5 : 1 (Fischer base/FA/hemin), cyclic cyanines (CY-1–CY-9) were produced with 24–90% efficiency (Table 1 and Fig. S64) and easily isolated for NMR & HRMS analysis (Fig. S42–S44 and S65–S88). The relatively high yield of CY-4 in comparison to CY-5 (85% *versus* 31%) may be ascribed to the effect of electron donating properties of the methyoxyl group. Even with large ring sizes, CY-6, CY-7 and CY-9 were isolated with high purity, benefiting from the clean transformation. The CCClick reaction is a method promising for synthesis of macrocylic cyanines. The analytical data conclusively confirmed that all cyclocyanines adopt a trans-configuration on the trimethine bridge.

**Table 1:** Products of the hemin-catalyzed CCClick reaction^a^^,^^b^

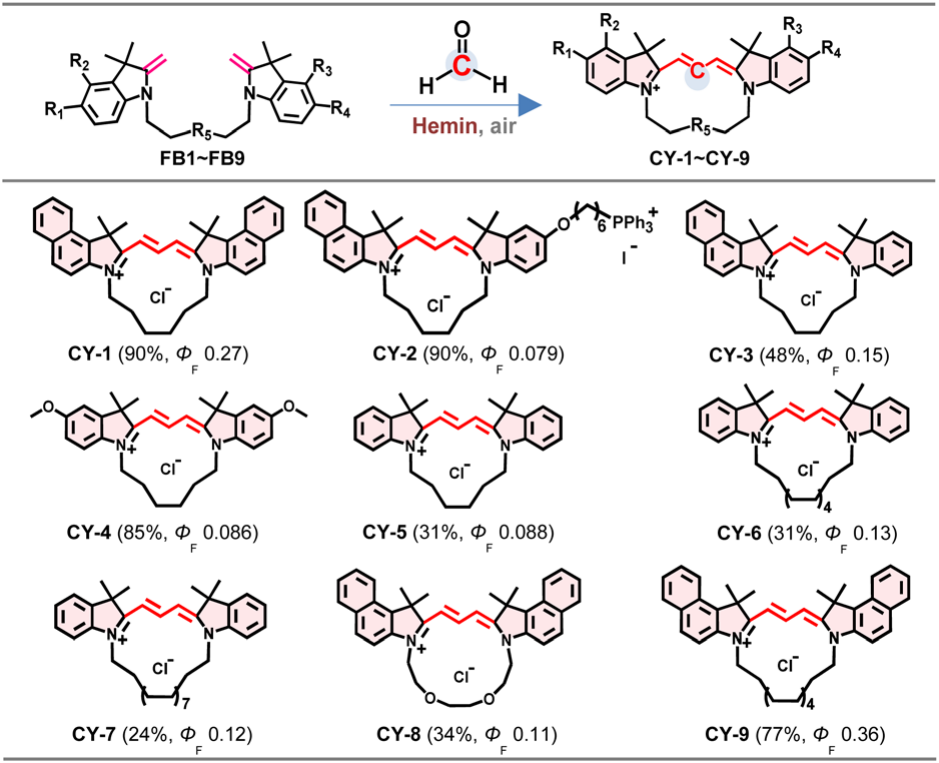

a Reaction conditions: FB1-FB9 (1 mM), FA (10 mM) and hemin (1 mM) in DMF at 37 °C. Conversion efficiency and *Φ*_F_ were shown in brackets.

b Fluorescence quantum yields of CY-1–CY-4 and CY-8–CY-9 were measured in DCM using Cresyl Violet (*Φ*_F_ = 0.54 in methanol at 25 °C) as the reference; fluorescence quantum yields of CY-5–CY-7 were measured in DCM using rhodamine B (*Φ*_F_ = 0.66 in ethanol at 25 °C) as the reference. 12.

The ultra-violet (UV-vis) absorption and fluorescence spectra of cyclocyanines are shown in Fig. S45 and S89–S96, and photophysical properties (*e.g.*, maximum absorption and emission wavelengths, molar extinction coefficients, Stokes shifts, and fluorescence quantum yields) are summarized in Table S2. For clarity, the fluorescence quantum yields (*Φ*_F_s) are also shown in Table 1 in the range of 0.079–0.36, which were comparable to those of typical acyclic Cy3 dyes frequently utilized in bioimaging studies.^9^ Specifically, the cyclic cyanines CY-1 and CY-5 showed *Φ*_F_s comparable to those of their ethyl-substituted acyclic analogues (aCY-1 and aCY-5, with *Φ*_F_s of 0.076 and 0.30, respectively).

Furthermore, the cyclization strategy enables the tuning of fluorescence performance. The increase in ring size is favourable for enhancement of *Φ*_F_. For example, the larger ring derivative CY-9 exhibited a 33% higher *Φ*_F_ than CY-1. The *Φ*_F_s of CY-6 and CY-7 were enhanced by 47% and 36%, respectively, relative to that of CY-5. This kind of *Φ*_F_ enhancement is likely due to the reduced intermolecular π–π stacking and restricted non-radiative decay pathways conferred by the larger macrocyclic structure.

### Hemin-activated in-cell synthesis of cyclocyanines

**Fig. 5:**
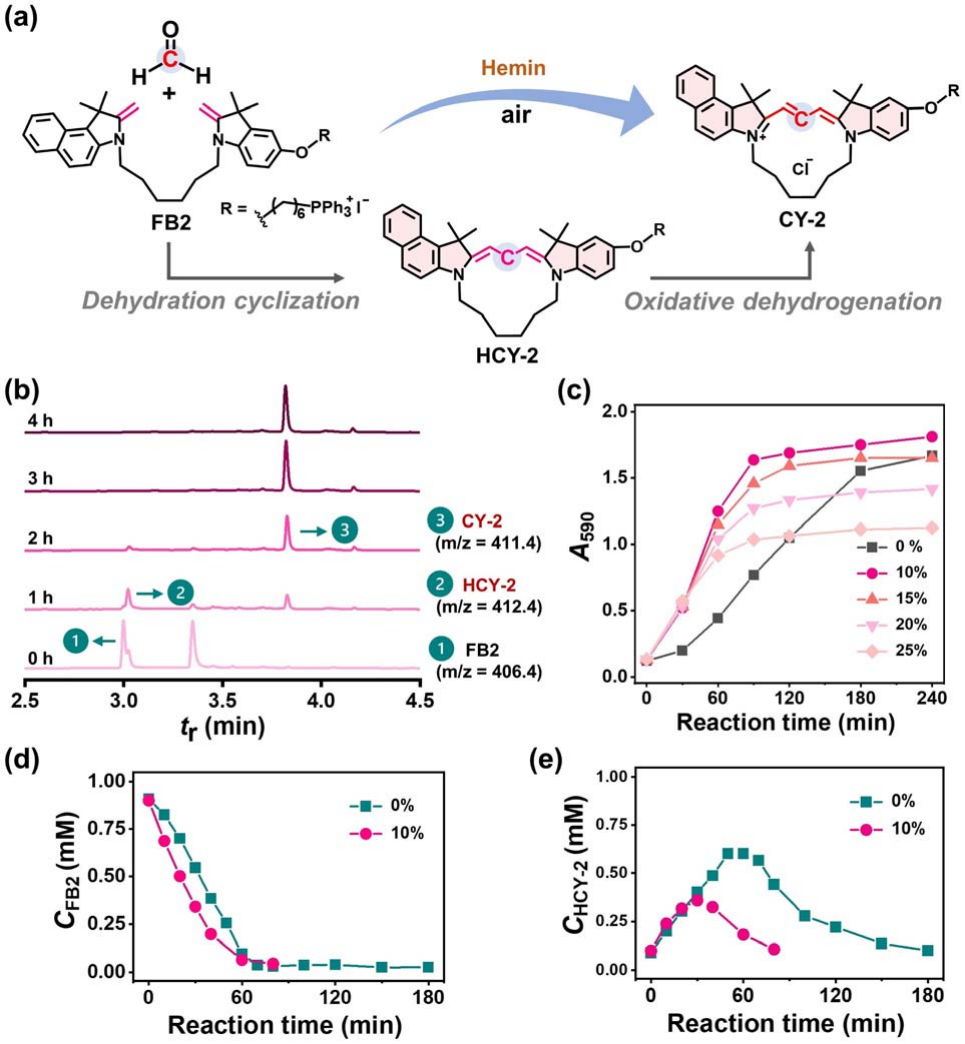
(a) Schematic presentation for FB2 to CY-1 conversion *via* hemin-activated CCClick. (b) UPLC-MS analysis of the reaction mixture recorded in a 254 nm channel (PDA detector). Reaction: FB2 (1 mM) and FA (10 mM) with hemin (1 mM) in DMF at 37 °C for 0–4 h. (c) Plot of *A*_590_ as a function of reaction time. The reaction of the mixture containing FB2 (1 mM), FA (10 mM) and hemin (1 mM) in 0–25% (v/v) water/DMF solvent was performed at 37 °C. The mixture was diluted 33.3-fold with methanol before spectrophotometric analysis. Plot of *C*_FB2_ (d) or *C*_HCY-2_ (e) as a function of reaction time. The mixture containing FB2 (1 mM), FA (10 mM) and hemin (1 mM) was incubated in 0% or 10% (v/v) water/DMF solvent at 37 °C and analysed by UPLC (254 nm).

Installed with a cationic triphenylphosphorous (TPP) group as a mitochondria-targeting ligand (Fig. 5a), FB2 is a satisfying asymmetrical substrate for hemin-activated CCClick for potential use in cell studies. The UPLC-MS data (in the PDA 254 nm channel) revealed that the transformation was very clean and obeyed the two-step mechanism, with hydrogenated CY-2 (HCY-2) as the key intermediate (Fig. 5b and S97a) detected at *t*_r_ 3.02 min with an *m*/*z* of 412.4. In the 590 nm channel (Fig. S97b and S98), only CY-2 was detected at *t*_r_ 3.83 min with an *m*/*z* of 411.4 without signs of any byproduct, demonstrating that the cyclization process is prevailing over dimeric/oligomeric reactions and laying a solid foundation for the *in situ* synthesis of structurally well-defined cyclocyanines in living cells.

To understand the effect of the aqueous intracellular environment, we carried out the hemin-activated CCClick reaction in the water/DMF mixed solvent at 37 °C. Kinetic analysis revealed that the CCClick reaction of FB2 proceeded efficiently across 0–25% (v/v) water fractions (Fig. 5c). The dehydration cyclization step of FB2 followed zero-order kinetics, with observed rate constants (*k*_1,obs_) of 2.3 × 10^−7^ M s^−1^ and 2.9 × 10^−7^ M s^−1^ in 0% and 10% (v/v) water/DMF solvent, respectively. Correspondingly, the first-order kinetics associated with the oxidative dehydrogenation process afforded rate constants (*k*_2_) of 2.6 × 10^−4^ s^−1^ and 4.6 × 10^−4^ s^−1^ in the same solvent systems. Notably, the addition of water accelerated the overall CCClick reaction kinetics, resulting in a significantly higher yield of CY-2 within 1 h as compared to the pure DMF system. This kinetic acceleration may suggest that the CCClick reaction is favourable in the physiological environment of live cells.

A mitochondrion was selected as the target organelle for rapid *in situ* cyanine synthesis in view of the following considerations: (1) the very negative mitochondrial membrane potential (MMP, approx. −180 mV) in living cells favors accumulation of large amounts of TPP-tethered precursors in the mitochondrial inner membrane (MIM);^13,14^ (2) hemin is accumulated in MIM *via* the 2-oxoglutarate carrier (OGC, mitochondrial transporter of 2-oxoglutarate);^15^ (3) mitochondrial functions are highly abnormal in cancer cells.^16^ MIM is thus an ideal site where hemin activates the reaction of Fischer base with membrane permeable FA.

**Fig. 6:**
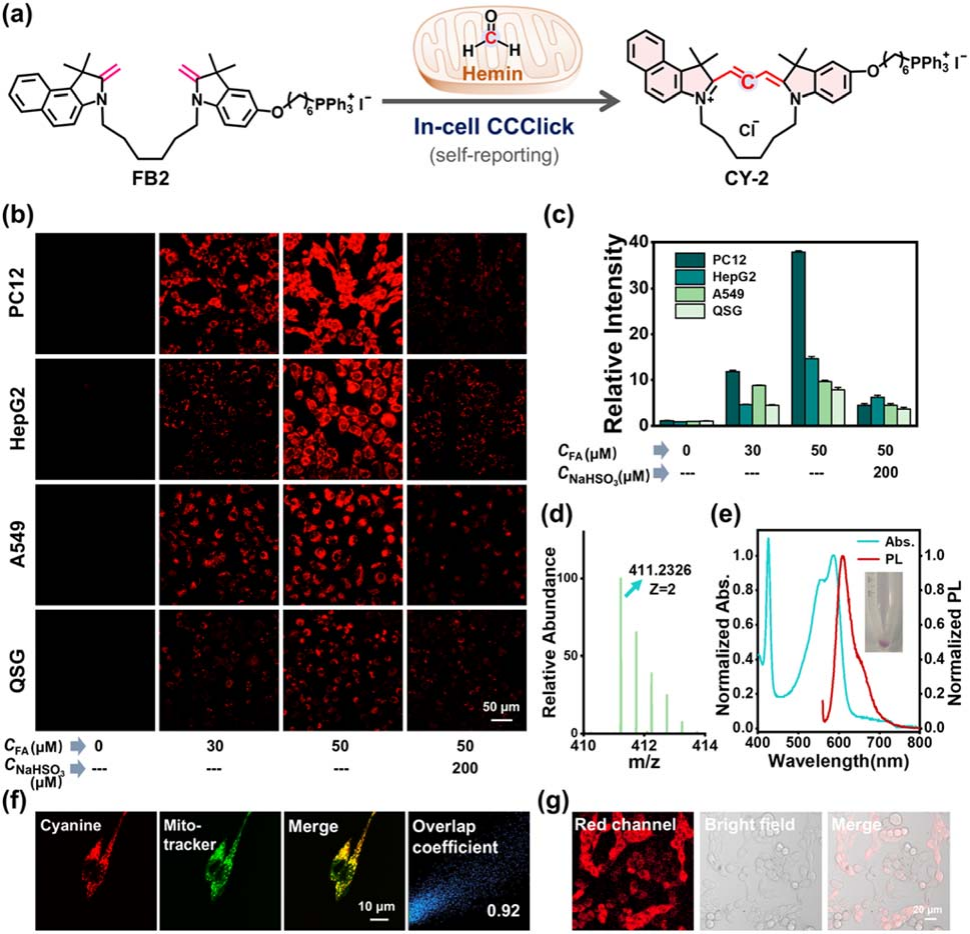
(a) Schematic presentation of hemin-activated CCClick to generate CY-2 in mitochondria. (b) Fluorescence images of living cells after various treatments. Hemin, 0 or 5 µM; FB2, 5 µM. (c) Mean relative fluorescence intensities derived from (b). Error bar represents the standard deviation of relative fluorescence intensity measurements in independent cells (mean ± SD), *n* = 3. (d) HRMS analysis and (e) normalized absorption/fluorescence spectra of PC12 cell lysate after hemin-activated CCClick. (f) CLSM images of PC12 cells after hemin-activated CCClick and Mitotracker staining. (g) CLSM images of co-cultured PC12/QSG cells after hemin-activated CCClick.

Concerning the pathological effects of FA and hemin on malignancies and neurological diseases,^17^ neuronal cancer cell line PC12 is chosen for *in vitro* study and compared to other cancer cells (HepG2 and A549). Hemin, FA, and Fischer bases (*e.g.*, FB1 and FB2) showed low cytotoxicity (Fig. S99 and 100). In-cell synthesis of CY-2 was studied by fluorescence imaging of FB2-treated PC12 cells with CY-2 as the fluorescent self-reporter (Fig. 6a). In FA-free cells, no emission signals were detected (Fig. S101). The very low signal from PC12 cells treated with FB2 and FA was attributable to the biocatalysis of endogenous redox species. By contrast, bright fluorescence was induced after 0.5 h-incubation of hemin (5 µM)/FB2 (5 µM) pre-treated cells with FA (30 µM and 50 µM). The fluorescence enhancement was almost abolished if the cells were pre-treated with NaHSO_3_ that was utilized as a strong FA scavenger (Fig. 6b and c), which verified that FA participated in cyanine synthesis in PC12 cells.

The self-reporting fluorescence was also observed in HepG2 and A549 cells under the same conditions (Fig. 6b), while with lower intensity than in PC12 cells (Fig. 6c). Particularly, normal liver QSG cells displayed very weak fluorescence after treatment with the same protocol (Fig. 6b and c), reflecting that the CCClick reaction was inefficient in normal cells as compared to cancer cells.

To verify the structure of the fluorescent product in the living cells, we analyzed cell lysate after hemin/FB2/FA treatment on PC12 cells. CY-2 was detected from the red lysate by HRMS analysis (found *m*/*z* 411.2326 *versus* calc. *m*/*z* 411.2334) (Fig. 6d). The UV-vis absorption and fluorescence spectra of cell lysate were also in good agreement with those of CY-2 (Fig. 6e). Therefore, the CCClick reaction was successful in forming fluorescent cyclocyanines in PC12 cells and well tolerated the cellular environment.

To clarify the location of CY-2, we carried out cell imaging experiments using confocal laser scanning microscopy (CLSM) after PC12 cells were treated under CCClick conditions. The imaging data confirmed excellent mitochondrial localization of *in situ* generated CY-2, with a high Pearson's coefficient (0.92) (Fig. 6f). Tethering a TPP moiety that enhanced mitochondrial accumulation, FB2 was much superior to FB1 in in-cell CY-2 synthesis (Fig. S102).

To further evaluate the potential of CCClick in discriminating cancer cells from normal cells in the identical environment, we tested *in situ* CY-2 synthesis on a co-culture model by co-culturing PC12 and QSG cells that differed in shapes and sizes.

After treatment with FB2 (2 µM) and hemin (5 µM), PC12 cells were responsive to the exposure of FA (50 µM), with fluorescence intensity 17.9-fold that of co-cultured QSG cells (Fig. 6g and S103). In another experiment, FB2 outperformed FB1 in cyanine synthesis in PC12 cells (Fig. S104), thanks to the TPP ligand-enhanced accumulation in cancer cells.^14,18^ The above results suggested that the CCClick method using FB2 is promising for cancer cell-selective bioimaging.

**Fig. 7:**
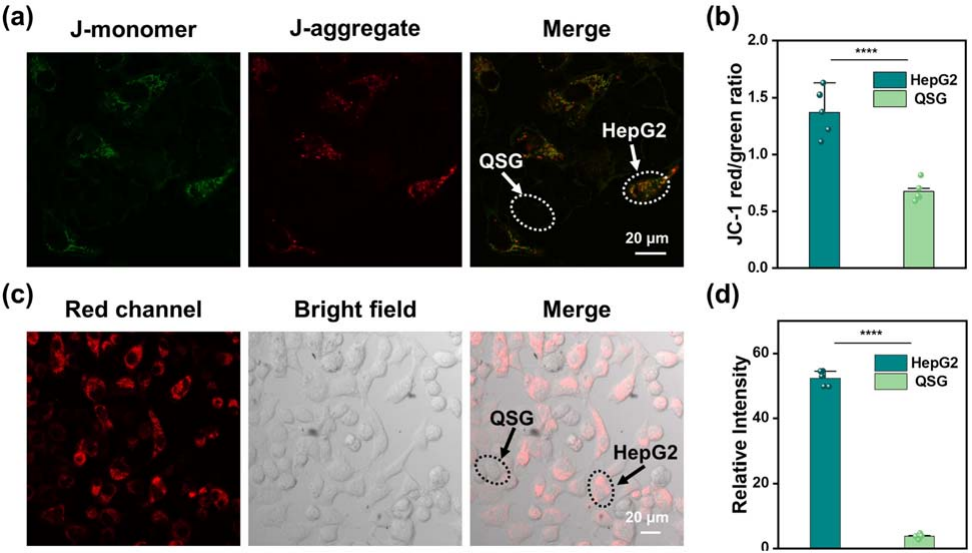
(a) CLSM images of co-cultured HepG2/QSG cells stained with JC-1. The cells within randomly selected elliptical regions were distinguished based on their morphological characteristics. (b) The ratio of red and green fluorescence intensity in co-cultured HepG2/QSG cells stained with JC-1. The error bar represents the standard deviation of fluorescence intensity measurements in independent cells (mean ± SD), *n* = 5, **** *p* < 0.0001. (c) CLSM images of co-cultured HepG2/QSG cells after hemin-activated CCClick. The cells within randomly selected elliptical regions were distinguished based on their morphological characteristics. (d) Mean relative fluorescence intensities of co-cultured HepG2/QSG cells. The error bar represents the standard deviation of relative fluorescence intensity measurements in independent cells (mean ± SD), *n* = 5, **** *p* < 0.0001.

The observed cellular selectivity arises from the more negative MMP in cancer cells (*e.g.*, PC12 and HepG2) as compared to normal cells (*e.g.*, QSG).^19^ The imaging data of JC-1 assay using the commercial MMP-sensitive JC-1 probe^20^ to stain co-cultured HepG2 and QSG cells indicated that HepG2 was stained with higher brightness than QSG cells in green and red channels (Fig. 7a, b and S105) with a greater red-to-green fluorescence ratio, which supported the superior accumulation of cationic cyanine dyes in cancer cells. In another co-culture model by co-culturing HepG2 and QSG cells, the treatment with FB2, FA and hemin led to bright fluorescence in the HepG2 cells relative to QSG cells, suggesting that CCClick is more efficient in HepG2 cells in the same culturing environment due to the difference in MMP (Fig. 7c and d).

## Conclusions

In summary, we developed an activatable cyclization method for one-pot synthesis of cyclic cyanines by clicking FA into dimeric Fischer bases. The CCClick reaction is clean and proceeds in two steps: FA-specific dehydration cyclization (Knoevenagel condensation) and metal-dependent oxidative dehydrogenation. The advantages of the CCClick reaction are multi-fold: (1) the reaction runs smoothly in open air at 37 °C without external requirements (*e.g.*, acid/base, strong oxidants, and high temperature); (2) cyclization is efficient, loop-flexible and exclusively selective to FA; (3) the reaction is suitable to obtain both symmetrical and asymmetrical cyclocyanines and avoids acyclic byproducts (*e.g.*, multimers); (4) the reaction is nicely compatible with the cellular environment and can be activated by hemin to rapidly generate self-reporting cyclocyanines in the living cells. In addition, intracellular CCClick in mitochondria exhibits potential applications for cancer cell-selective diagnosis.

## Author contributions

F. Feng developed the concept and designed the experiments. H. Hang performed the experimental studies and analyzed the data. H. Hang and F. Feng wrote the main manuscript. All authors discussed the results and assisted during manuscript preparation.

## Conflicts of interest

There are no conflicts to declare.

## Supplementary Material

SC-017-D5SC05408G-s001

SC-017-D5SC05408G-s002

## Data Availability

CCDC 2393670 contains the supplementary crystallographic data for this paper.^21^ The data supporting this article have been included as part of the supplementary information (SI). Supplementary information: materials and instruments, experimental details (*e.g.*, reaction kinetics analysis, cell studies, and all synthesis routes), experimental data (*e.g.*, NMR, HRMS, UPLC-MS, and spectroscopic characterization), and X-Ray crystal structure analysis. See DOI: https://doi.org/10.1039/d5sc05408g.
