# Supplementary material for: Activatable self-reporting cyclization reaction for in-cell synthesis of cyclocyanines
Source: Chem Sci. 2025 Nov 12;17(3):1785–91. doi: 10.1039/d5sc05408g (PMC12666538; doi:10.1039/d5sc05408g)
Supplement: SC-017-D5SC05408G-s001 [file SC-017-D5SC05408G-s001.pdf]

Supplementary Information (SI) for Chemical Science.
This journal is © The Royal Society of Chemistry 2025

Supplementary Information (ESI)

Activatable Self-Reporting Cyclization Reaction for In-Cell Synthesis of Cyclocyanines

He Hang, Xia Wang, Zhaobin Wang, Chi Meng, Qihang Sun, Ziyue Yang and Fude Feng

MOE Key Laboratory of High Performance Polymer Materials and Technology, Department of Polymer Science and Engineering

School of Chemistry and Chemical Engineering, Nanjing University

Nanjing 210023 (P. R. China)

E-mail: fengfd@nju.edu.cn

Table of Contents

Table of Contents 2
1. General methods 3
1.1 Materials and Instruments 3
1.2 Single crystal X-ray diffraction 3
1.3 Reaction kinetics experiment and analysis 3
1.4 Detection of Fluorescence quantum yield 4
1.5 The limit of detection (LOD) for FA 4
1.6 Cell culture 4
1.7 In-cell synthesis of Cy3 4
1.8 Assay of mitochondria colocalization 4
1.9 CLSM imaging of co-cultured cells 4
1.10 JC-1 assay 5
1.11 HRMS analysis of cell lysate 5
1.12 Cell viabilities assay 5
2. Substrate synthesis and characterization 5
2.1 Preparation of Indo1−Indo9 5
2.2 Preparation of FB1−FB9 9
2.3 Preparation of Cy3 12
3. Supplementary table and figures 16
4. X-Ray Crystal Structure Analysis 63
5. References 66

1. General methods

1.1 Materials and Instruments

All reagents and solvents from commercial suppliers were used without any purification unless otherwise stated.

All NMR spectra were recorded on a Bruker AMX 400 spectrophotometer, using TMS as an internal standard. UV-Vis absorption spectra were measured on a UV-2600 spectrophotometer (Shimadzu, Japan). Fluorescence spectra were measured on a F-7000 fluorimeter (Hitachi, Japan). Low-resolution mass spectra were obtained using a micromass GC-TOF mass spectrometer with Electrospray Ionization (ESI). High-resolution mass spectra (HRMS) were taken on a Q Exactive high resolution mass spectrometer (Thermo Fisher, Germany) and reported as positive m/z. Fluorescence images were taken on an Eclipse Ti-s inverted fluorescence microscope (Nikon, Japan) and LSM 710 confocal laser scanning microscope (Zeiss, Germany). Single-crystal X ray diffraction intensity data were collected on a Bruker D8 Venture diffractometer equipped with liquid metal target (Bruker AXS, Germany).

Waters ACQUITY H-Class UPLC system and a SQD2 single quadrupole mass spectrometer equipped with ESI source were used to monitor the reaction process. Analytical LC was performed with Waters ACQUITY PDA detectors using a Water ACQUITY UPLC BEH C18 column (1.7 μm, 3×100 mm) eluting with a gradient of 30%-95% acetonitrile for 5 min in 0.2% acetic acid at 40°C. After LC separation, the mass to charge ratio (m/z) of the ionized analyte was collected.

1.2 Single crystal X-ray diffraction

Preparation of single crystal: Single crystals of **CY-1** were prepared by liquid phase diffusion method. **CY-1** (8 mg) was dissolved in dichloromethane (DCM, 0.5 mL), filtered, added to an NMR tube, and carefully dripped n-pentane (1.8 mL) along the tube wall. After 8 days of standing at room temperature, a purple single crystal was formed at the liquid phase interface between DCM and n-pentane.

The crystal was kept at 193.00 K during data collection using X-ray single crystal diffractometer. Using Olex2,[1] the structure was solved with the olex2.solve[2] structure solution program using Charge Flipping and refined with the SHELXL[3] refinement package using CGLS minimisation.

1.3 Reaction kinetics experiment and analysis

The reaction kinetics were investigated by recording reactant and product concentrations via UPLC and UV-Vis spectroscopy. Reaction was initiated by combining **FB1/FB2** (1 mM), FA (10 mM), and hemin (0–1 mM) in DMF at constant temperature (e.g., 37°C and 50°C). For UPLC analysis, samples were withdrawn at intervals, diluted with acetonitrile/water (*V*/*V*, 1: 1), and analyzed to quantify **FB1/FB2** and the intermediates **HCY-1/HCY-2**. For UV-Vis analysis, samples were similarly withdrawn and diluted with methanol, and the absorption intensity at 590 nm (A_{590}) was measured to monitor the formation of **CY-1/CY-2**. It should be noted that after the complete consumption of **FB1/FB2**, the increase in **CY-1/CY-2** corresponded to the decay of **HCY-1/HCY-2**.

By constructing concentration-time profiles, key kinetic parameters (e.g., rate constants *k*, reaction orders *n* and activation energy *E*a) were determined under pseudo first-order conditions with an excess of FA catalyzed by hemin. Finally, kinetic analysis revealed that the dehydrative cyclization step was zero-order, while the oxidative dehydrogenation step was first-order. Their respective rates are quantitatively described by the following equations:

$$r_1 = k_{1,\mathrm{obs}} \qquad Eq.1$$

$$r_2 = k_2 * c_{\mathrm{HCY\text{-}1}} \qquad Eq.2$$

$$k_2 = 58.34 - 56.16 * \exp(-C_{\mathrm{Hemin}}/0.38) \qquad Eq.3$$

where $\boldsymbol{r_1}$ and $\boldsymbol{r_2}$ denote the reaction rates for the dehydrative cyclization and oxidative dehydrogenation steps, respectively; $\boldsymbol{k_{1,\mathrm{obs}}}$ and $\boldsymbol{k_2}$ are the corresponding rate constants, and $\boldsymbol{C_{\mathrm{Hemin}}}$ represents the concentration of hemin.

The rate constant $\boldsymbol{k_2}$ was obtained from kinetic fitting of the **HCY-1** decay or **CY-1** formation curves. Furthermore, the relationship between $\boldsymbol{k_2}$ and $\boldsymbol{C_{Hemin}}$ (***Eq.*** **3**) was well-described by an exponential function, affording a high correlation (R^2 = 0.998).

1.4 Detection of Fluorescence quantum yield

Relative fluorescence quantum yield (***Φ***) of cyclic cyanines were measured on Hitachi F-7000 fluorimeter using Cresyl Violet (***Φ*** = 0.54 in methanol at 25°C) or Rhodamine B (***Φ*** = 0.66 in ethanol at 25°C)[4] as the reference, and calculated according to the following equation:

$$\Phi_{\mathrm{s}} = \Phi_{\mathrm{r}} \frac{F_{\mathrm{s}}}{F_{\mathrm{r}}} \frac{A_{\mathrm{r}}}{A_{\mathrm{s}}} \frac{n_{\mathrm{s}}^2}{n_{\mathrm{r}}^2} \qquad Eq.4$$

where ***F*** represents the integrated intensity of the fluorescence spectrum; ***A*** refers to the absorbance of the solution at the excitation wavelength, ***n*** represents the refractive index of the solvent, **s** and **r** mean the test sample and reference, respectively.

1.5 The limit of detection (LOD) for FA

The response of **FB1** to FA was assessed by fluorescence spectroscopy. Stock solutions of **FB1** (3.0 mM), $FeCl_3$ (3.0 mM), and FA (3.0 mM) were first prepared in DMF. **FB1** (10 μ L) and $FeCl_3$ (5 μ L) were transferred to a test tube containing DMF. Different volumes of the FA stock solution were then added to achieve the desired concentrations, and the mixture was diluted with DMF (3 mL). After incubation at 37°C for 2 h, fluorescence emission spectra were recorded upon excitation at 590 nm (λ_{em} = 600−800 nm).

A calibration curve was constructed by plotting the fluorescence intensity at 619 nm (F_{619}) against the corresponding FA concentration. The limit of detection (LOD) for FA was calculated using the following equation:

$$\mathrm{LOD} = 3\sigma/S \qquad Eq.5$$

where **S** is the slope of the calibration curve; **σ** is the standard deviation of F_{617} determined from five independent blank measurements in the absence of FA.

1.6 Cell culture

Cells (PC12/HepG2/A549/QSG cells) were cultured in DMEM medium containing 10% fetal bovine serum at 37°C in an atmosphere containing 5% CO_2 for 24 h. The cell lines PC12, A549 and HepG2 were obtained from Jiangsu KeyGEN BioTECH Corp., Ltd. The cell lines QSG was purchased from Wuhan Pricella Biotechnology Co., Ltd.

1.7 In-cell synthesis of Cy3

Cells (PC12 or QSG cells) were cultured in DMEM medium containing 10% FBS at 37°C in an atmosphere containing 5% CO_2, incubated with hemin (5 μM) for 4.5 h in culture medium and washed three times with PBS. Then, the cells were incubated with FB1 or FB2 (5 μM) for 2 h, washed with PBS and incubated with FA (0−50 μM) for 0.5 h, and subjected to fluorescence imaging.

1.8 Assay of mitochondria colocalization

PC12 cells were cultured in DMEM medium containing 10% FBS at 37°C in an atmosphere containing 5% CO_2 for 24 h, incubated with hemin (5 μM) for 4.5 h and washed with PBS. The cells were incubated with **FB2** (0.1 μM, 1 h), washed with PBS and incubated with FA (50 μM, 0.5 h). After washing, Mitochondria green tracker (0.1 μM) was added to the cells for 1 h-staining before CLSM imaging. Cy3: excited at 543 nm, emission collected at 600−700 nm. Mitochondria green tracker: excited at 488 nm, emission collected at 500−600 nm.

1.9 CLSM imaging of co-cultured cells

PC12/HepG2 and QSG cells were co-cultured in DMEM medium containing 10% FBS at 37°C in an atmosphere containing 5% CO_2, incubated with hemin (5 μM) for 4.5 h and washed with PBS. The cells were treated with **FB1** or **FB2** (2 μM) for 2 h, washed with PBS and incubated with FA (50 μM) for 0.5 h before CLSM imaging. Cy3: excited at 543 nm, emission collected at 600−700 nm.

1.10 JC-1 assay

The mitochondrial membrane potential was monitored with the fluorescent probe JC-1. HepG2 and QSG cells were seeded in a 40 mm 4-well glass-bottomed plate at a density of 1 × 10^5 cells per well for 24 h. The cells were then incubated with 500 μL of JC-1 working solution at 37 °C for 20 min and washed twice with 1× incubation buffer. CLSM imaging was performed thereafter using the following settings: Green fluorescence channel: λex = 488 nm, λem = 500−540 nm; Red fluorescence channel: λex = 543 nm, λem = 560−620 nm.

1.11 HRMS analysis of cell lysate

PC12 cells were cultured in DMEM medium supplemented with 10% FBS at 37°C in an atmosphere containing 5% CO_2 and treated with hemin (10 μM, 4.5 h), **FB2** (10 μM, 2 h), and FA (100 μM, 2 h) at 37°C. The cells were washed with PBS, trypsinized, and centrifuged at 3000 rpm for 5 min. The collected cells were dispersed in purified water (1 mL), and repeatedly frozen and thawed five times by liquid nitrogen to destroy the membrane structure. After removal of water under reduced pressure, the cell lysate was dissolved in methanol for analysis by high-resolution mass spectrometry.

1.12 Cell viabilities assay

PC12 Cells were seeded in a 96-well plate at a density of 2 × 10^4 per well at 37 °C and 5% CO_2. After 24 h of incubation, PC12 cells were treated with hemin/FA/**FB1**/**FB2** at varied concentrations for 24 h, respectively. After removal of the medium, MTT (100 μL, 0.1 mg mL^{-1}) in DMEM medium supplemented with 10% FBS was added and incubated for another 4 h to allow the formation of formazan precipitates by living cells. DMSO (100 μL) was then added to each well to dissolve the purple formazan. The absorption intensities at 565 nm and 720 nm in each well were recorded by a microplate reader to calculate the cell viabilities.

2. Substrate synthesis and characterization

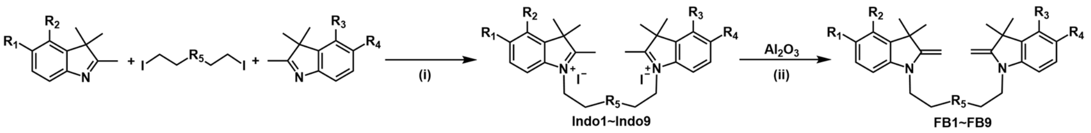

Figure S1. Reagents and conditions: (i) Dioxane/MeCN/$MeNO_2$, reflux. (ii) neutral alumina column chromatography.

2.1 Preparation of Indo1−Indo9

Preparation of 1,1'-(hexane-1,6-diyl)bis(2,3,3-trimethyl-3H-benzo[e]indolium iodide) (Indo1).

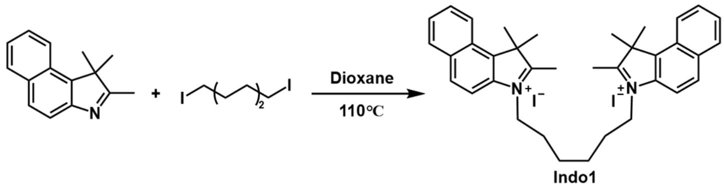

The mixture of 2,3,3-trimethyl-3H-benz[e]indole (9432 mg, 45 mmol, 2.25 eq.) and diiodohexane (6770 mg, 20 mmol, 1 eq.) in dioxane (60 mL) was refluxed in the dark at 110°C for 42 h under argon atmosphere. After filtration, the crude product was purified by recrystallization in MeCN/diethyl ether to afford a light gray solid **Indo1** (8613 mg, 57%).

^{1}H NMR (400 MHz, DMSO-*d6*): δ(ppm)= 8.40-8.35 (d, *J* = 8.4 Hz, 2H), 8.28 (d, *J* = 8.9 Hz, 2H), 8.22 (dd, *J* = 8.1, 1.6 Hz, 2H), 8.14 (d, *J* = 8.9 Hz, 2H), 7.79 (ddd, *J* = 8.4, 6.8, 1.5 Hz, 2H), 7.74 (ddd, *J* = 8.1, 6.8, 1.2 Hz, 2H), 4.59 (t, *J* = 7.7 Hz, 4H), 2.95 (s, 6H), 1.97-1.86 (m, 4H), 1.76 (s, 12H), 1.58-1.49 (m, 4H); **^{13}C NMR** (101 MHz, DMSO-*d6*): δ(ppm)= 196.30, 138.45, 136.98, 133.01, 130.64, 129.69, 128.44, 127.30, 127.21, 123.42, 113.30, 55.49, 47.75, 27.22, 25.51, 21.62, 13.98; **MS(ESI):** 251.30.

Preparation of 2,3,3-trimethyl-1-(6-(2,3,3-trimethyl-5-((6-(triphenylphosphonium iodide)-hexyl)oxy)-3H-indolium iodide-1-yl)hexyl)-3H-benzo[e]indolium iodide (Indo2).

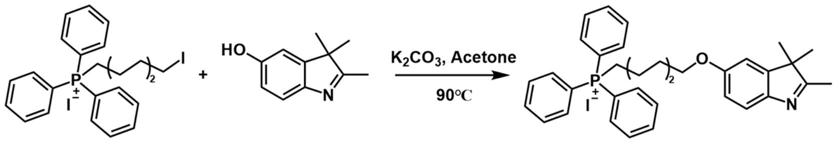

TPP-C6 TPP-Indo

Synthesis of TPP-Indo: The mixture of 6-iodohexyl-triphenylphosphonium iodide **TPP-C6** (2088 mg, 3.5 mmol, 1 eq.), 2,3,3-trimethyl-3H-5-hydroxyindole (701 mg, 4.00 mmol, 1.1 eq.), and potassium carbonate (1971 mg, 14.3, 4 eq.) in acetone (50 mL) was refluxed at 90°C for 15 h in the dark. After purification by silica column chromatography with DCM/methanol (*V*/*V*, 100:2) as eluent, **TPP-Indo** was obtained as gray solid (1642 mg, 73%).

^{1}H NMR (400 MHz, $CDCl_3$): δ(ppm)= 7.86-7.66 (m, 21H), 7.37 (d, *J* = 8.4 Hz, 2H), 6.84 (d, *J* = 2.4 Hz, 2H), 6.74 (dd, *J* = 8.3, 2.6 Hz, 2H), 3.93 (t, *J* = 6.2 Hz, 4H), 3.79-3.69 (m, 4H), 2.23 (s, 6H), 1.81-1.71 (m, 12H), 1.55-1.45 (m, 4H), 1.27 (s, 12H); **^{13}C NMR** (101 MHz, $CDCl_3$): δ(ppm)= 185.88, 157.41, 147.32, 147.16, 135.21, 135.18, 133.78, 133.68, 130.69, 130.57, 119.99, 118.57, 117.72, 112.94, 108.58, 68.20, 53.86, 30.21, 30.05, 28.98, 25.73, 23.40, 23.28, 15.33; **MS(ESI):** 520.25.

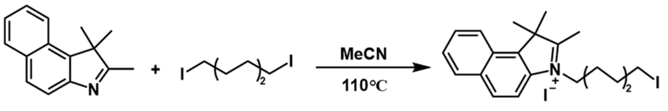

Indo-C6

Synthesis of Indo-C6: The mixture of 2,3,3-trimethyl-3H-benz[e]indole (2100 mg, 10 mmol, 1 eq.) and diiodohexane (10403 mg 30 mmol, 3 eq.) in acetonitrile (5 mL) was refluxed in the dark at 110°C for 60 h under argon atmosphere. After the reaction, the crude product was purified by silica column chromatography with DCM/methanol (*V*/*V*, 100:2.5) as eluent to obtain **Indo-C6** as a light green solid (3290 mg, 60%).

^{1}H NMR (400 MHz, DMSO-d_6): δ(ppm)= 8.37 (dd, *J* = 8.4, 1.2 Hz, 1H), 8.29 (d, *J* = 8.8 Hz, 1H), 8.22 (dd, *J* = 8.0, 1.6 Hz, 1H), 8.15 (d, *J* =9.0 Hz, 1H), 7.79 (ddd, *J* = 8.4, 6.9, 1.7 Hz, 1H), 7.73 (ddd, *J* = 8.2, 6.8, 1.2 Hz, 1H), 4.56 (t, *J* = 7.7 Hz, 2H), 3.28 (t, *J* = 6.9 Hz, 2H), 2.94 (s, 3H), 1.95-1.85 (m, 2H), 1.82-1.73 (m, 8H), 1.53-1.35 (m, 4H); **^{13}C NMR** (101 MHz, DMSO-d_6): δ(ppm)= 196.31, 138.47, 136.96, 133.02, 130.67, 129.70, 128.40, 127.26, 127.22, 123.41, 113.32, 55.48, 47.78, 32.54, 29.45, 27.23, 24.73, 21.62, 13.88, 8.85; **MS(ESI):** 420.05.

Synthesis of Indo2.

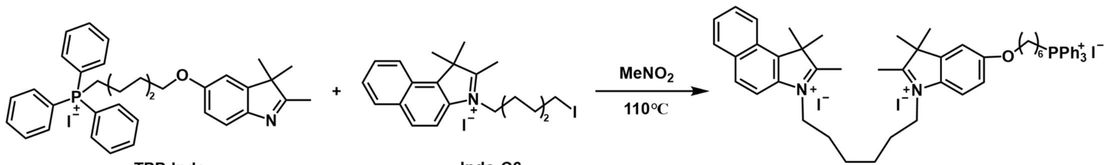

TPP-Indo Indo-C6 Indo2

The mixture of **TPP-Indo** (1417 mg, 2.2 mmol, 1 eq.) and **Indo-C6** (1523 mg, 2.8 mmol, 1.3 eq.) was dissolved in nitromethane (10 mL) and refluxed in the dark at 110°C for 20 h under argon atmosphere. After the reaction, the crude product was purified by silica column chromatography with DCM/methanol (*V*/*V*, 100:7.5) as eluent to afford a light green solid **Indo2** (1304 mg, 50%).

^{1}H NMR (400 MHz, DMSO-d_6): δ(ppm)= 8.38 (d, *J* = 8.3 Hz, 1H), 8.29 (d, *J* = 8.9 Hz, 1H), 8.22 (d, *J* = 8.4 Hz, 1H), 8.14

(d, J = 8.9 Hz, 1H), 7.94-7.70 (m, 18H), 7.44 (d, J = 2.6 Hz, 1H), 7.07 (dd, J = 8.9, 2.6 Hz, 1H), 4.58 (t, J = 7.8 Hz, 2H), 4.40 (t, J = 7.6 Hz, 2H), 4.01 (t, J = 6.4 Hz, 2H), 3.66-3.53 (m, 2H), 2.95 (s, 3H), 2.77 (s, 3H), 1.95-1.86 (m, 2H), 1.85-1.79 (m, 2H), 1.76 (s, 6H), 1.71-1.65 (m, 2H), 1.58-1.41 (m, 16H); **^{13}C NMR** (101 MHz, DMSO-d_6): δ(ppm)= 196.30, 193.36, 159.94, 143.97, 138.46, 136.97, 134.90, 134.87, 134.06, 133.63, 133.53, 133.01, 130.64, 130.29, 130.17, 129.69, 128.43, 127.28, 127.21, 123.42, 118.95, 118.10, 116.45, 114.63, 113.35, 109.84, 68.34, 55.49, 53.94, 47.78, 47.50, 29.62, 29.45, 28.24, 27.23, 27.09, 25.50, 24.69, 22.12, 21.76, 21.72, 21.64, 14.08, 13.94; **MS(ESI):** 271.25.

Preparation of 2,3,3-trimethyl-1-(6-(2,3,3-trimethyl-3H-indolium iodide-1-yl)hexyl)-3H-benzo[e]indolium iodide (Indo3).

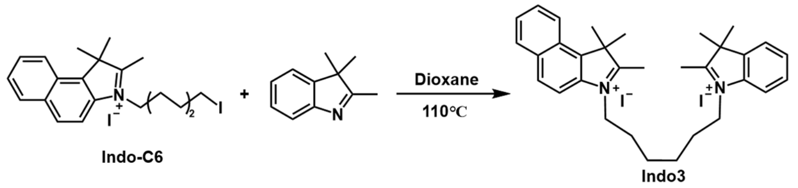

The mixture of 2,3,3-trimethyl-3H-indole (1566 mg, 9.8 mmol, 5.5 eq.) and **Indo-C6** (985 mg, 1.8 mmol, 1 eq.) in dioxane (20 mL) was refluxed in the dark at 110°C for 35 h under argon atmosphere. After the reaction, the crude product was obtained by filtration and washed with MeCN/diethyl to afford a light brown solid **Indo3** (922 mg, 72%).

1H NMR (400 MHz, DMSO-d6): δ(ppm)= 8.38 (d, J = 7.3 Hz, 1H), 8.29 (d, J = 9.0 Hz, 1H), 8.22 (dd, J = 8.1, 2.4 Hz, 1H), 8.15 (d, J = 8.9 Hz, 1H), 7.98-7.94 (m, 1H), 7.86-7.70 (m, 3H), 7.64-7.60 (m, 1H), 4.58 (t, J = 7.8 Hz, 2H), 4.46 (t, J = 7.8 Hz, 2H), 2.94 (s, 3H), 2.83 (s, 3H), 1.92-1.81 (m, 4H), 1.76 (s, 6H), 1.57-1.44 (m, 10H); **^{13}C NMR** (101 MHz, DMSO-d_6): δ(ppm)= 196.47, 196.31, 141.88, 141.03, 138.46, 137.00, 133.03, 130.66, 129.71, 129.46, 128.92, 128.46, 127.32, 127.23, 123.56, 123.42, 115.44, 113.29, 55.50, 54.17, 47.73, 47.48, 27.21, 27.00, 25.55, 25.49, 22.02, 21.62, 14.06, 13.89; **MS(ESI):** 226.25.

Preparation of 1,1'-(hexane-1,6-diyl)bis(2,3,3-trimethyl-3H-5-methoxy-indolium iodide) (Indo4).

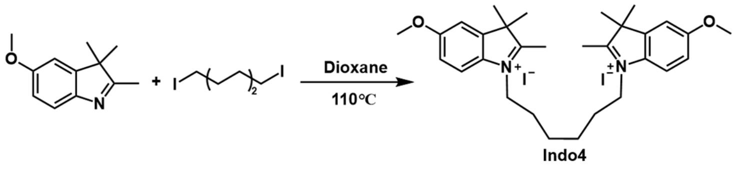

The mixture of 2,3,3-trimethyl-3H-5-methoxy indole (3535 mg, 18.7 mmol, 5 eq.) and diiodohexane (1332 mg, 3.9 mmol, 1 eq.) in dioxane (40 mL) was refluxed in the dark at 110°C for 38 h under argon atmosphere. After the reaction, the crude product was purified by silica column chromatography with DCM/methanol (V/V, 100:5) as eluent to obtain **Indo4** as a light green solid (1570 mg, 57%).

^{1}H NMR (400 MHz, DMSO-d_6): δ(ppm)= 7.86 (d, J = 8.9 Hz, 2H), 7.49 (d, J = 2.6 Hz, 2H), 7.13 (dd, J = 8.9, 2.6 Hz, 2H), 4.41 (t, J = 7.6 Hz, 4H), 3.86 (s, 6H), 2.77 (s, 6H), 1.88-1.75 (M, 4H), 1.51 (s, 12H), 1.48-1.41 (s, 4H); **^{13}C NMR** (101 MHz, DMSO-d_6): δ(ppm)= 193.45, 160.62, 144.00, 134.20, 116.43, 114.38, 109.38, 56.17, 53.96, 47.49, 27.08, 25.47, 22.12, 13.88; **MS(ESI):** 231.20.

Preparation of 1,1'-(hexane-1,6-diyl)bis(2,3,3-trimethyl-3H-indolium iodide) (Indo5).

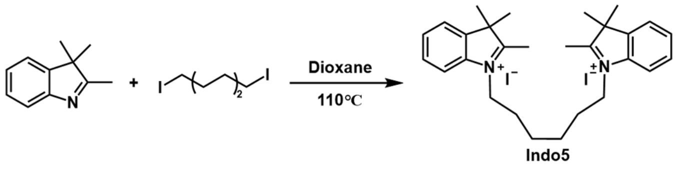

The mixture of 2,3,3-trimethyl-3H-indole (3782 mg, 24 mmol, 6 eq.) and diiodohexane (1376 mg, 4 mmol, 1 eq.) in dioxane (40 mL) was refluxed in the dark at 110°C for 32 h under argon atmosphere. After the reaction, the crude

product was obtained by filtration and purified by recrystallization in MeCN/diethyl ether to afford a light red solid **Indo5** (1346 mg, 51%).

^{1}H NMR (400 MHz, DMSO-*d_6*): δ(ppm)= 7.98-7.93 (m, 2H), 7.88-7.81 (m, 2H), 7.65-7.60 (m, 4H), 4.45 (t, *J* = 7.8 Hz, 4H), 2.83 (s, 6H), 1.89-1.78 (m, 4H), 1.56-1.44 (m, 16H); **^{13}C NMR** (101 MHz, DMSO-*d_6*): δ(ppm)= 196.45, 141.87, 141.02, 129.43, 128.91, 123.55, 115.46, 54.16, 47.53, 26.99, 25.49, 22.04, 14.21; **MS(ESI):** 201.20.

Preparation of 1,1'-(dodecane-1,12-diyl)bis(2,3,3-trimethyl-3H-indolium iodide) (Indo6).

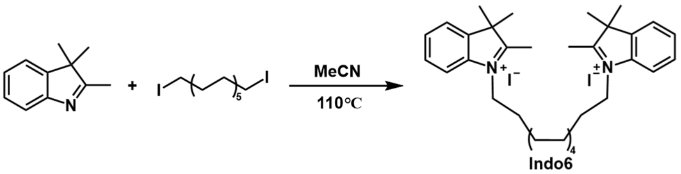

The mixture of 2,3,3-trimethyl-3H-indole (2790 mg, 17.6 mmol, 5 eq.) and 1,12-diiodododecane (1475 mg, 3.5 mmol, 1 eq.) in acetonitrile (20 mL) was refluxed in the dark at 90°C for 24 h under argon atmosphere. After the reaction, the crude product was obtained by filtration and washed with MeCN/diethyl to afford a light red solid **Indo6** (1300 mg, 50%).

^{1}H NMR (400 MHz, DMSO-*d_6*): δ(ppm)= 7.99-7.94 (m, 2H), 7.86-7.82 (m, 2H), 7.65-7.60 (m, 4H), 4.44 (t, *J* = 7.8 Hz, 4H), 2.83 (s, 6H), 1.88-1.76 (m, 4H), 1.53 (s, 12H), 1.44-1.35 (m, 4H), 1.29-1.17 (m, 12H); **^{13}C NMR** (101 MHz, DMSO-*d_6*): δ(ppm)= 196.42, 141.88, 141.05, 129.40, 128.93, 123.53, 115.48, 54.14, 47.60, 28.91, 28.82, 28.62, 27.24, 25.89, 22.01, 14.04; **MS(ESI):** 243.25.

Preparation of 1,1'-(octadecane-1,18-diyl)bis(2,3,3-trimethyl-3H-indolium iodide) (Indo7).

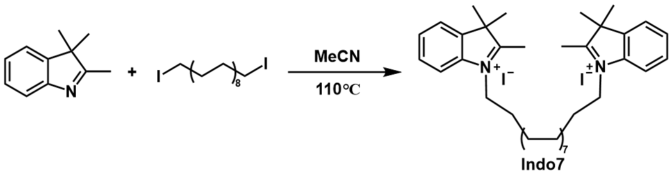

The mixture of 2,3,3-trimethyl-3H-indole (386 mg, 2.4 mmol, 5 eq.) and 1,18-dibromooctadecane (242.7 mg, 0.49 mmol, 1 eq.) in acetonitrile (20 mL) was refluxed in the dark at 90°C for 24 h under argon atmosphere. After the reaction, the crude product was obtained by filtration and washed with MeCN/diethyl ether to afford a light red solid **Indo7** (224 mg, 55%).

^{1}H NMR (400 MHz, DMSO-*d_6*): δ(ppm)= 7.99-7.94 (m, 2H), 7.86-7.81 (m, 2H), 7.65-7.60 (m, 4H), 4.44 (t, *J* = 7.7 Hz, 4H), 2.83 (s, 6H), 1.88-1.77 (m, 4H), 1.53 (s, 12H), 1.45-1.35 (m, 4H), 1.26-1.18 (m, 24H); **^{13}C NMR** (101 MHz, DMSO-*d_6*): δ(ppm)= 196.39, 141.87, 141.04, 129.42, 128.94, 123.53, 115.46, 54.15, 47.60, 29.07, 29.02, 28.94, 28.94, 28.81, 28.60, 27.23, 25.88, 22.02, 14.02; **MS(ESI):** 285.46.

Preparation of 1,1'-(1,2-bis(2-ethoxy)-ethane)bis(2,3,3-trimethyl-3H-benzo[e]indolium iodide) (Indo8).

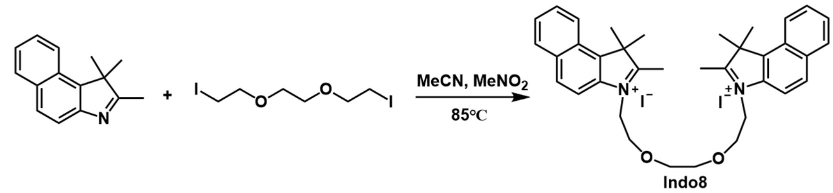

The mixture of 2,3,3-trimethyl-3H-benz[e]indole (4181 mg, 20.0 mmol, 5 eq.) and 1,2-bis(2-iodoethoxy) ethane (1467 mg, 4.0 mmol, 1 eq.) in acetonitrile/nitromethane (*V*/*V* =1: 1, 20 mL) was refluxed in the dark at 85°C for 17.5 h under argon atmosphere. After the reaction, the crude product was purified by silica column chromatography with DCM/methanol (*V*/*V*, 100:5) as eluent to obtain **Indo8** as a light green solid (2308 mg, 74%).

^{1}H NMR (400 MHz, DMSO-*d_6*): δ(ppm)= 8.30 (dd, *J* = 8.3, 1.4 Hz, 2H), 8.23 (d, *J* = 8.9 Hz, 2H), 8.19 (dd, *J* = 7.7, 1.6 Hz, 2H), 8.05 (d, *J* = 8.9 Hz, 2H), 7.80-7.70 (m, 4H), 4.76 (t, *J* = 5.2 Hz, 4H), 3.82 (t, *J* = 5.1 Hz, 4H), 3.40 (s, 4H), 2.82 (s,

6H), 1.67 (s, 12H); **^{13}C NMR** (101 MHz, DMSO-d_6): δ(ppm)= 197.89, 138.33, 136.63, 132.95, 130.48, 129.66, 128.44, 127.29, 127.10, 123.33, 113.35, 69.80, 66.87, 55.49, 47.99, 21.45, 14.32; **MS(ESI):** 267.25.

Preparation of 1,1'-(dodecane-1,12-diyl)bis(2,3,3-trimethyl-3H-benzo[e]indolium iodide) (Indo9).

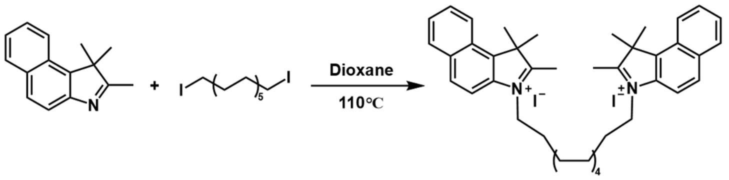

The mixture of 2,3,3-trimethyl-3H-benz[e]indole (4039 mg, 19 mmol, 6 eq.) and 1,12-diiodododecane (1350 mg, 3.2 mmol, 1 eq.) in acetonitrile/nitromethane (*V*/*V* = 5: 1, 30 mL) was refluxed in the dark at 85°C for 96 h under argon atmosphere. After the reaction, the crude product was purified by silica column chromatography with DCM/methanol (*V*/*V*, 100:5) as eluent to obtain **Indo9** as a light green solid (2394 mg, 89%).

^{1}H NMR (400 MHz, DMSO-d_6): δ(ppm)= 8.37 (d, *J* = 8.4 Hz, 2H), 8.28 (d, *J* = 8.8 Hz, 2H), 8.21 (dd, *J* = 8.3, 1.7 Hz, 2H), 8.13 (d, *J* = 8.9 Hz, 2H), 7.79 (ddd, *J* = 8.6, 7.0, 1.5 Hz, 2H), 7.73 (ddd, *J* = 8.2, 6.8, 1.2 Hz, 2H), 4.56 (t, *J* = 7.8 Hz, 4H), 2.93 (s, 6H), 1.93-1.80 (m, 4H), 1.76 (s, 12H), 1.47-1.36 (m, 4H), 1.35-1.16 (m, 12H); **^{13}C NMR** (101 MHz, DMSO-d_6): δ(ppm)= 196.27, 138.48, 136.95, 133.01, 130.66, 129.70, 128.39, 127.24, 127.21, 123.41, 113.37, 55.46, 47.87, 28.89, 28.80, 28.64, 27.45, 25.86, 21.61, 13.95; **MS(ESI):** 293.40.

2.2 Preparation of FB1−FB9

Preparation of 1,1'-(hexane-1,6-diyl)bis(3,3-dimethyl-2-methylene-benzo[e]indolin) (FB1).

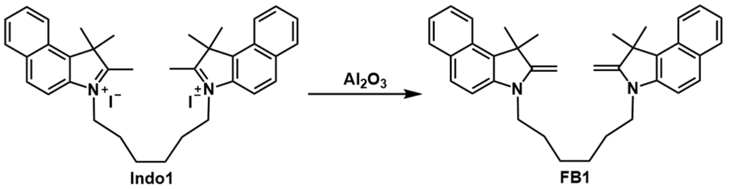

Indo1 (1715 mg, 2.27 mmol) and an appropriate amount of neutral Al_2O_3 (200-300 mesh) were mixed in DCM and subjected to an Al_2O_3 column with DCM/methanol (*V*/*V*, 100:5) as eluent to obtain a green solid **FB1**(742 mg, 83%). Recrystallization in n-pentane/DCM yielded light green crystal (527 mg, 46%).

^{1}H NMR (400MHz, DMSO-*d6*-$CDCl_3$): δ(ppm)= 7.90 (d, *J* = 8.6 Hz, 2H), 7.70 (d, *J* = 8.2 Hz, 2H), 7.60 (d, *J* = 8.8 Hz, 2H), 7.32 (m, 2H), 7.11 (m, 2H), 7.00 (d, *J* = 8.7 Hz, 2H), 3.90 (d, *J* = 2.2 Hz, 2H), 3.88 (d, *J* = 2.3 Hz, 2H), 3.56 (t, *J* = 7.2 Hz, 4H), 1.65-1.51 (m, 16H), 1.39 (m, 4H); **^{13}C NMR** (101 MHz, DMSO-*d6*-$CDCl_3$): δ(ppm)= 162.09, 143.10, 129.37, 129.22, 128.65, 128.14, 126.11, 125.62, 120.92, 120.84, 108.75, 73.59, 45.34, 41.31, 29.38, 26.16, 25.57; **MS(ESI):** 501.30.

Preparation of 3,3-dimethyl-2-methylene-1-(6-(3,3-dimethyl-2-methylene-5-((6-(triphenylphosphonium iodide)-hexyl)oxy)-indolin-1-yl)hexyl)-benzo[e]indolin (FB2).

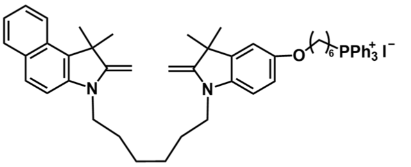

Indo2 (592 mg, 0.50 mmol) and an appropriate amount of neutral Al_2O_3 (200-300 mesh) were mixed in DCM and subjected to an Al_2O_3 column with DCM/methanol (*V*/*V*, 100:3) as eluent to obtain a colorless solid **FB2** (342 mg, 74%).

^{1}H NMR (400MHz, DMSO-*d6*): δ(ppm)= 7.95-7.73 (m, 17H), 7.71 (d, *J* = 8.6 Hz, 1H), 7.37 (ddd, *J* = 8.6, 6.7, 1.3 Hz, 1H), 7.17-7.10 (m, 2H), 6.76 (d, *J* = 2.5 Hz, 1H), 6.53 (dd, *J* = 8.4, 2.5 Hz, 1H), 6.43 (d, *J* = 8.5 Hz, 1H), 3.94 (dd, *J* = 11.0,

1.8 Hz, 2H), 3.79 (t, *J* = 6.3, 2H), 3.74 (dd, *J* = 11.4, 1.8 Hz, 2H), 3.62-3.62 (m, 4H), 3.41 (t, *J* = 7.1 Hz, 2H), 1.64-1.47 (m, 16H), 1.46-1.38 (m, 2H), 1.38-1.32 (m, 4H), 1.26-1.19 (m, 8H); **^{13}C NMR** (101 MHz, DMSO-*d6*): δ(ppm)= 162.19, 160.92, 152.12, 143.31, 139.56, 138.23, 134.89, 134.86, 133.63, 133.53, 130.29, 130.17, 129.46, 128.94, 128.28, 126.47, 125.71, 121.20, 121.08, 118.99, 118.14, 112.24, 110.41, 109.20, 104.96, 73.97, 72.71, 67.81, 45.46, 43.92, 41.35, 41.25, 29.71, 29.52, 28.59, 26.16, 26.12, 25.65, 25.25, 24.83, 21.78, 21.74, 21.06; **MS(ESI):** 406.40.

Preparation of 3,3-dimethyl-2-methylene-1-(6-(3,3-dimethyl-2-methyleneindolin-1-yl)hexyl)-benzo[e] indolin (FB3).

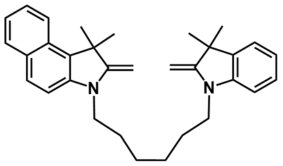

FB3

Indo3 (888 mg, 1.26 mmol) and an appropriate amount of neutral Al_2O_3 (200-300 mesh) were mixed in DCM and subjected to an Al_2O_3 column with DCM/methanol (*V*/*V*, 100:1.5) as eluent to obtain a light yellow liquid **FB3** (300 mg, 53%).

^{1}H NMR (400MHz, $CDCl_3$): δ(ppm)= 7.94 (d, *J* = 8.9 Hz, 1H), 7.74 (d, *J* = 8.8 Hz, 1H), 7.65 (d, *J* = 8.7 Hz, 1H), 7.37 (ddd, *J* = 8.7, 6.7, 1.3 Hz, 1H), 7.16 (t, *J* = 7.0 Hz, 1H), 7.12-7.04 (m, 2H), 6.93 (d, *J* = 8.7 Hz, 1H), 6.74 (t, *J* = 7.0 Hz, 1H), 6.49 (d, *J* = 7.6 Hz, 1H), 3.91 (dd, *J* = 14.3, 2.1 Hz, 2H), 3.82 (dd, *J* = 11.9, 2.1 Hz, 2H), 3.57 (t, *J* = 7.3 Hz, 2H), 3.47 (t, *J* = 7.3 Hz, 2H), 1.71-1.61 (m, 10H), 1.45-1.38 (m, 4H), 1.32 (s, 6H); **^{13}C NMR** (101 MHz, $CDCl_3$): δ(ppm)= 163.55, 161.78, 146.09, 143.63, 137.68, 130.14, 129.75, 129.09, 128.88, 127.61, 126.80, 126.45, 121.94, 121.59, 121.48, 118.31, 108.91, 105.16, 73.14, 73.02, 46.29, 44.31, 42.39, 42.29, 30.20, 29.98, 27.24, 27.21, 26.44, 26.22; **MS(ESI):** 451.25.

Preparation of 1,1'-(hexane-1,6-diyl)bis(3,3-dimethyl-2-methylene-5-methoxy-indolium iodide) (FB4).

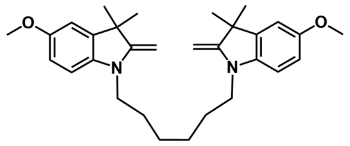

FB4

Indo4 (506 mg, 0.70 mmol) and an appropriate amount of neutral Al_2O_3 (200-300 mesh) were mixed in DCM and subjected to an Al_2O_3 column with DCM/methanol (*V*/*V*, 100:2) as eluent to obtain a light purple liquid **FB4** (231 mg, 71%).

^{1}H NMR (400MHz, DMSO-*d6*): δ(ppm)= 6.81 (d, *J* = 2.5 Hz, 2H), 6.62 (dd, *J* = 8.4, 2.5 Hz, 2H), 6.48 (d, *J* = 8.4 Hz, 2H), 3.77 (d, *J* = 1.8 Hz, 2H), 3.74 (d, *J* = 1.6 Hz, 2H), 3.67 (s, 6H), 3.42 (t, *J* = 7.2 Hz, 4H), 1.55-1.45 (m, 4H), 1.35-1.28 (m, 4H), 1.24 (s, 12H); **^{13}C NMR** (101 MHz, DMSO-*d6*): δ(ppm)= 160.98, 152.86, 139.59, 138.26, 111.64, 109.78, 105.03, 72.66, 55.52, 43.95, 41.39, 29.72, 26.17, 25.27; **MS(ESI):** 461.20.

Preparation of 1,1'-(hexane-1,6-diyl)bis(3,3-dimethyl-2-methyleneindolin) (FB5).

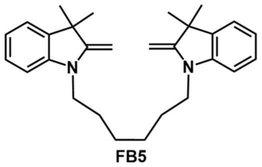

FB5

Indo5 (1100 mg, 1.68 mmol) and an appropriate amount of neutral Al_2O_3 (200-300 mesh) were mixed in DCM and subjected to an Al_2O_3 column with DCM/methanol (*V*/*V*, 100:4) as eluent to obtain a light red liquid **FB5** (450 mg, 67%).

^{1}H NMR (400MHz, DMSO-*d6*): δ(ppm)= 7.12 (dd, *J* = 7.3, 1.3 Hz, 2H), 7.04 (td, *J* = 7.7, 1.3 Hz, 2H), 6.67 (td, *J* = 7.4, 1.0 Hz, 2H), 6.59 (d, *J* = 7.7 Hz, 2H), 3.85 (d, *J* = 1.7 Hz, 2H), 3.81 (d, *J* = 1.8 Hz, 2H), 3.47 (t, *J* = 7.2 Hz, 4H), 1.56-1.47 (m, 4H), 1.37-1.30 (m, 4H), 1.24 (s, 12H); **^{13}C NMR** (101 MHz, DMSO-*d6*): δ(ppm)= 160.55, 145.51, 136.91, 127.47,

121.76, 118.03, 105.20, 73.63, 43.60, 41.17, 29.79, 26.17, 25.37; **MS(ESI):** 401.25

Preparation of 1,1'-(dodecane-1,12-diyl)bis(3,3-dimethyl-2-methylene-indolin) (FB6).

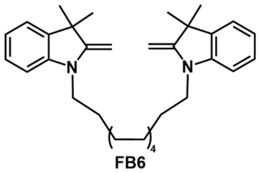

FB6

Indo6 (974 mg, 1.32 mmol) and an appropriate amount of neutral Al_2O_3 (200-300 mesh) were mixed in DCM and subjected to an Al_2O_3 column with DCM/methanol (*V*/*V*, 100:1.5) as eluent to obtain a light red liquid **FB6** (167 mg, 26%).

^{1}H NMR (400MHz, $CDCl_3$): δ(ppm)= 7.14-7.06 (m, 4H), 6.74 (td, *J* = 7.4, 1.0 Hz, 2H), 6.52 (d, *J* = 7.7 Hz, 2H), 3.86 (s, 2H), 3.82 (s, 2H), 3.47 (t, *J* = 8.4 Hz, 4H), 1.65-1.57 (m, 4H), 1.35-1.22 (m, 28H); **^{13}C NMR** (101 MHz, $CDCl_3$): δ(ppm)= 161.82, 146.19, 137.72, 127.60, 121.91, 118.23, 105.18, 72.90, 44.32, 42.46, 30.20, 29.68, 29.61, 27.39, 26.21; **MS(ESI):** 485.30.

Preparation of 1,1'-(octadecane-1,18-diyl)bis(3,3-dimethyl-2-methylene-indolin) (FB7).

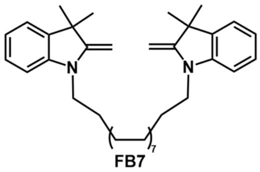

FB7

Indo7 (275 mg, 0.33 mmol) and an appropriate amount of neutral Al_2O_3 (200-300 mesh) were mixed in DCM and subjected to an Al_2O_3 column with DCM/methanol (*V*/*V*, 100:1) as eluent to obtain a light red liquid **FB7** (74 mg, 49%).

^{1}H NMR (400MHz, $CDCl_3$): δ(ppm)= 7.14-7.06 (m, 4H), 6.74 (td, *J* = 7.4, 1.0 Hz, 2H), 6.52 (d, *J* = 7.8 Hz, 2H), 3.85 (d, *J* = 1.9 Hz, 2H), 3.81 (d, *J* = 1.9 Hz, 2H), 3.47 (d, *J* = 7.4 Hz, 4H), 1.67-1.58 (m, 4H), 1.35-1.22 (m, 40H); **^{13}C NMR** (101 MHz, $CDCl_3$): δ(ppm)= 161.81, 146.18, 137.72, 127.59, 121.90, 118.23, 105.18, 72.90, 44.31, 42.46, 30.20, 29.82, 29.79, 29.75, 29.73, 29.63, 27.39, 26.20; **MS(ESI):** 569.70.

Preparation of 1,1'-(1,2-bis(2-ethoxy)-ethane)bis(3,3-dimethyl-2-methylene-benzo[e]indolin) (FB8).

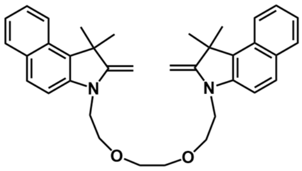

FB8

Indo8 (250 mg, 0.32 mmol) and an appropriate amount of neutral Al_2O_3 (200-300 mesh) were mixed in DCM and subjected to an Al_2O_3 column with DCM/methanol (*V*/*V*, 100:3) as eluent to obtain a light purple liquid **FB8** (120 mg, 71%).

^{1}H NMR (400MHz, DMSO-*d6*): δ(ppm)= 7.93 (dd, *J* = 8.7, 1.0 Hz, 2H), 7.74 (dd, *J* = 8.3, 1.5 Hz, 2H), 7.65 (d, *J* = 8.7 Hz, 2H), 7.37 (ddd, *J* = 8.7, 6.87, 1.5 Hz, 2H), 7.19-7.08 (m, 4H), 3.94 (d, *J* = 2.1 Hz, 2H), 3.91 (d, *J* = 2.1 Hz, 2H), 3.69 (t, *J* = 5.9 Hz, 4H), 3.55 (t, *J* = 5.8 Hz, 4H), 3.45 (s, 4H), 1.55 (s, 12H); **^{13}C NMR** (101 MHz, DMSO-*d6*): δ(ppm)= 162.15, 143.55, 129.42, 129.34, 128.69, 128.39, 126.41, 125.53, 121.25, 121.11, 109.66, 74.26, 70.20, 66.79, 45.42, 42.05, 29.47; **MS(ESI):** 533.30.

Preparation of 1,1'-(dodecane-1,12-diyl)bis(3,3-dimethyl-2-methylene-benzo[e]indolin) (FB9).

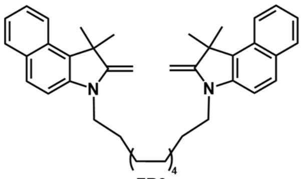

Indo9 (270 mg, 0.29 mmol) and an appropriate amount of neutral Al_2O_3 (200-300 mesh) were mixed in DCM and subjected to an Al_2O_3 column with DCM/methanol (*V*/*V*, 100:1.5) as eluent to obtain a light green solid **FB9** (170 mg, 91%).

^{1}H NMR (400MHz, $CDCl_3$): δ(ppm)= 7.94 (dd, *J* = 8.6, 1.0 Hz, 2H), 7.74 (dd, *J* = 8.3, 1.7 Hz, 2H), 7.67 (d, *J* = 8.6 Hz, 2H), 7.37 (ddd, *J* = 8.6, 6.7, 1.4 Hz, 2H), 7.16 (ddd, *J* = 8.3, 6.7, 1.1 Hz, 2H), 6.96 (d, *J* = 8.7 Hz, 2H), 3.93 (d, *J* = 2.1 Hz, 2H), 3.91 (d, *J* = 2.1 Hz, 2H), 3.57 (t, *J* = 7.4 Hz, 4H), 1.70-1.61 (m, 16H), 1.39-1.20 (m, 16H); **^{13}C NMR** (101 MHz, $CDCl_3$): δ(ppm)= 163.60, 143.74, 130.15, 129.75, 129.06, 128.86, 126.79, 126.44, 121.59, 121.44, 108.98, 73.03, 46.28, 42.55, 29.67, 29.66, 29.61, 27.35, 26.46; **MS(ESI):** 585.60.

2.3 Preparation of Cy3

Preparation of aHCY-1.

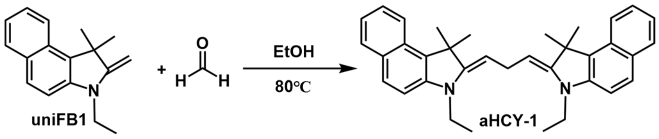

1-ethyl-2-methylene-3,3-dimethyl-benzo[e]indolin (**uniFB1**) was synthesized as reported previously.[5] The mixture of **uniFB1** (892 mg, 3.74 mmol, 2.5 eq.) and FA (114 μL, 1.5 mmol, 1 eq.) in EtOH (20 mL) was refluxed in the dark at 80°C for 2 h under argon atmosphere. After the reaction, the mixture was filtered to obtain **aHCY-1** as a light yellow solid (618 mg, 85%).

^{1}H NMR (400 MHz, $CDCl_3$): δ(ppm)= 7.97 (d, *J* = 8.4 Hz, 2H), 7.76 (d, *J* = 7.6 Hz, 2H), 7.69 (d, *J* = 8.7 Hz, 2H), 7.43-7.37 (m, 2H), 7.17 (t, *J* = 7.5 Hz, 2H), 6.92 (d, *J* = 8.7 Hz, 2H), 4.42 (t, *J* = 7.6 Hz, 2H), 3.65 (q, *J* = 7.0 Hz, 4H), 3.59 (t, *J* = 7.7 Hz, 2H), 1.95 (s, 12H), 1.21 (t, *J* = 7.0 Hz, 6H); **^{13}C NMR** (101 MHz, $CDCl_3$): δ(ppm)= 152.83, 143.37, 129.92, 129.68, 129.28, 128.87, 126.61, 126.34, 121.83, 121.52, 120.92, 108.75, 94.92, 45.14, 37.49, 26.73, 11.14; MS(ESI): 487.30.

Preparation of aCY-1.

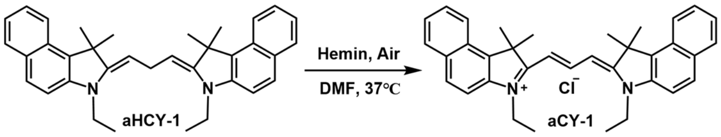

The mixture of **aHCY-1** (50.0 mg,1 mM) and hemin (1 mM) in DMF was stirred at 37°C in the dark for 12 h. After the reaction, the crude product was purified by Al_2O_3 column chromatography with DCM/methanol (*V*/*V*, 100:4) as eluent to obtain **aCY-1** (44.5 mg, 84%).

R_f: 0.25 (Al_2O_3, DCM/methanol 10: 1).

^{1}H NMR (400 MHz, CD_3OD-*d4*): δ(ppm)= 8.78 (t, *J* = 13.5 Hz, 1H), 8.29 (d, *J* = 8.5 Hz, 2H), 8.05 (d, *J* = 8.8 Hz, 2H), 8.02 (d, *J* = 8.2 Hz, 2H), 7.71-7.62 (m, 4H), 7.55-7.49 (m, 2H), 6.53 (d, *J* = 13.5 Hz, 2H), 4.34 (q, *J* = 7.2 Hz, 4H), 2.09 (s, 12H), 1.50 (t, *J* = 7.2 Hz, 6H); **^{13}C NMR** (101 MHz, CD_3OD-*d4*): δ(ppm)= 176.98, 150.91, 140.43, 135.01, 133.66, 132.02, 131.18, 129.38, 128.92, 126.34, 123.33, 111.98, 102.77, 52.43, 40.50, 27.91, 12.95; **HRMS-ESI:** $[M]^+$ m/z = 485.2948, calcd. for $[C_{35}H_{37}N_2^+]$: 485.2951.

Preparation of CY-1−CY-9 under standard conditions:

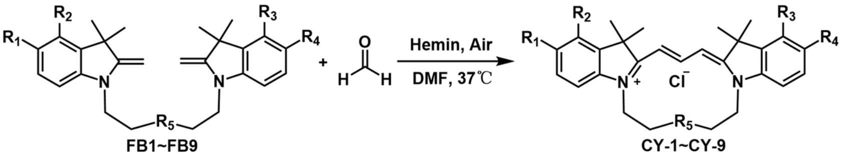

FB1−FB9 (1 mM, 1 eq.), 37% formaldehyde aqueous solution (5 mM, 5 eq.) and hemin (1 mM, 1 eq.) were dissolved in DMF and stirred at 37°C in the dark for 12 h. After the reaction, the crude product was purified by Al_2O_3 column chromatography with DCM/methanol (*V*/*V*, 100:3−100:4) as eluent to obtain **CY-1−CY-9**.

Preparation of CY-1.

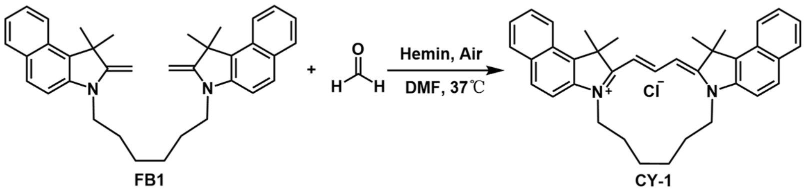

CY-1 (17 mg, 62%) was prepared from **FB1** according to the procedure described above.

R_f: 0.25 (Al_2O_3, DCM/methanol=10:1).

^{1}H NMR (400 MHz, CD_3OD-*d4*): δ(ppm)= 8.52 (t, *J* = 13.8 Hz, 1H), 8.20 (dd, *J* = 8.5, 1.0 Hz, 2H), 8.07 (d, *J* = 8.8 Hz, 2H), 8.01 (dd, *J* = 8.3, 1.4 Hz, 2H), 7.69 (d, *J* = 8.9 Hz, 2H), 7.64 (ddd, *J* = 8.6, 6.8, 1.4 Hz, 2H), 7.51 (ddd, *J* = 8.3, 6.9, 1.1 Hz, 2H), 6.38 (d, *J* = 13.8 Hz, 2H), 4.55-4.46 (m, 4H), 2.22-2.12 (m, 4H), 1.97-1.90 (m, 4H), 1.82 (s, 12H); **^{13}C NMR** (101 MHz, CD_3OD-*d4*): δ(ppm)= 177.03, 151.49, 141.95, 133.55, 133.32, 131.82, 130.94, 129.91, 128.83, 126.38, 123.64, 111.64, 102.48, 53.37, 47.94, 26.27, 24.94, 22.65; **HRMS-ESI:** $[M]^+$ m/z = 511.3101, calcd. for $[C_{37}H_{39}N_2^+]$: 511.3108.

Preparation of CY-2.

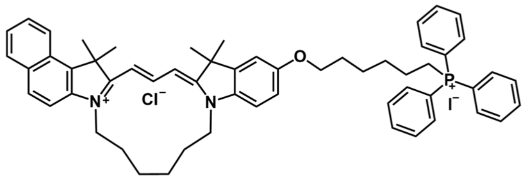

CY-2 (70 mg, 57%) was prepared from **FB2** according to the procedure described above.

R_f: 0.30 (Al_2O_3, DCM/methanol=10:1).

^{1}H NMR (400 MHz, CD_3OD-*d4*): δ(ppm)= 8.41 (t, *J* = 13.8 Hz, 1H), 8.18 (d, *J* = 8.4 Hz, 1H), 8.04 (d, *J* = 8.9 Hz, 1H), 7.99 (d, *J* = 7.9 Hz, 1H), 7.93-7.72 (m, 15H), 7.67 (d, *J* = 8.9 Hz, 1H), 7.62 (ddd, *J* = 8.4, 6.8, 1.3 Hz, 1H), 7.48 (t, *J* = 7.3 Hz, 1H), 7.34 (d, *J* = 8.8 Hz, 1H), 7.12 (d, *J* = 2.6 Hz, 1H), 6.97 (dd, *J* = 8.7, 2.6 Hz, 1H), 6.32 (d, *J* = 13.6 Hz, 1H), 6.27 (d, *J* = 13.8 Hz, 1H), 4.50-4.40 (m, 2H), 4.40-4.30 (m, 2H), 4.02 (t, *J* = 6.4 Hz, 2H), 3.52-3.41 (m, 2H), 2.17-2.02 (m, 4H), 1.94-1.84 (m, 4H), 1.81-1.62 (m, 12H), 1.59-1.49 (m, 8H); **^{13}C NMR** (101 MHz, CD_3OD-*d4*): δ(ppm)= 176.29, 175.04, 159.52, 151.01, 142.47, 142.02, 137.98, 136.27, 136.24, 134.90, 134.80, 133.37, 133.00, 131.72, 131.60, 131.48, 130.91, 129.93, 128.74, 126.17, 123.58, 120.40, 119.54, 115.54, 112.88, 111.60, 110.96, 102.70, 101.94, 69.60, 53.15, 51.93, 47.90, 47.84, 31.38, 31.22, 30.01, 26.72, 26.49, 26.43, 25.01, 24.85, 23.53, 23.01, 22.64, 22.50, 22.39; **HRMS-ESI:** $[M]^{2+}$ m/z = 411.2333, calcd. for $[C_{57}H_{63}N_2OP^{2+}]$: 411.2334.

Preparation of CY-3.

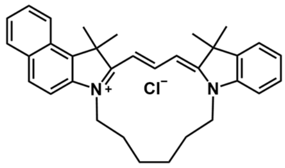

CY-3 (40 mg, 35%) was prepared from **FB3** according to the procedure described above.

R_f: 0.20 (Al_2O_3, DCM/methanol=10:1).

^{1}H NMR (400 MHz, CD_3OD-*d4*): δ(ppm)= 8.45 (t, *J* = 13.7 Hz, 1H), 8.19 (d, *J* = 8.6 Hz, 1H), 8.06 (d, *J* = 8.9 Hz, 1H), 8.00 (d, *J* = 7.5 Hz, 1H), 7.70 (d, *J* = 8.9 Hz, 1H), 7.62 (t, *J* = 7.7 Hz, 1H), 7.52-7.46 (m, 2H), 7.44 (d, *J* = 7.3 Hz,1H), 7.39 (d, *J* = 7.4 Hz, 1H), 7.29 (t, *J* = 7.4 Hz, 1H), 6.37 (d, *J* = 13.8 Hz, 1H), 6.24 (d, *J* = 13.7 Hz, 1H), 4.52-4.42 (m, 2H), 4.42-4.31 (m, 2H), 2.17-2.01 (m, 4H), 1.89 (s, 4H), 1.79 (s, 6H), 1.53 (s, 6H); **^{13}C NMR** (101 MHz, CD_3OD-*d4*): δ(ppm)= 177.42, 175.28, 151.85, 144.76, 141.84, 140.51, 133.56, 133.45, 131.85, 130.93, 129.90, 129.83, 128.84, 126.57, 126.45, 123.92, 123.66, 111.73, 102.85, 102.26, 53.45, 51.56, 47.99, 47.53, 26.86, 26.14, 24.97, 24.75, 22.59, 22.29; **HRMS-ESI:** $[M]^+$ m/z = 461.2942, calcd. for [$C_{33}H_{37}N_2^+$]: 461.2951.

Preparation of CY-4.

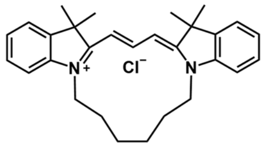

CY-4 (41 mg, 40%) was prepared from **FB4** according to the procedure described above.

R_f: 0.22 (Al_2O_3, DCM/methanol=10:1).

^{1}H NMR (400 MHz, CD_3OD-*d4*): δ(ppm)= 8.35 (t, *J* = 13.8 Hz, 1H), 7.30 (d, *J* = 8.9 Hz, 2H), 7.11 (d, *J* = 2.4 Hz, 2H), 6.99 (dd, *J* = 8.8, 2.5 Hz, 2H), 6.12 (d, *J* = 13.8 Hz, 2H), 4.38-4.26 (m, 4H), 3.84 (s, 6H), 2.15-2.01 (m, 4H), 1.86 (s, 4H), 1.51 (s, 12H); **^{13}C NMR** (101 MHz, CD_3OD-*d4*): δ(ppm)= 174.53, 159.99, 150.66, 142.28, 138.14, 114.80, 112.52, 110.40, 101.87, 56.42, 51.65, 47.70, 26.78, 24.86, 22.36; **HRMS-ESI:** $[M]^+$ m/z = 471.2995, calcd. for [$C_{31}H_{39}N_2O_2^+$]: 471.3006.

Preparation of CY-5.

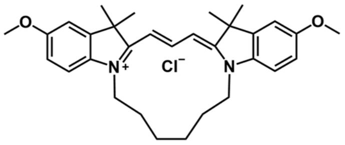

CY-5 (17 mg, 26%) was prepared from **FB5** according to the procedure described above.

R_f: 0.20 (Al_2O_3, DCM/methanol=10:1).

^{1}H NMR (400 MHz, CD_3OD-*d4*): δ(ppm)= 8.47 (t, *J* = 13.7 Hz, 1H), 7.50 (td, *J* = 7.3, 1.4 Hz, 2H), 7.49-7.40 (m, 4H), 7.30 (td, *J* = 7.3, 1.4 Hz, 2H), 6.23 (d, *J* = 13.8 Hz, 2H), 4.45-4.32 (m, 4H), 2.17-2.04 (m, 4H), 1.89 (s, 4H), 1.53 (s, 12H); **^{13}C NMR** (101 MHz, CD_3OD-*d4*): δ(ppm)= 175.85, 152.37, 144.71, 140.60, 129.94, 126.77, 123.95, 111.89, 102.57, 51.71, 47.62, 26.71, 24.84, 22.29; **HRMS-ESI:** $[M]^+$ m/z = 411.2784, calcd. for [$C_{29}H_{35}N_2^+$]: 411.2795.

Preparation of CY-6.

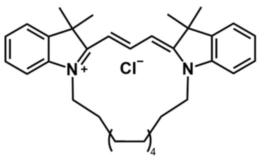

CY-6 (29 mg, 29%) was prepared from **FB6** according to the procedure described above.

R_f: 0.20 (Al_2O_3, DCM/methanol=10:1).

^{1}H NMR (400 MHz, CD_3OD-*d4*): δ(ppm)= 8.57 (t, *J* = 13.4 Hz, 1H), 7.56 (d, *J* = 7.5 Hz, 2H), 7.45 (t, *J*= 7.7 Hz, 2H), 7.39-7.30 (m, 4H), 6.40 (d, *J* = 13.4 Hz, 2H), 4.18 (t, *J* = 7.3 Hz, 4H), 1.94-1.84 (m, 4H), 1.78 (s, 12H), 1.55-1.48 (m, 8H), 1.46-1.37 (m, 8H); **^{13}C NMR** (101 MHz, CD_3OD-*d4*): δ(ppm)= 176.33, 151.77, 143.02, 142.29, 130.03, 126.84, 123.54, 112.62, 103.58, 50.77, 44.52, 30.05, 29.59, 29.06, 28.34, 27.38, 26.60; **HRMS-ESI:** $[M]^+$ m/z = 495.3718, calcd. for [$C_{35}H_{47}N_2^+$]: 495.3734.

Preparation of CY-7.

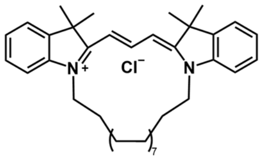

CY-7 (17 mg, 22%) was prepared from **FB7** according to the procedure described above.

R_f: 0.21 (Al_2O_3, DCM/methanol=10:1).

^{1}H NMR (400 MHz, CD_3OD-*d4*): δ(ppm)= 8.57 (t, *J* = 13.5 Hz, 1H), 7.55 (d, *J* = 7.5 Hz, 2H), 7.45 (t, *J* = 7.7 Hz, 2H), 7.37-7.29 (m, 4H), 6.50 (d, *J* = 13.4 Hz, 2H), 4.14 (t, *J* = 7.8 Hz, 4H), 1.88-1.80 (m, 4H), 1.78 (s, 12H), 1.57-1.47 (m, 4H), 1.48-1.21 (m, 24H); **^{13}C NMR** (101 MHz, CD_3OD-*d4*): δ(ppm)= 176.03, 152.31, 143.23, 142.26, 130.01, 126.76, 123.51, 112.46, 103.51, 50.67, 45.52, 30.74, 30.13, 29.98, 29.77, 29.64, 29.59, 28.68, 28.33, 27.90; **HRMS-ESI:** $[M]^+$ m/z = 579.4670, calcd. for $[C_{41}H_{59}N_2^+]$: 579.4673.

Preparation of CY-8.

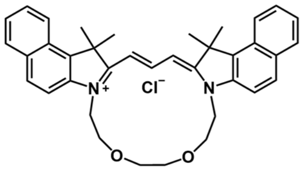

CY-8 (21 mg, 16%) was prepared from **FB8** according to the procedure described above.

R_f: 0.22 (Al_2O_3, DCM/methanol=10:1).

^{1}H NMR (400 MHz, CD_3OD-*d4*): δ(ppm)= 9.43 (s, 1H), 8.22 (d, *J* = 8.7 Hz, 2H), 8.05 (d, *J* = 8.9 Hz, 2H), 8.01 (d, *J* = 8.3 Hz, 2H), 7.68-7.61 (m, 5H), 7.51 (ddd, *J* = 8.2, 7.0, 1.1 Hz, 2H), 6.38 (s, 2H), 4.71 (s, 4H), 4.12 (s, 4H), 3.75 (s, 4H), 1.85 (s, 12H); **^{13}C NMR** (101 MHz, CD_3OD-*d4*): δ(ppm)= 178.23, 151.52, 141.74, 133.59, 133.50, 131.71, 130.96, 129.88, 128.84, 126.37, 123.62, 111.91, 103.39, 72.02, 68.82, 64.31, 53.38, 26.72; **HRMS-ESI:** $[M]^+$ m/z = 543.3005, calcd. for $[C_{37}H_{39}N_2O_2^+]$: 543.3006.

Preparation of CY-9.

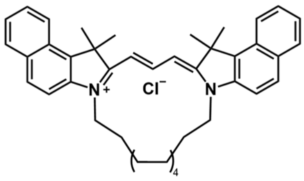

CY-9 (74.2 mg, 44%) was prepared from **FB9** according to the procedure described above.

R_f: 0.25 (Al_2O_3, DCM/methanol=10:1).

^{1}H NMR (400 MHz, CD_3OD-*d4*): δ(ppm)= 8.78 (t, *J* = 13.5 Hz, 1H), 8.29 (d, *J* = 8.7 Hz, 2H), 8.05 (d, *J* = 8.8 Hz, 2H), 8.01 (d, *J* = 7.3 Hz, 2H), 7.68 (ddd, *J* = 7.0, 6.9, 1.4 Hz, 2H), 7.63 (d, *J* = 8.9 Hz, 2H), 7.52 (ddd, *J* = 7.0, 6.8, 1.0 Hz, 2H), 6.42 (d, *J* = 13.6 Hz, 2H), 4.28 (t, *J* = 7.5 Hz, 4H), 2.10 (s, 12H), 1.99-1.90 (m, 4H), 1.57-1.52 (m, 8H), 1.47-1.39 (m, 8H); **^{13}C NMR** (101 MHz, CD_3OD-*d4*): δ(ppm)= 176.18, 148.91, 139.03, 133.57, 132.18, 130.51, 129.70, 127.80, 127.46, 124.92, 121.84, 110.79, 101.48, 51.04, 43.21, 28.56, 28.11, 27.58, 26.56, 26.25, 25.12; **HRMS-ESI:** $[M]^+$ m/z = 595.4047, calcd. for $[C_{37}H_{39}N_2O_2^+]$: 595.4039.

3. Supplementary table and figures

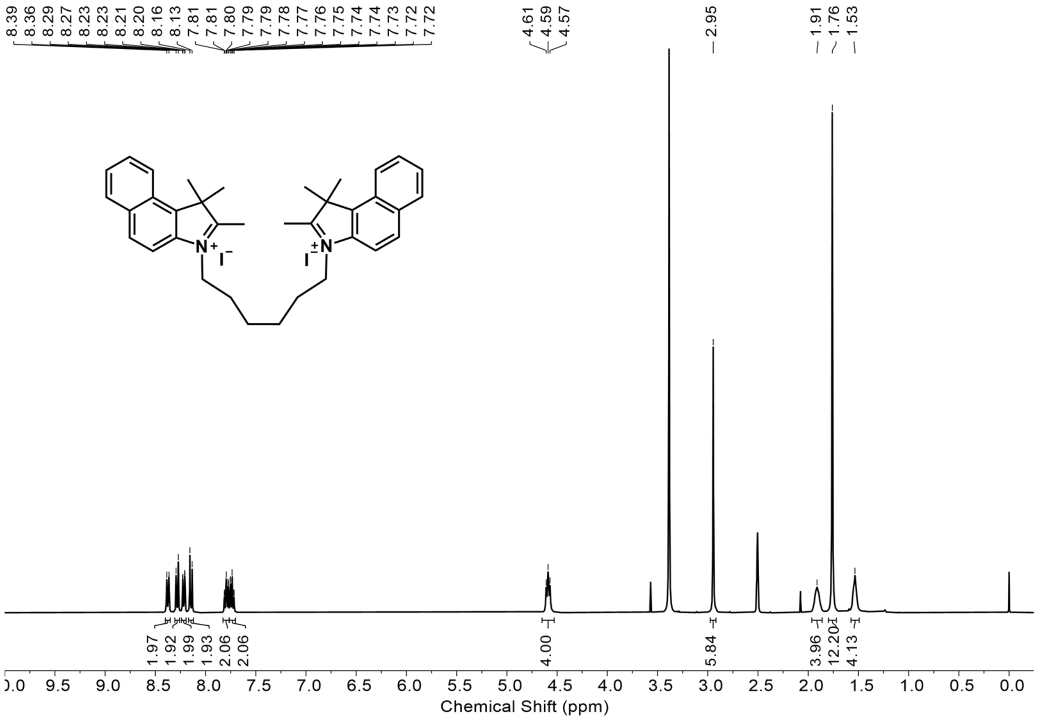

Figure S2. ^{1}H NMR spectrum of **Indo1** in DMSO-*d6* (400 MHz).

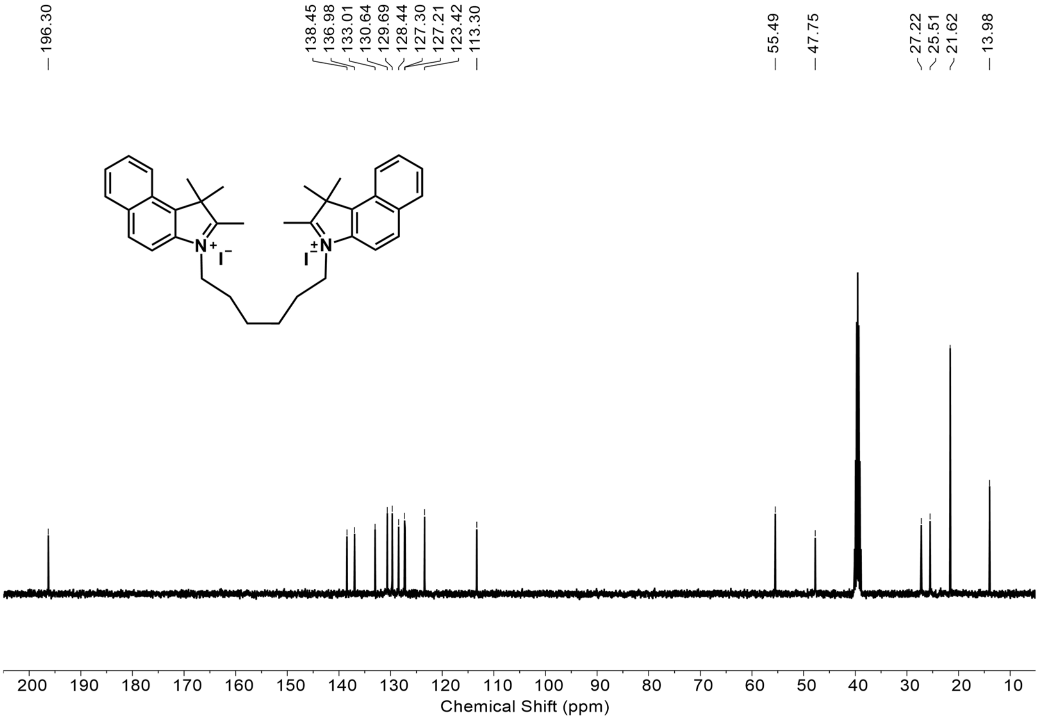

Figure S3. ^{13}C NMR spectrum of **Indo1** in DMSO-*d6* (101 MHz).

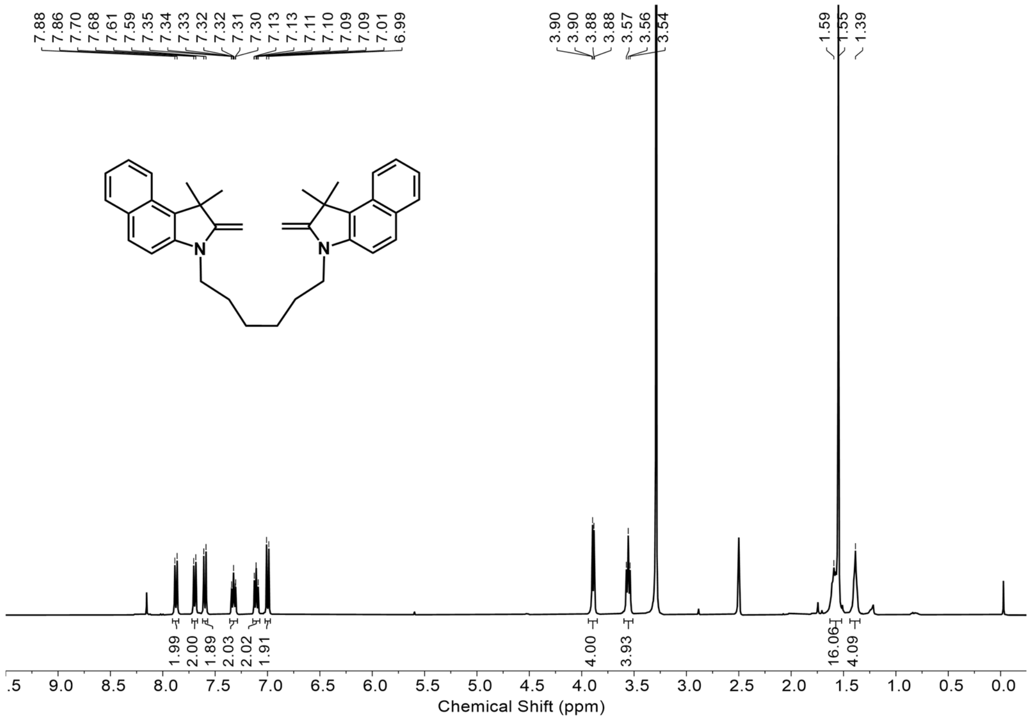

Figure S4. ^{1}H NMR spectrum of **FBI** in DMSO-*d6*-$CDCl_3$ (400 MHz).

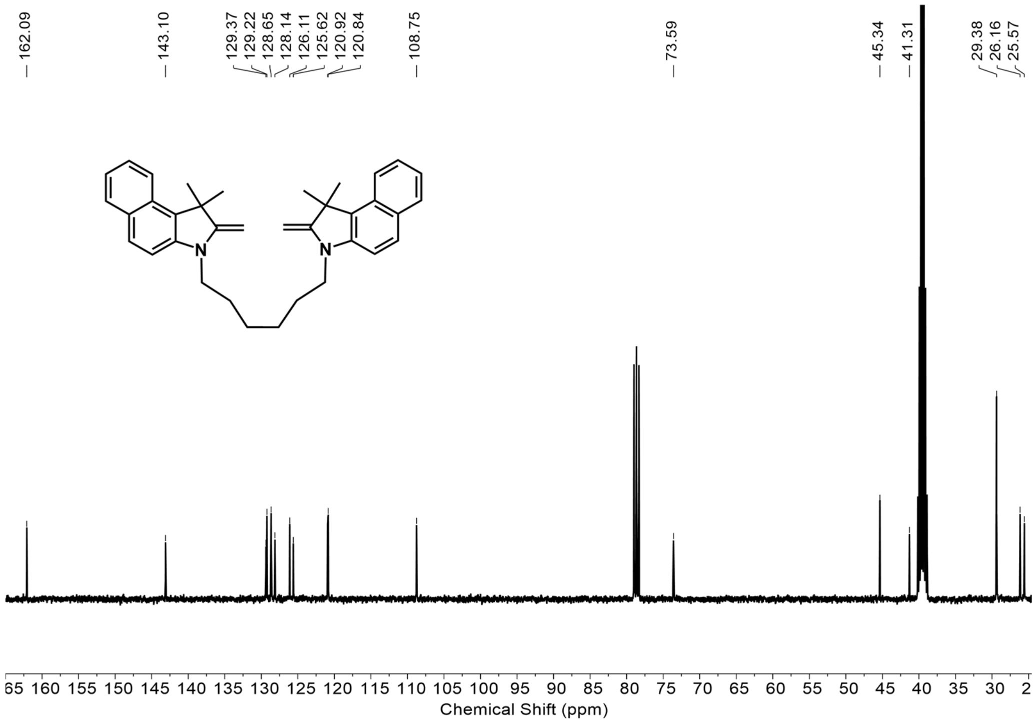

Figure S5. ^{13}C NMR spectrum of **FBI** in DMSO-*d6*-$CDCl_3$ (101 MHz).

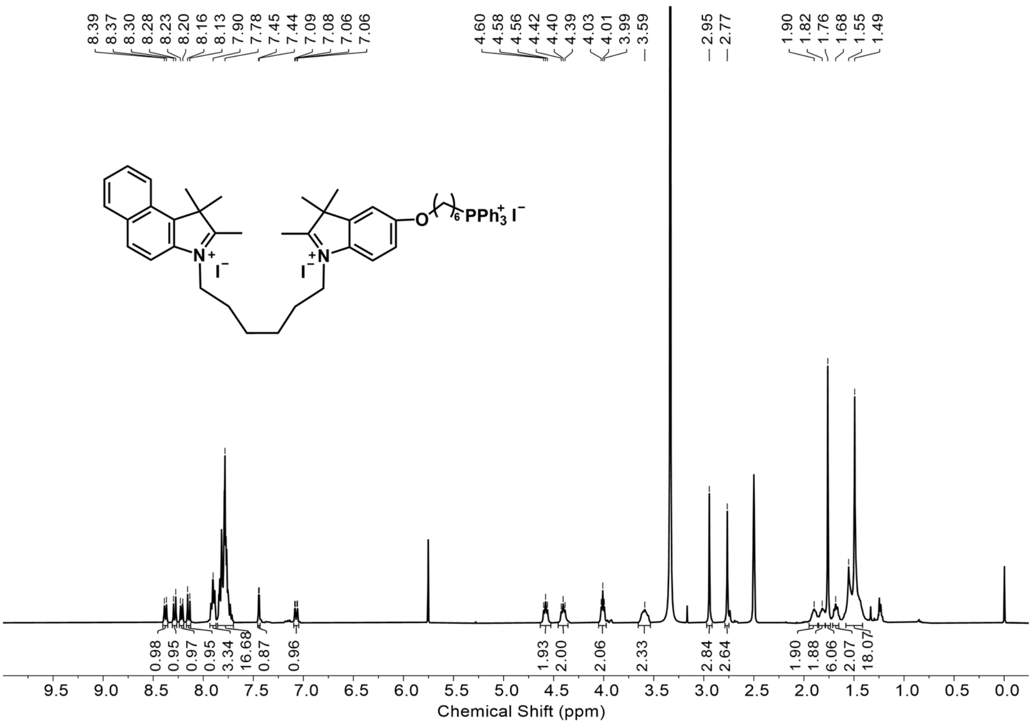

Figure S6. ^{1}H NMR spectrum of **Indo2** in DMSO-*d6* (400 MHz).

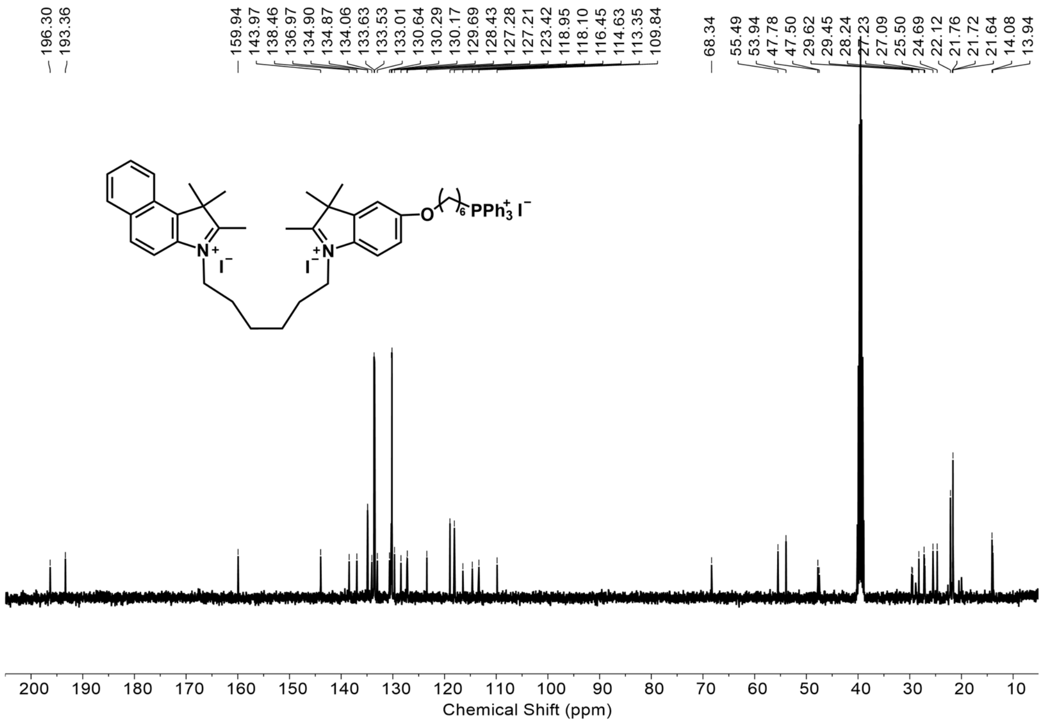

Figure S7. ^{13}C NMR spectrum of **Indo2** in DMSO-*d6* (101 MHz).

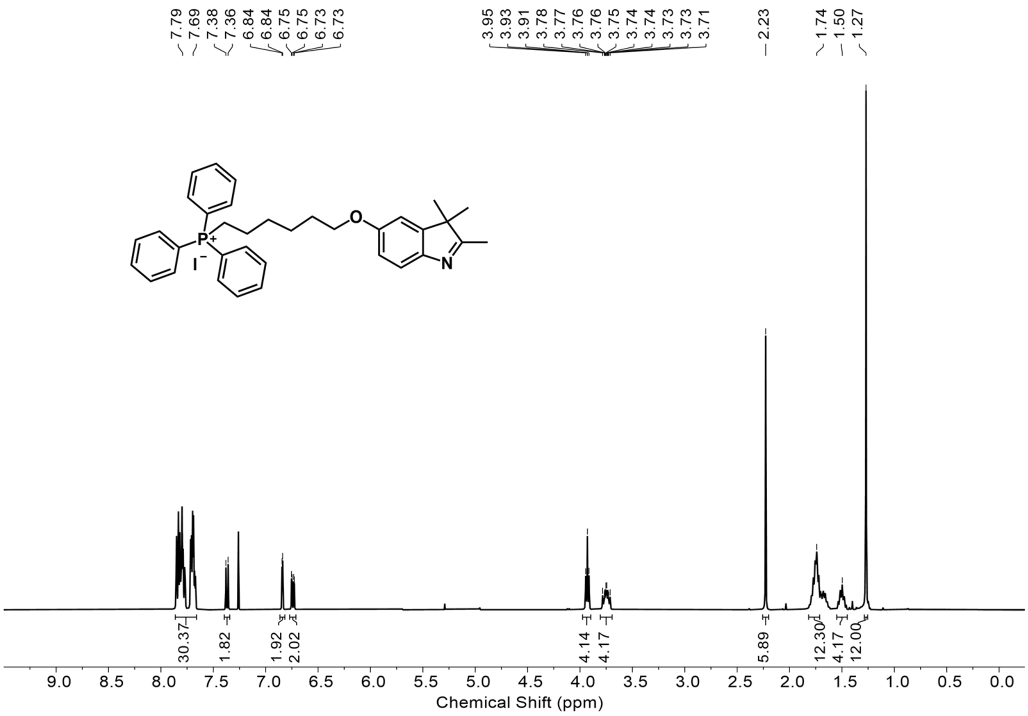

Figure S8. ^{1}H NMR spectrum of **TPP-Indo** in $CDCl_3$ (400 MHz).

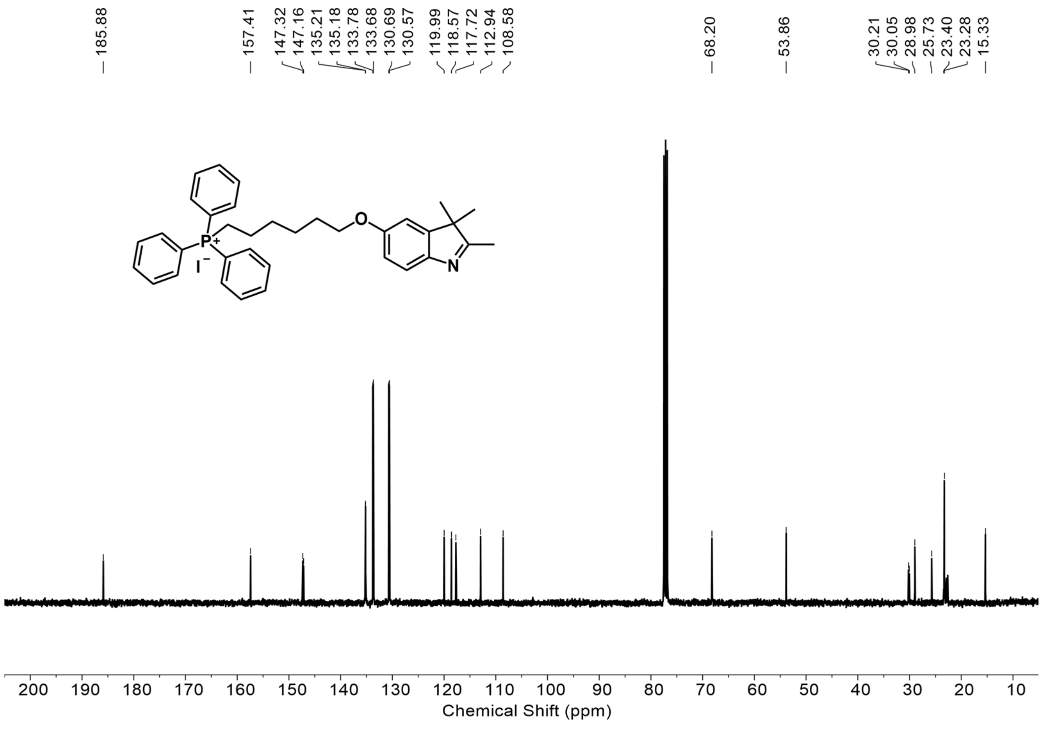

Figure S9. ^{13}C NMR spectrum of **TPP-Indo** in $CDCl_3$ (101 MHz).

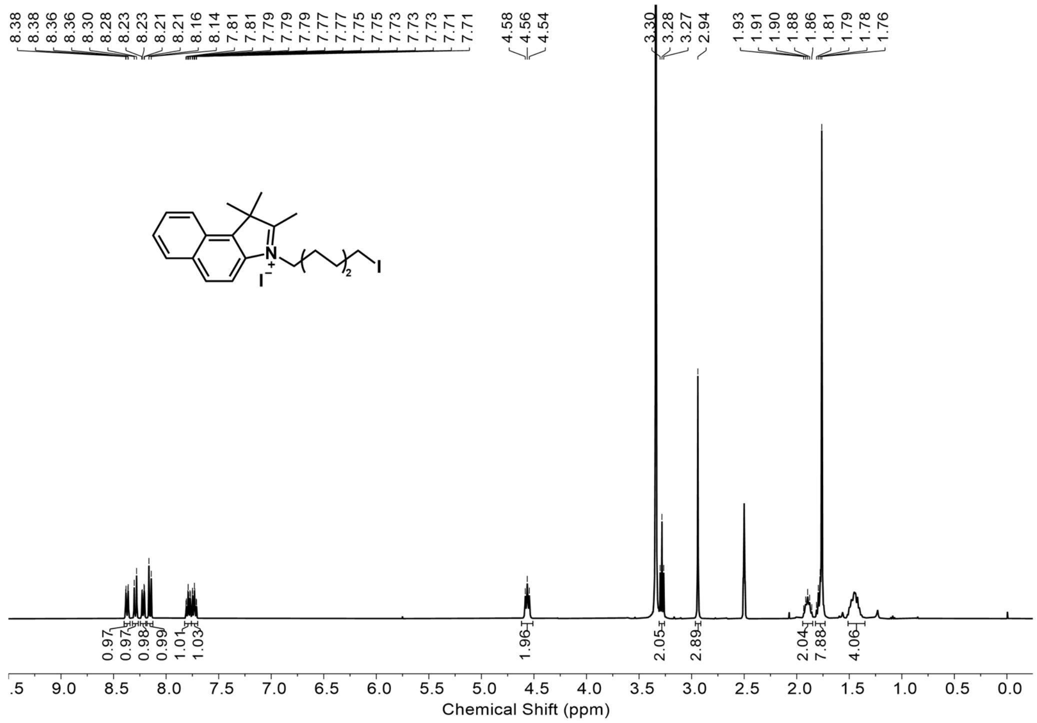

Figure S10. ^{1}H NMR spectrum of **Indo-C6** in DMSO-*d6* (400 MHz).

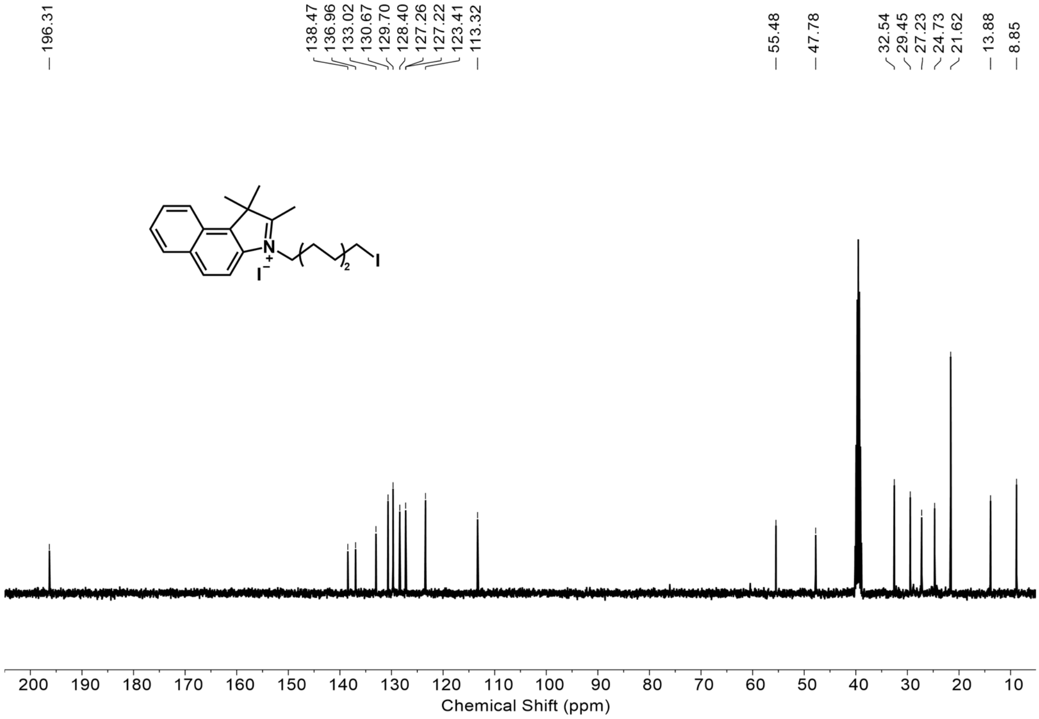

Figure S11. ^{13}C NMR spectrum of **Indo-C6** in DMSO-*d6* (101 MHz).

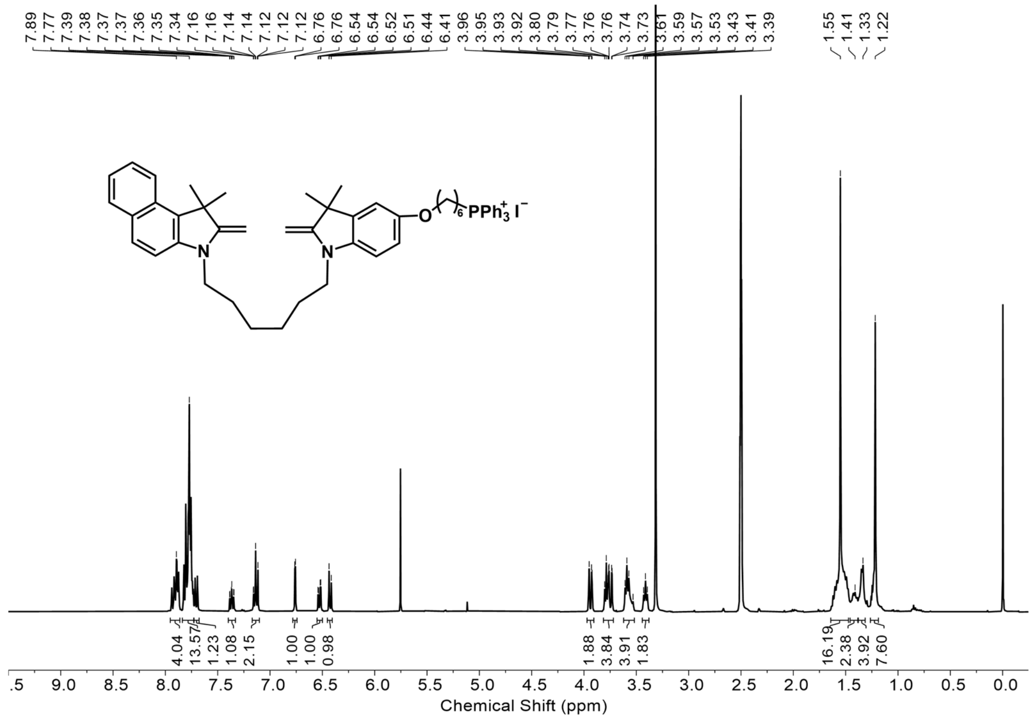

Figure S12. ^{1}H NMR spectrum of **FB2** in DMSO-*d6* (400 MHz).

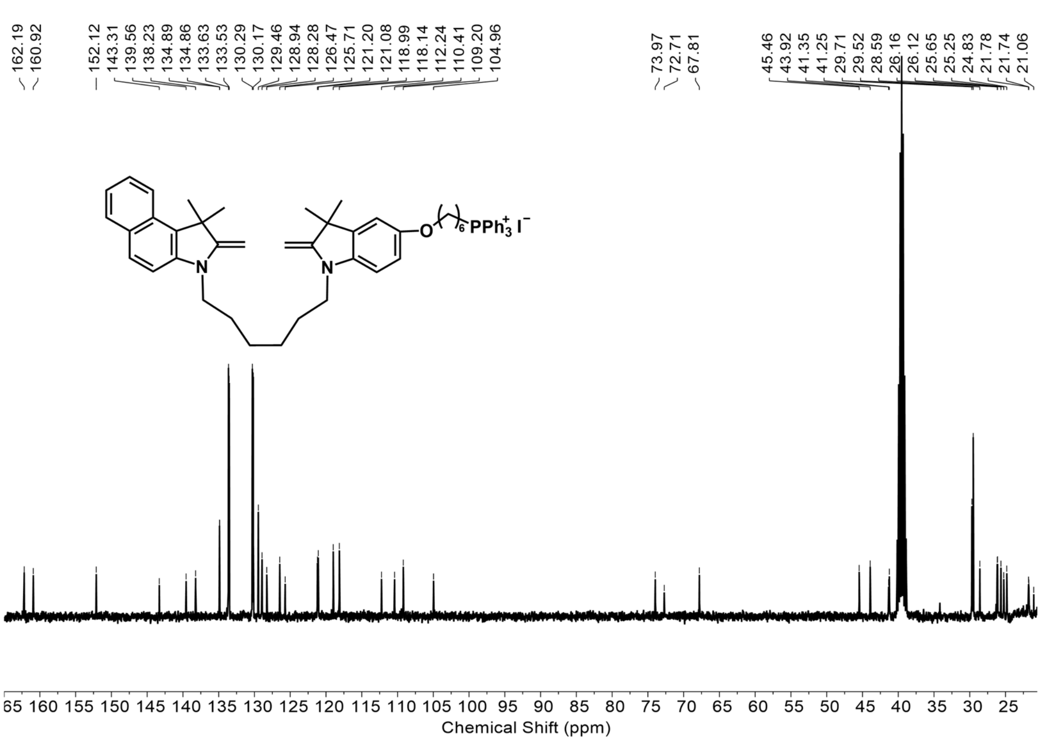

Figure S13. ^{13}C NMR spectrum of **FB2** in DMSO-*d6* (101 MHz).

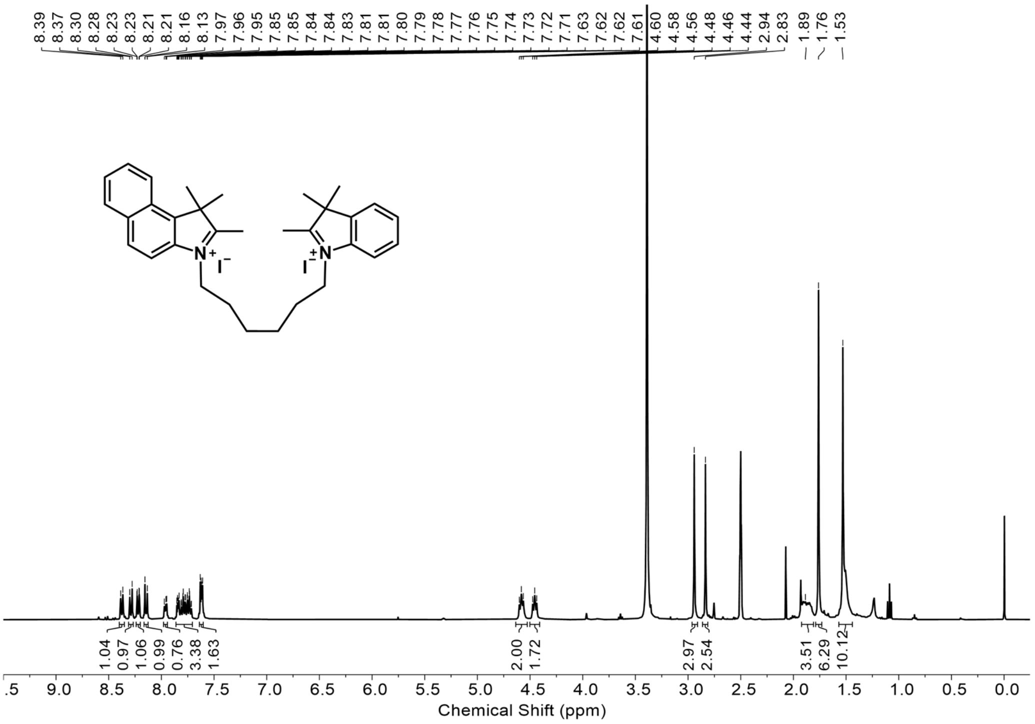

Figure S14. ^{1}H NMR spectrum of **Indo3** in DMSO-*d6* (400 MHz).

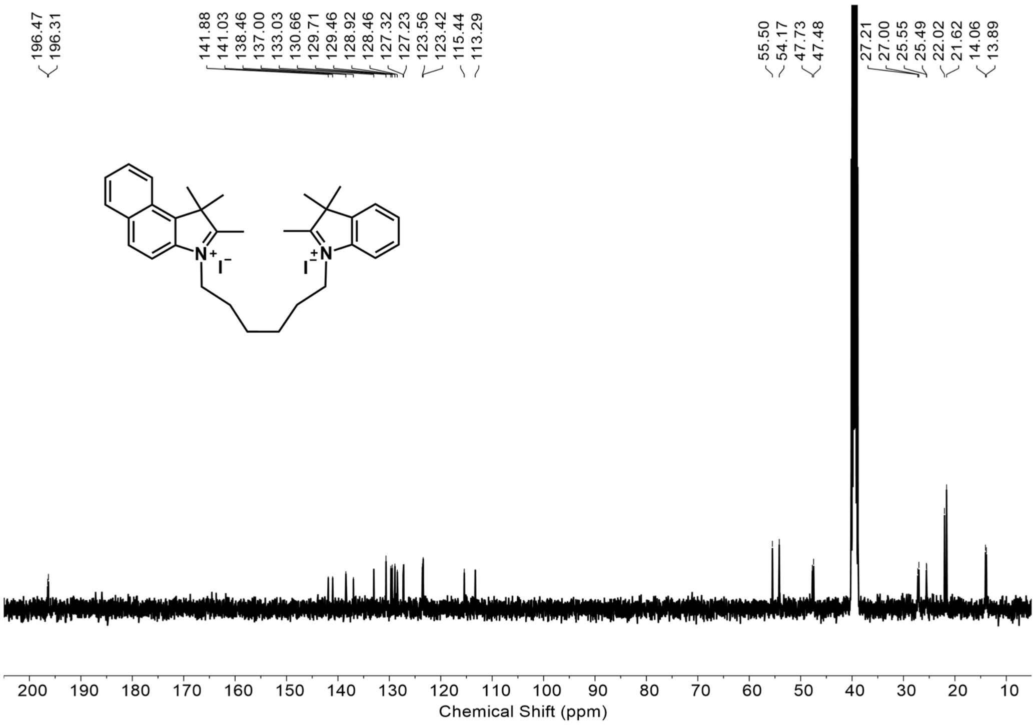

Figure S15. ^{13}C NMR spectrum of **Indo3** in DMSO-*d6* (101 MHz).

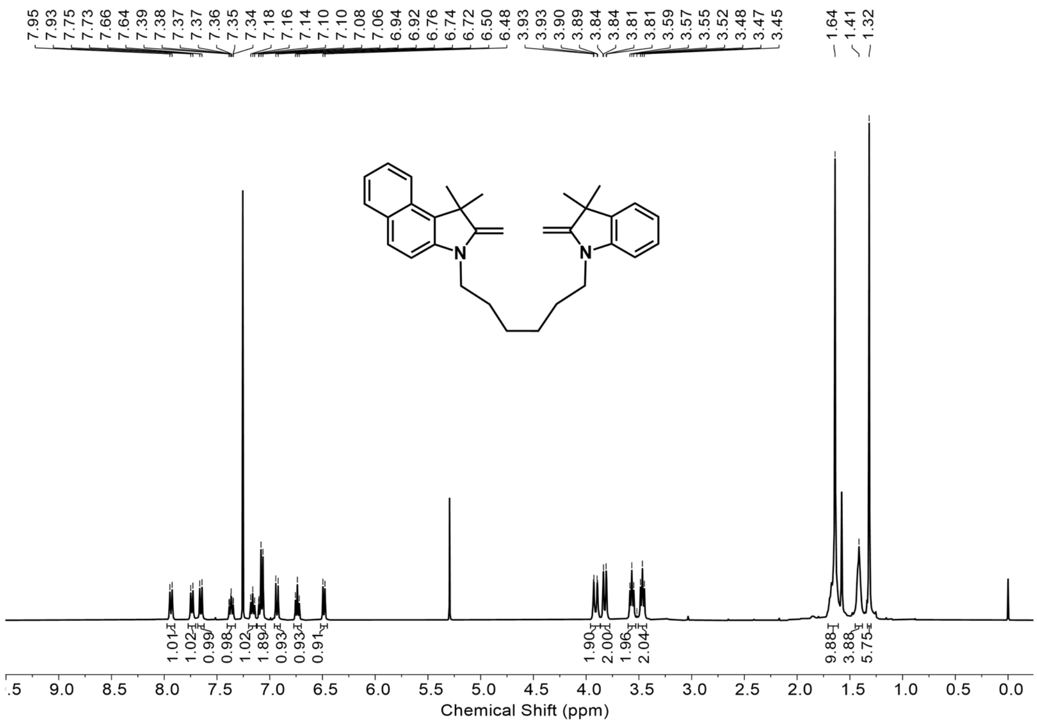

Figure S16. ^{1}H NMR spectrum of **FB3** in $CDCl_3$ (400 MHz).

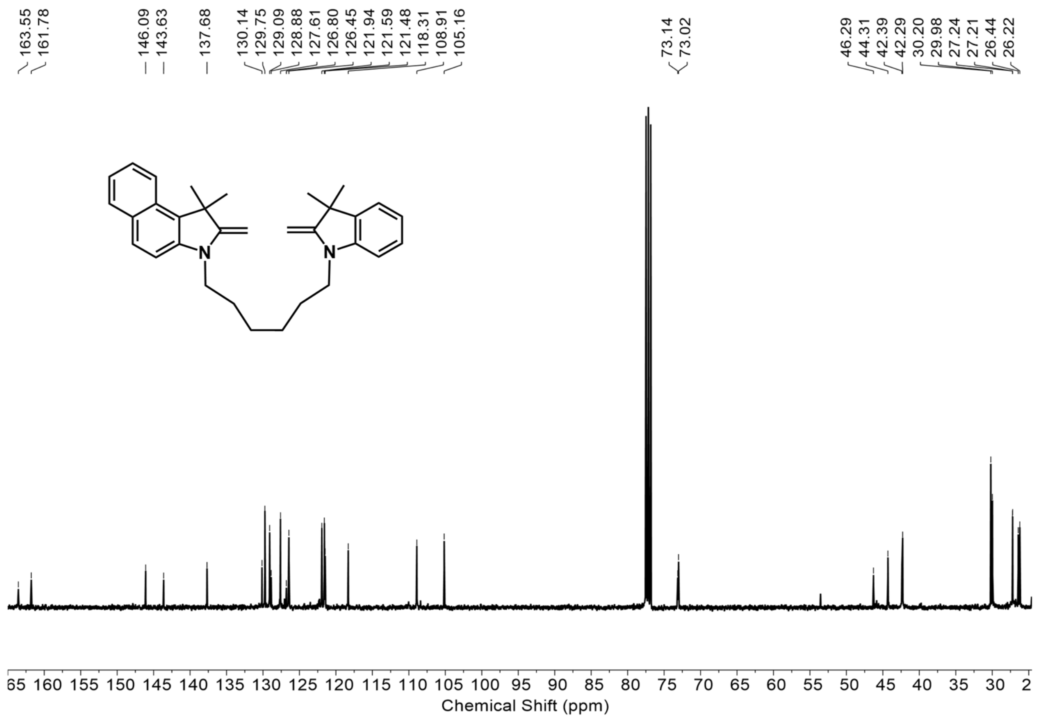

Figure S17. ^{13}C NMR spectrum of **FB3** in $CDCl_3$ (101 MHz).

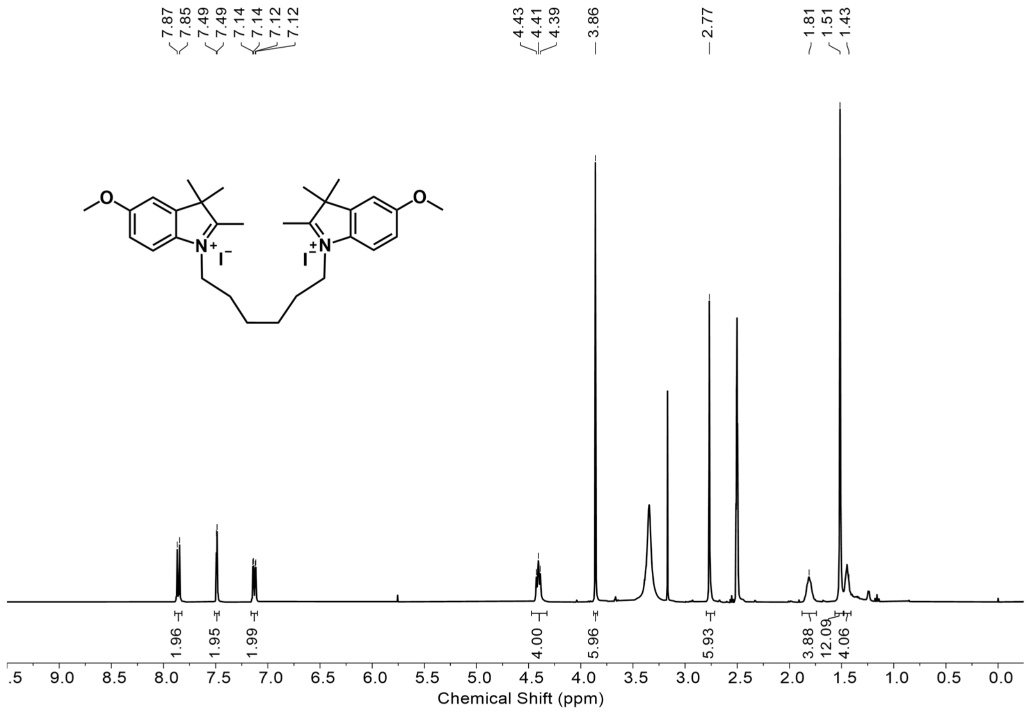

Figure S18. ^{1}H NMR spectrum of **Indo4** in DMSO-*d6* (400 MHz).

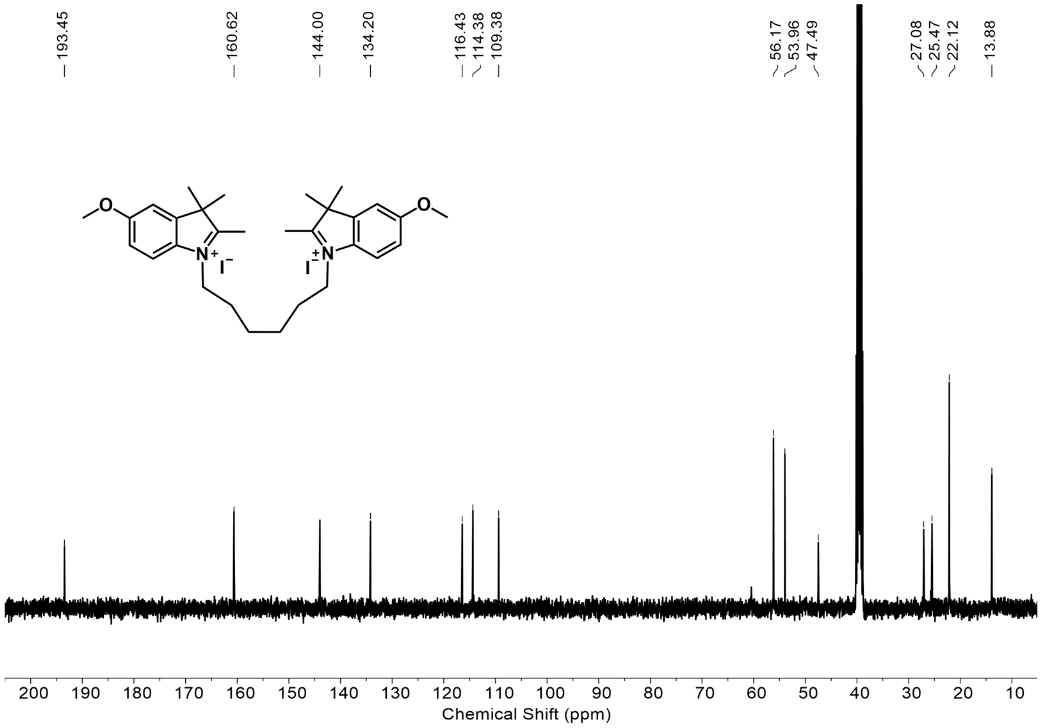

Figure S19. ^{13}C NMR spectrum of **Indo4** in DMSO-*d6* (101 MHz).

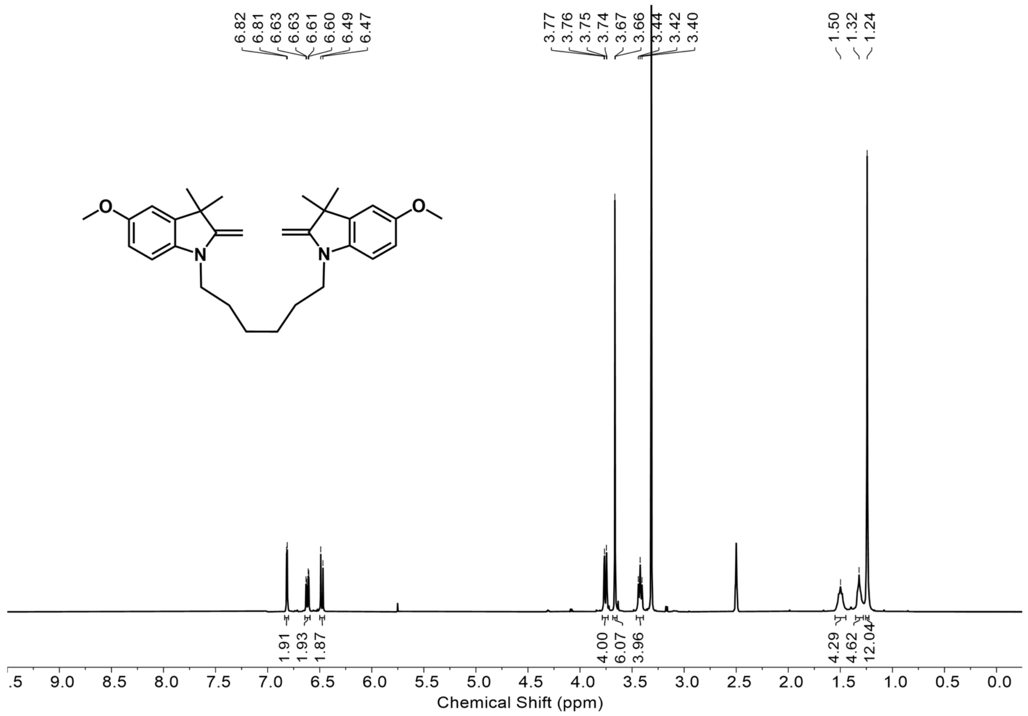

Figure S20. ^{1}H NMR spectrum of **FB4** in DMSO-*d6* (400 MHz).

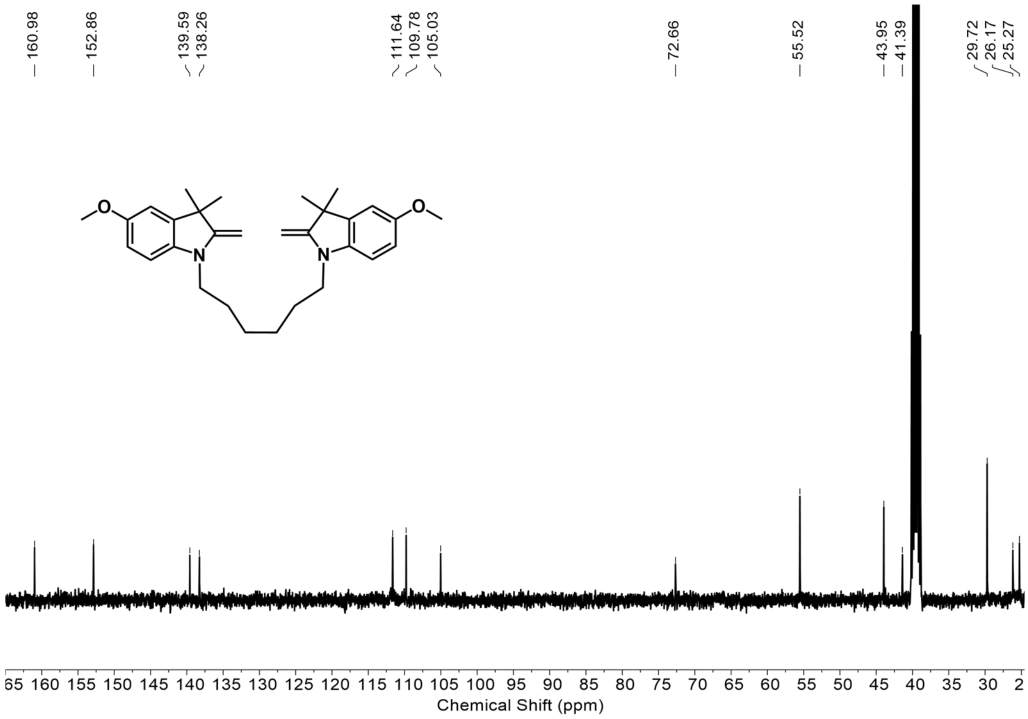

Figure S21. ^{13}C NMR spectrum of **FB4** in DMSO-*d6* (101 MHz).

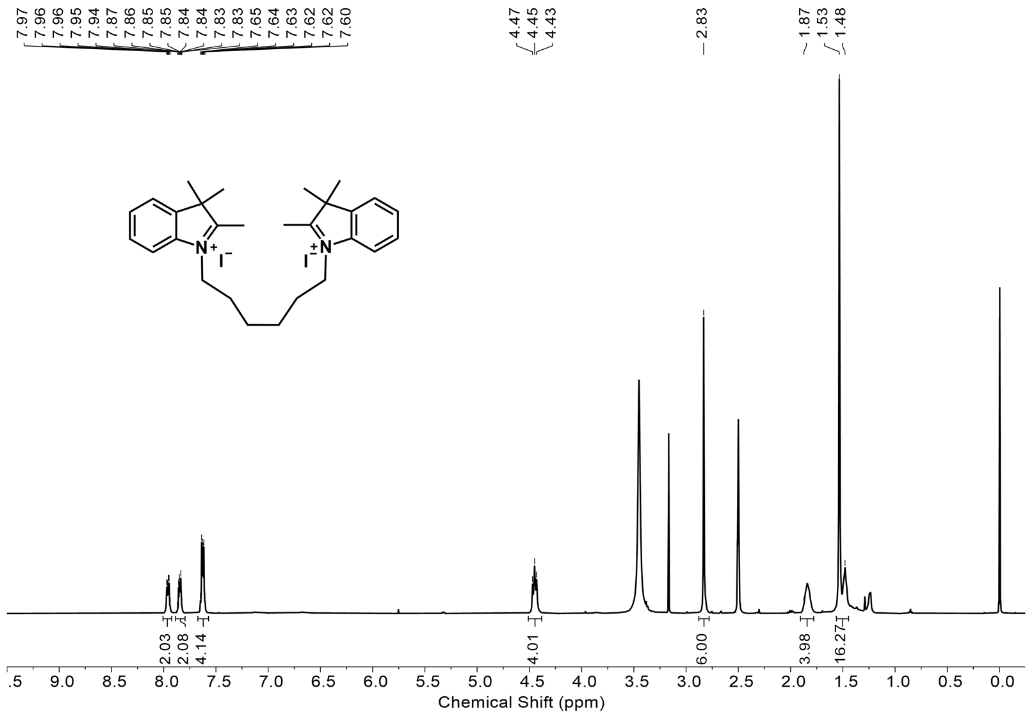

Figure S22. ^{1}H NMR spectrum of **Indo5** in DMSO-*d6* (400 MHz).

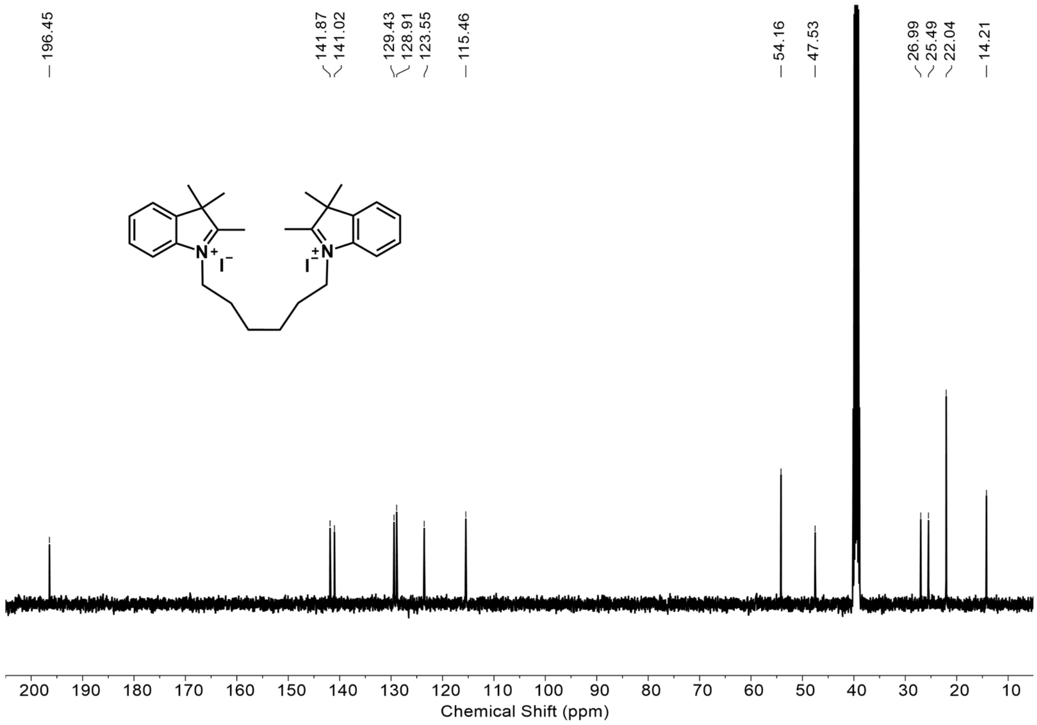

Figure S23. ^{13}C NMR spectrum of **Indo5** in DMSO-*d6* (101 MHz).

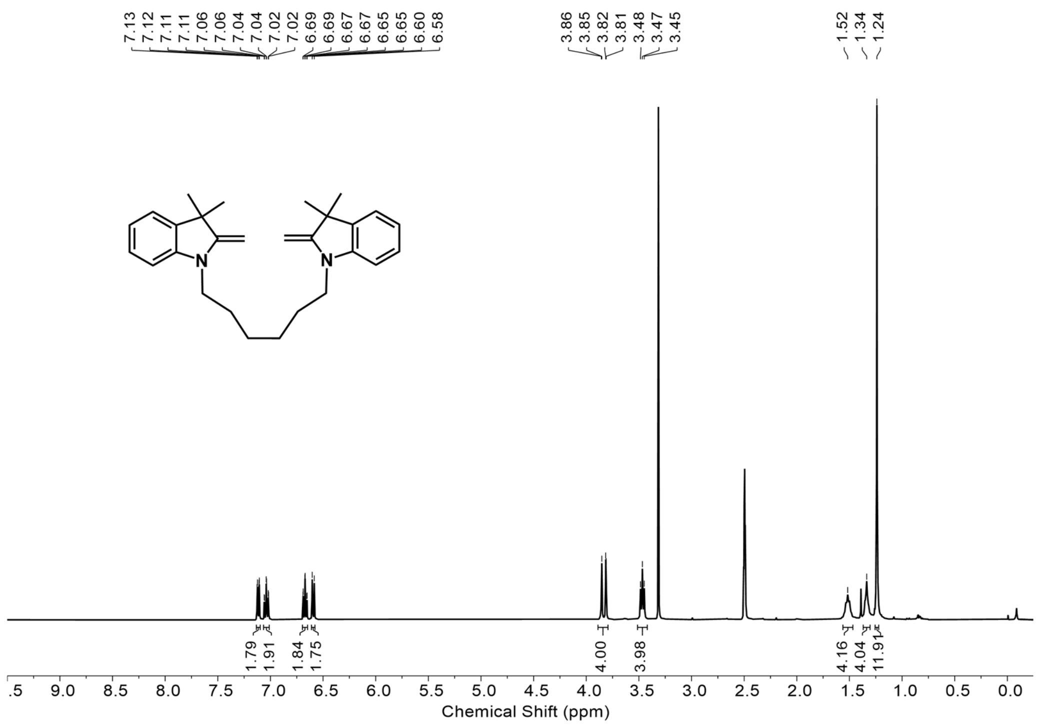

Figure S24. ^{1}H NMR spectrum of **FB5** in DMSO-*d6* (400 MHz).

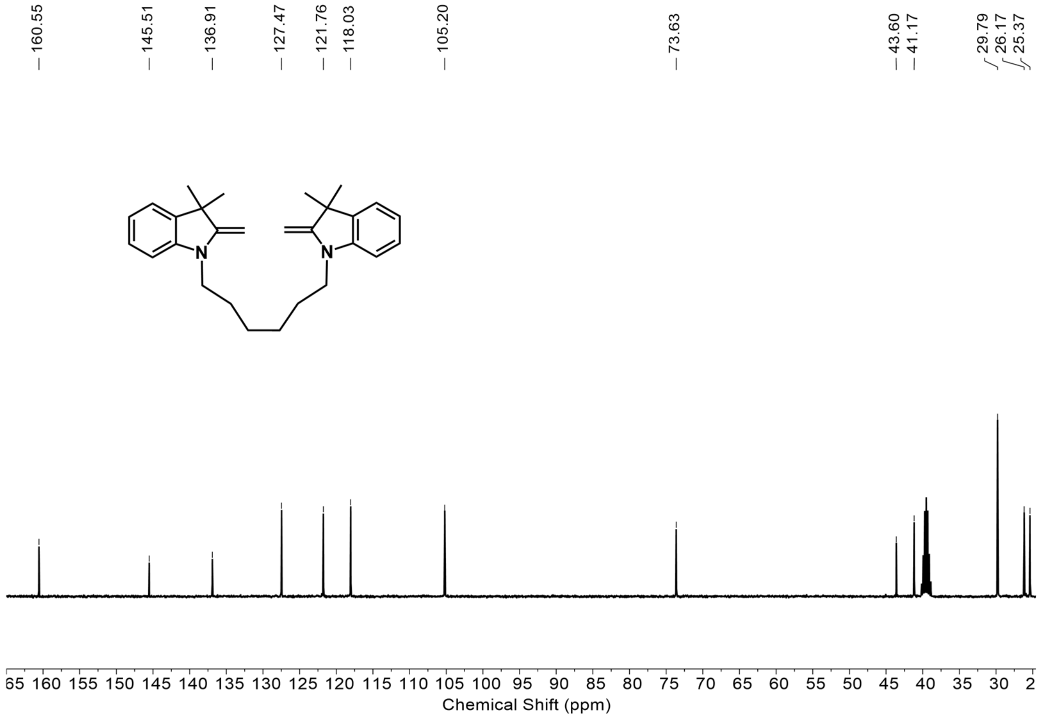

Figure S25. ^{13}C NMR spectrum of **FB5** in DMSO-*d6* (101 MHz).

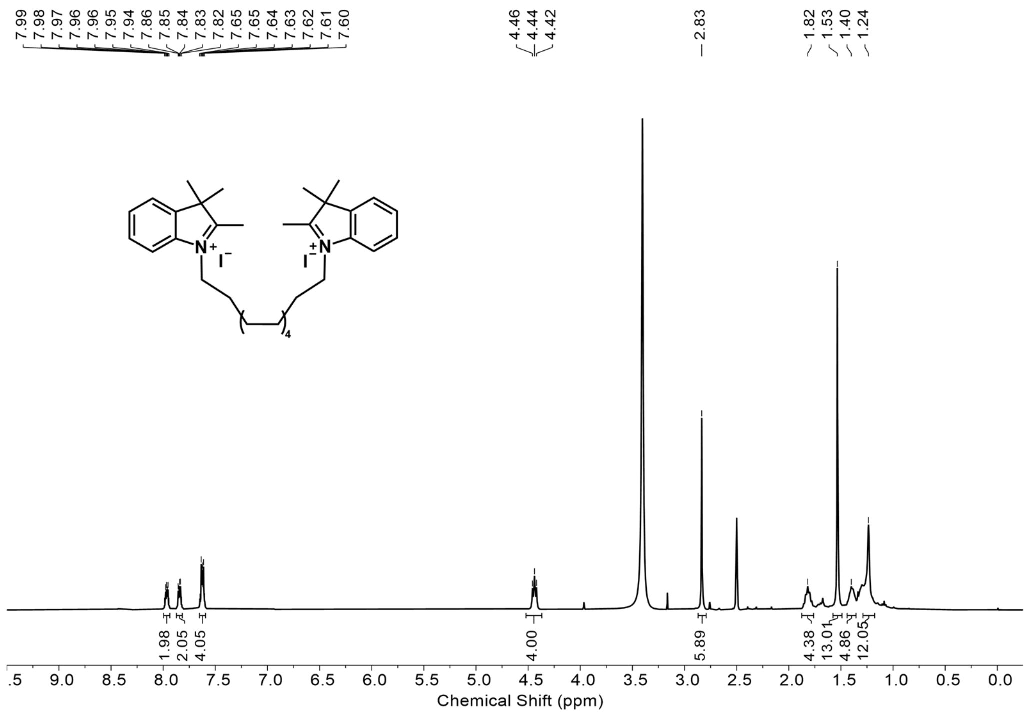

Figure S26. ^{1}H NMR spectrum of **Indo6** in DMSO-*d6* (400 MHz).

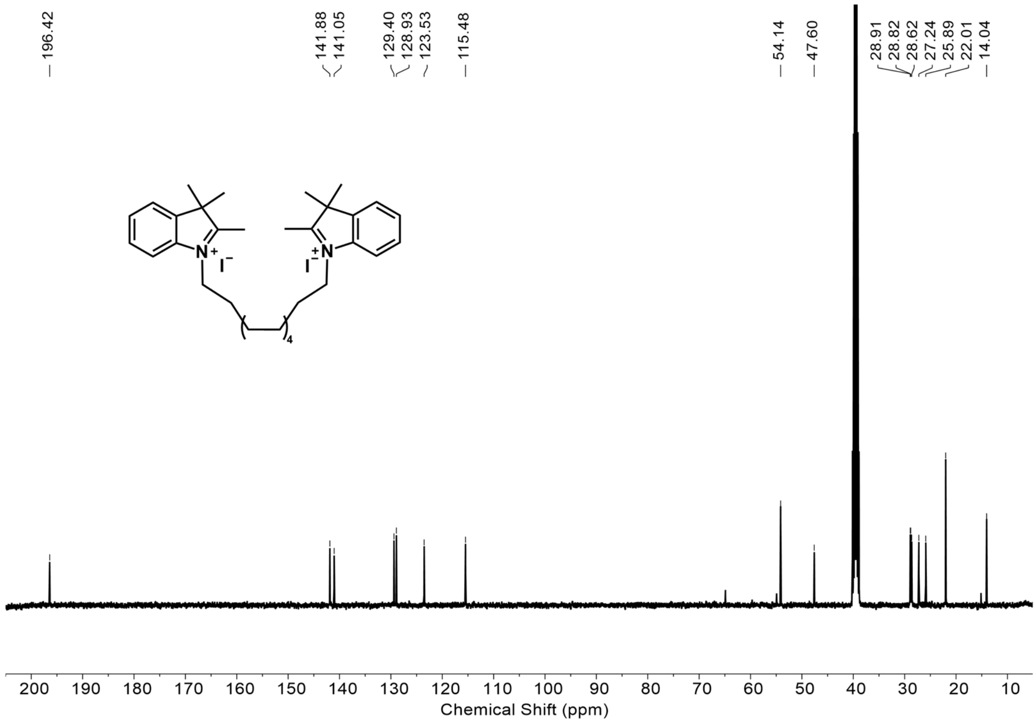

Figure S27. ^{13}C NMR spectrum of **Indo6** in DMSO-*d6* (101 MHz).

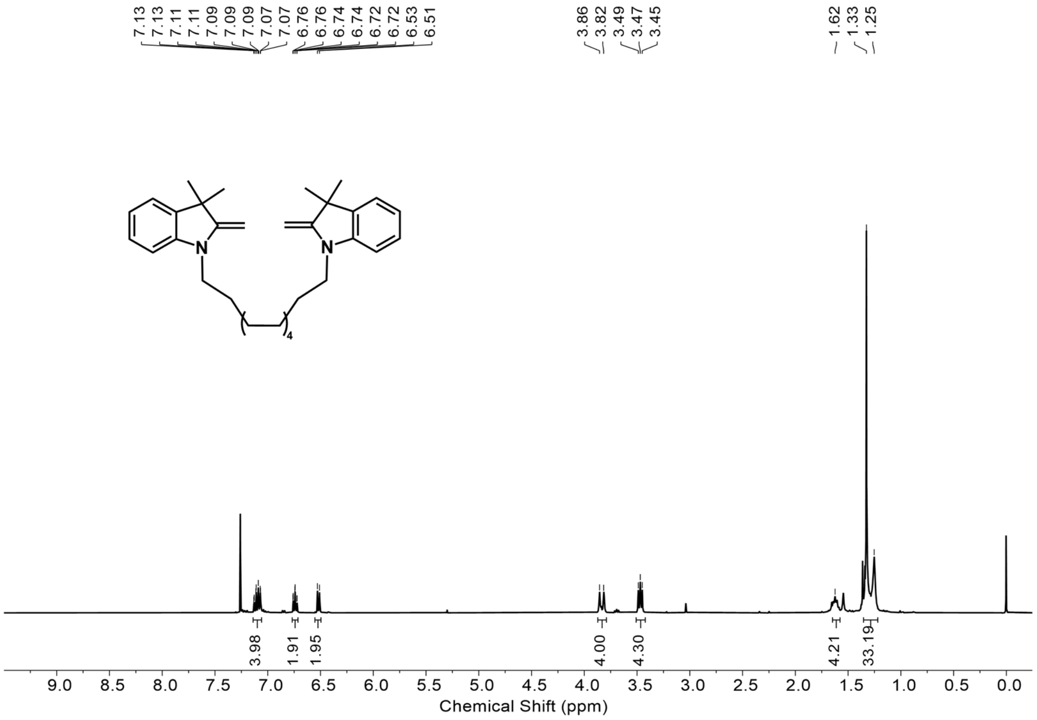

Figure S28. ^1H NMR spectrum of **FB6** in $CDCl_3$ (400 MHz).

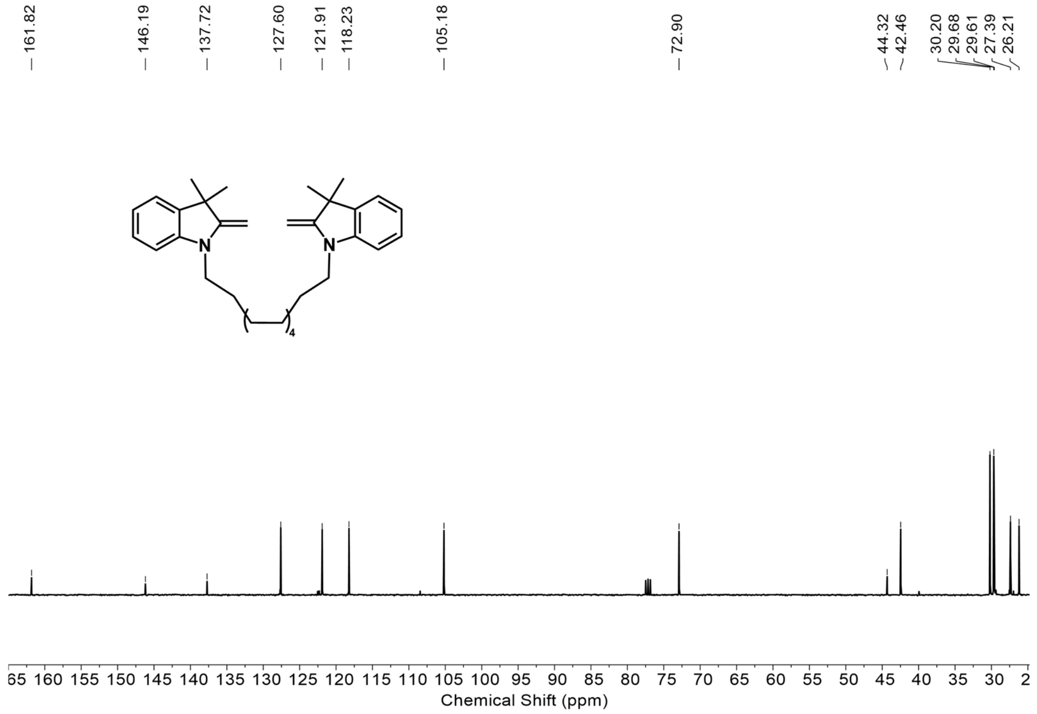

Figure S29. ^{13}C NMR spectrum of **FB6** in $CDCl_3$ (101 MHz).

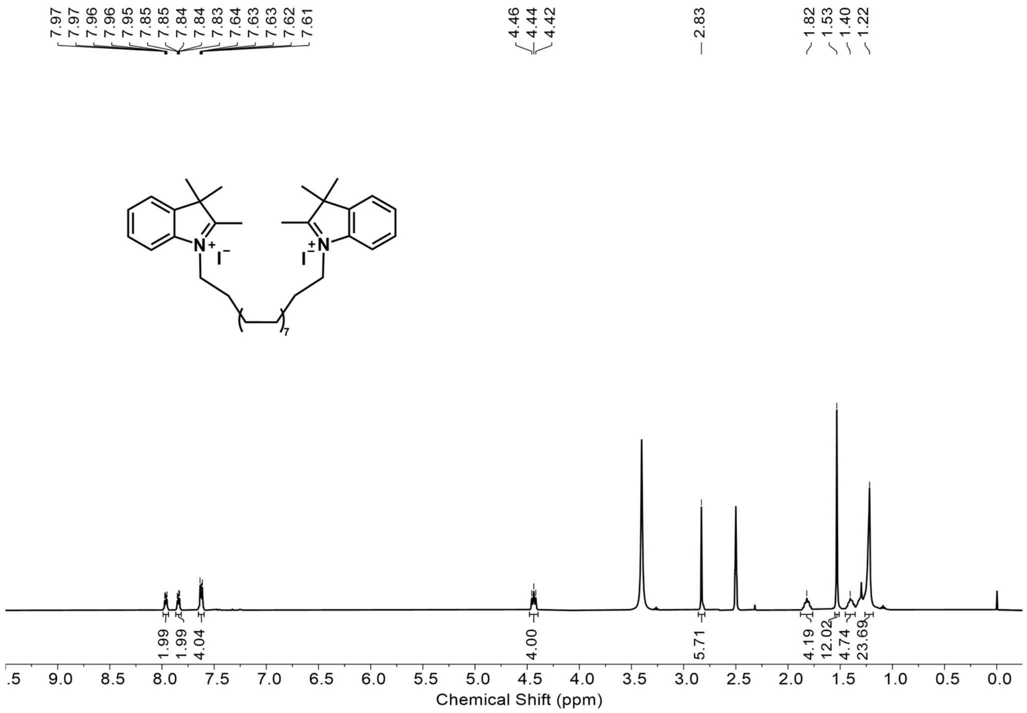

Figure S30. ^{1}H NMR spectrum of **Indo7** in DMSO-*d6* (400 MHz).

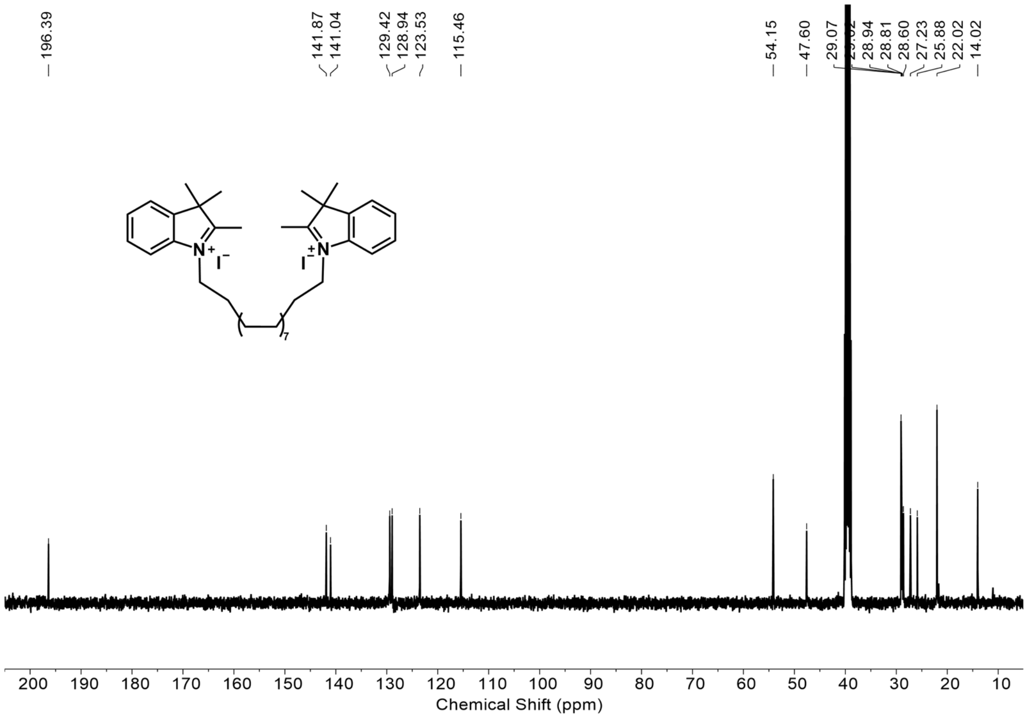

Figure S31. ^{13}C NMR spectrum of **Indo7** in DMSO-*d6* (101 MHz).

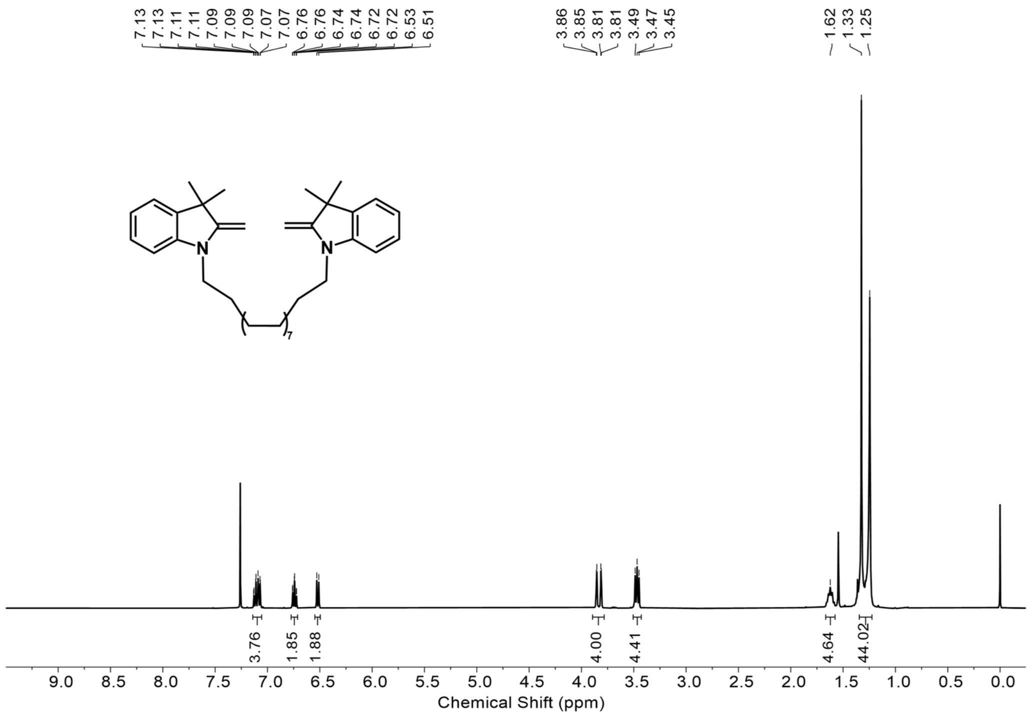

Figure S32. ^{1}H NMR spectrum of **FB7** in $CDCl_3$ (400 MHz).

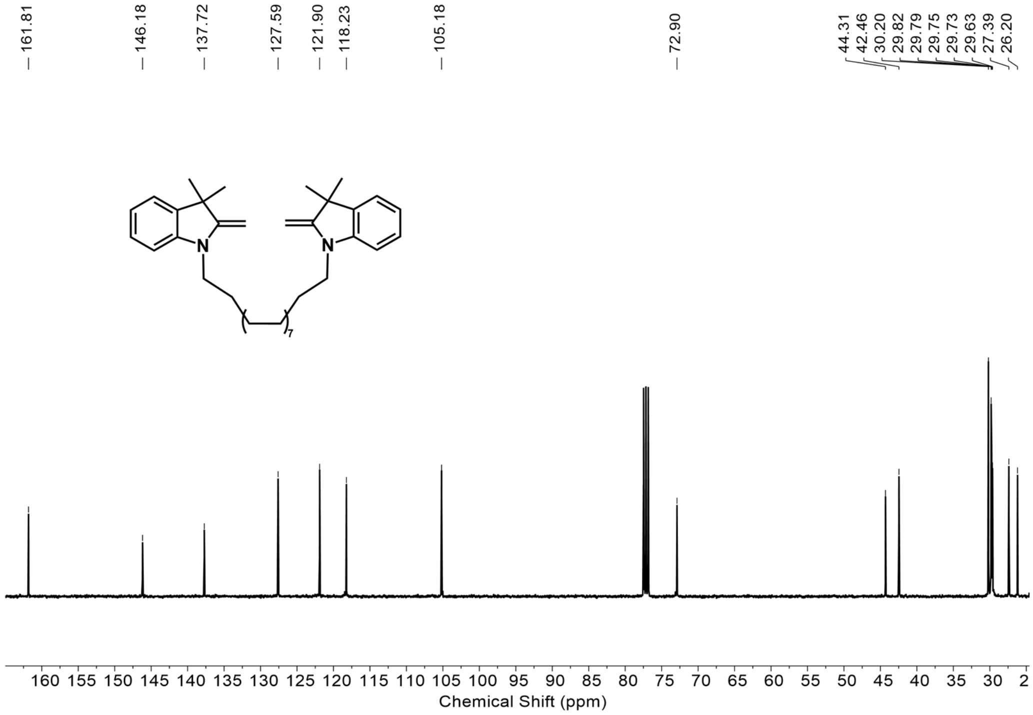

Figure S33. ^{13}C NMR spectrum of **FB7** in $CDCl_3$ (101 MHz).

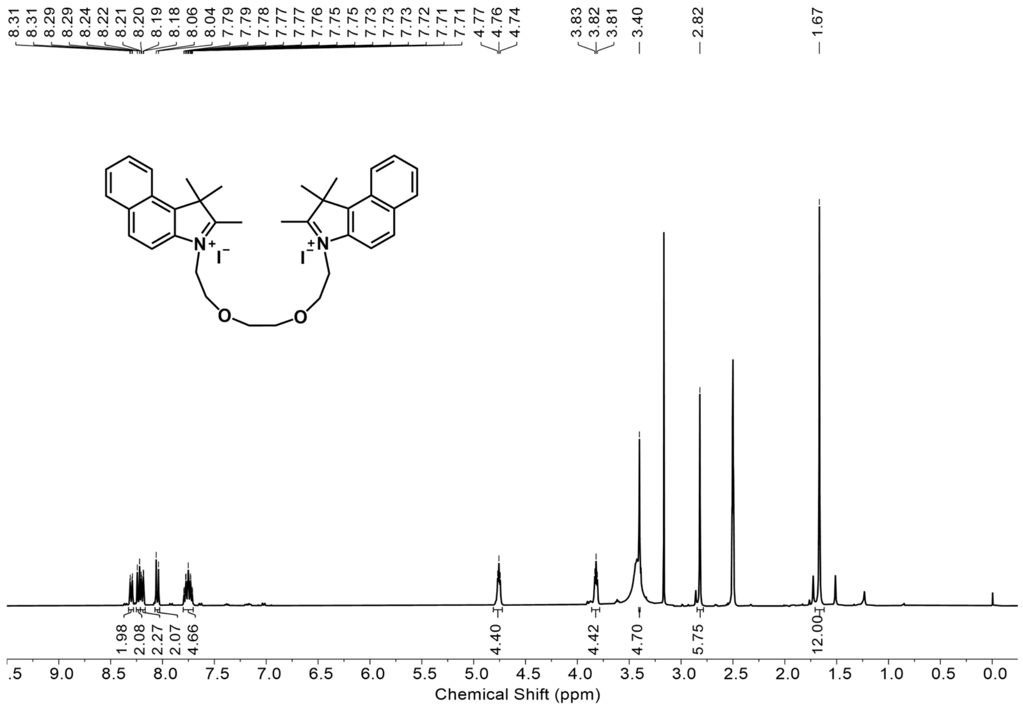

Figure S34. ^{1}H NMR spectrum of **Indo8** in DMSO-*d6* (400 MHz).

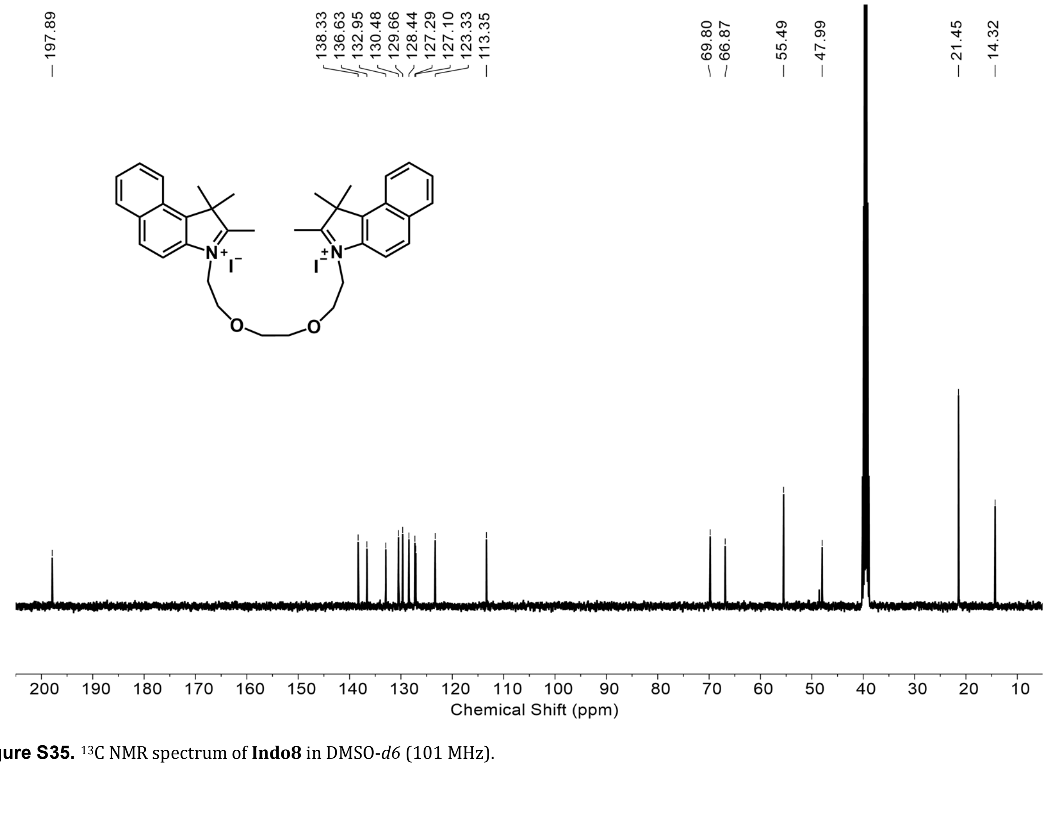

Figure S35. ^{13}C NMR spectrum of **Indo8** in DMSO-*d6* (101 MHz).

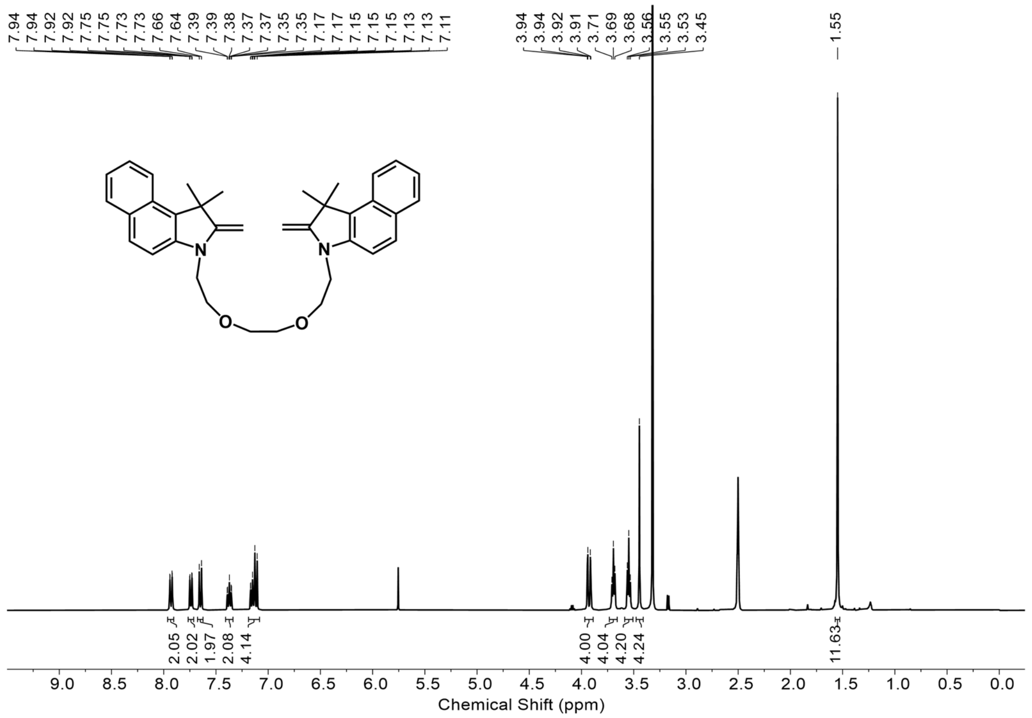

Figure S36. ^{1}H NMR spectrum of **FB8** in DMSO-*d6* (400 MHz).

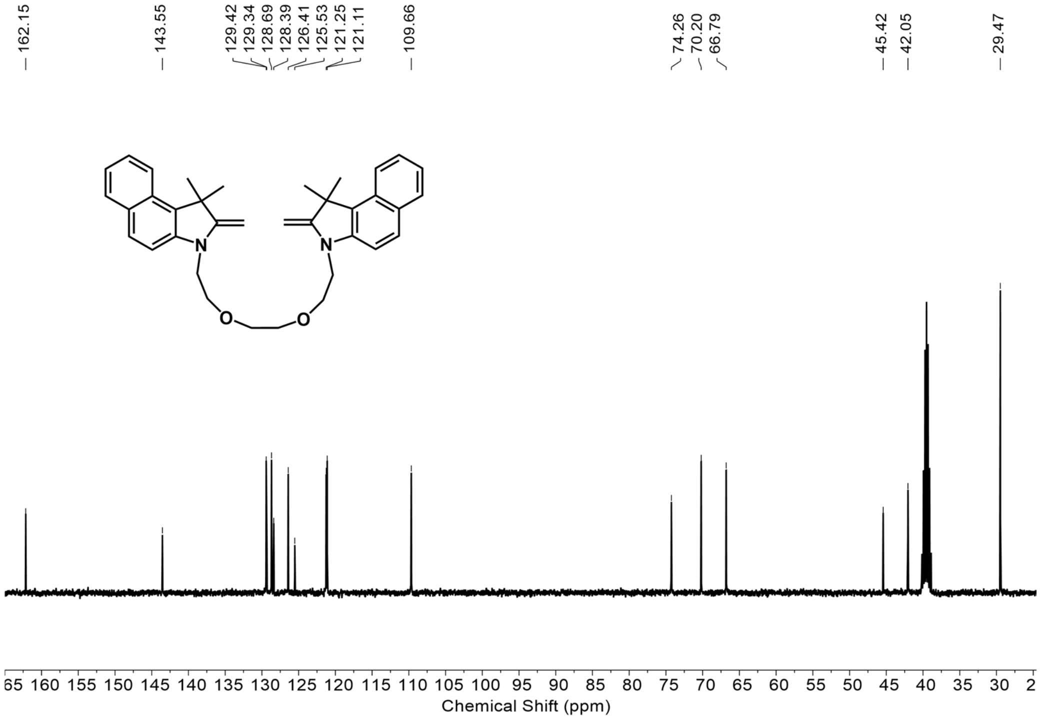

Figure S37. ^{13}C NMR spectrum of **FB8** in DMSO-*d6* (101 MHz).

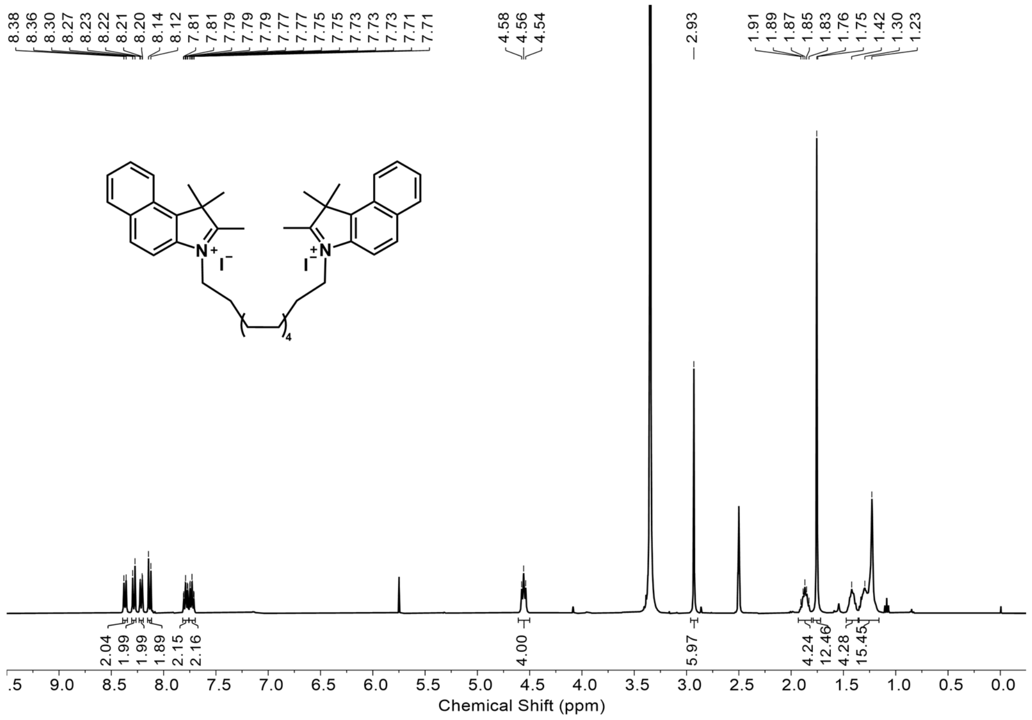

Figure S38. ^{1}H NMR spectrum of **Indo9** in DMSO-*d6* (400 MHz).

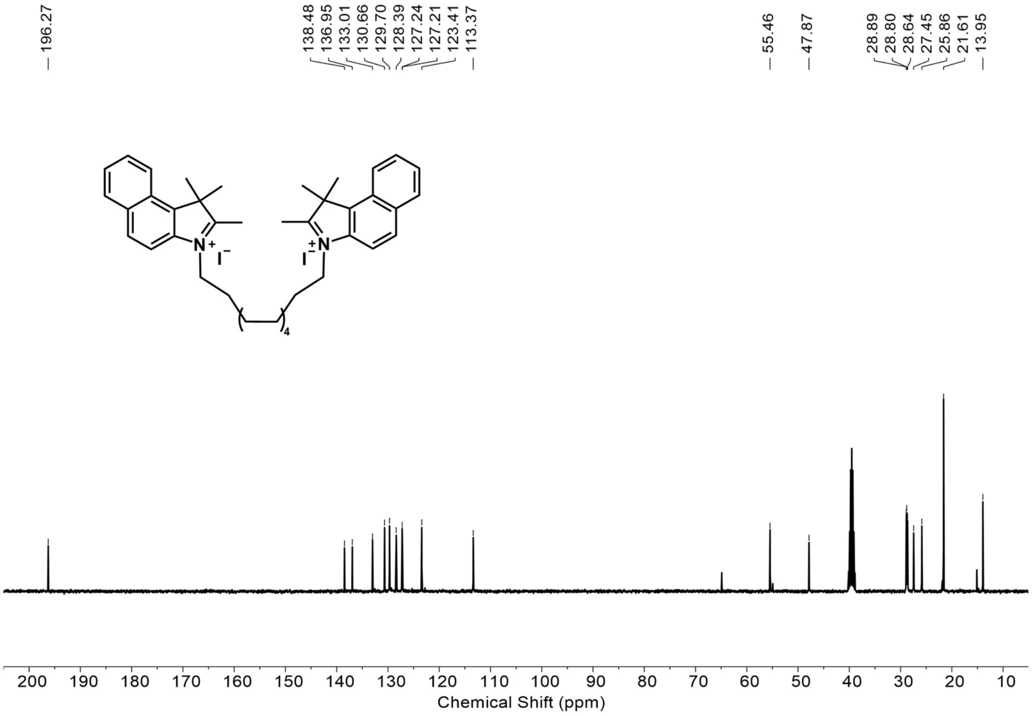

Figure S39. ^{13}C NMR spectrum of **Indo9** in DMSO-*d6* (101 MHz).

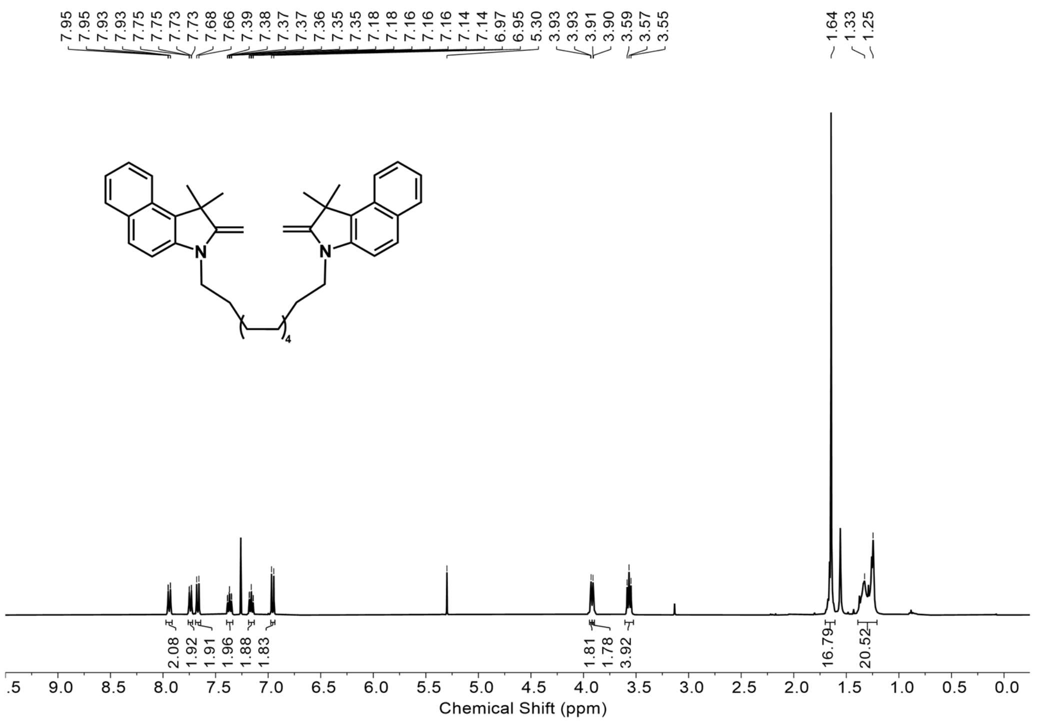

Figure S40. ^{1}H NMR spectrum of **FB9** in $CDCl_3$ (400 MHz).

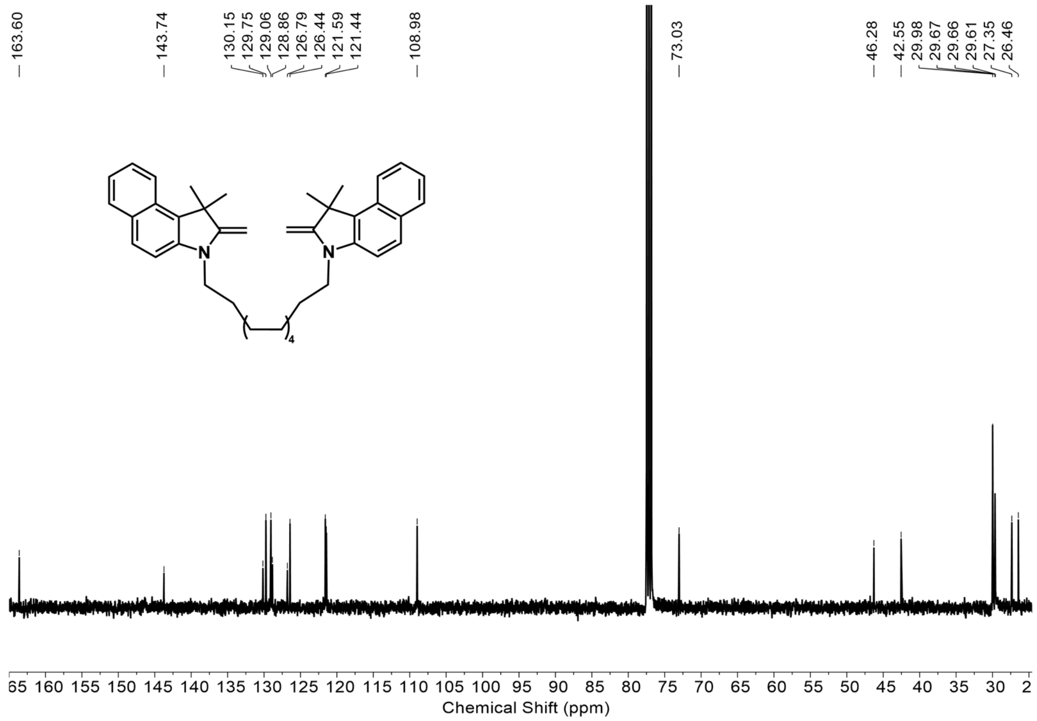

Figure S41. ^{13}C NMR spectrum of **FB9** in $CDCl_3$ (101 MHz).

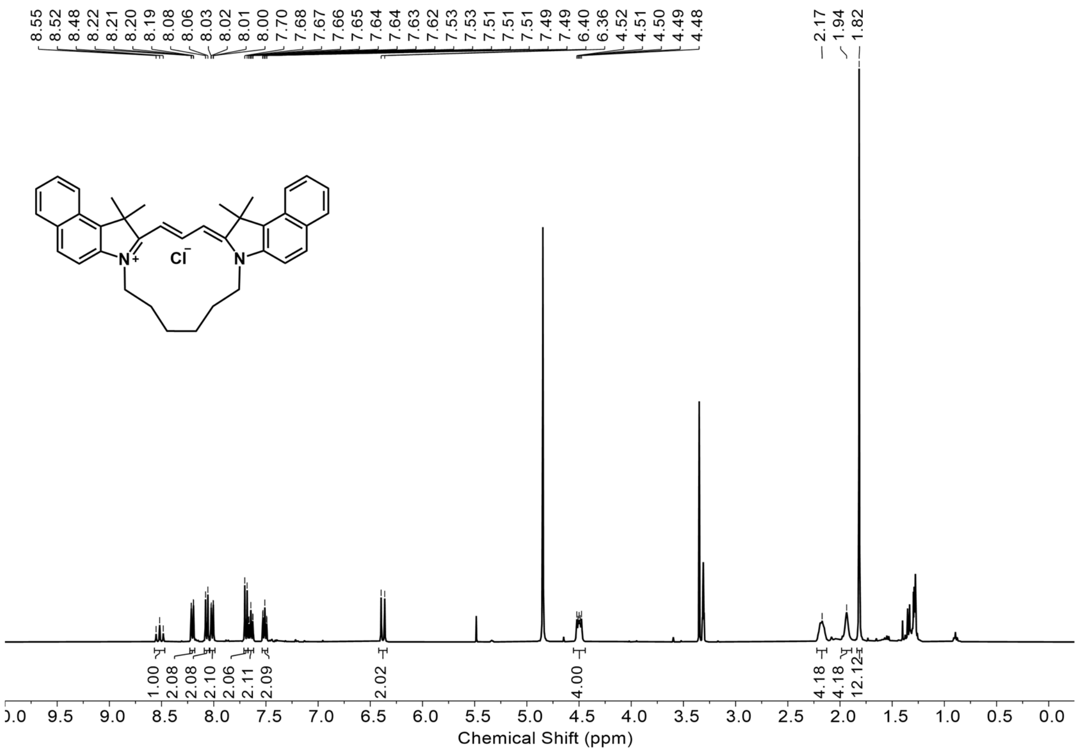

Figure S42. ^{1}H NMR spectrum of **CY-1** in CD_3OD-*d4* (400 MHz).

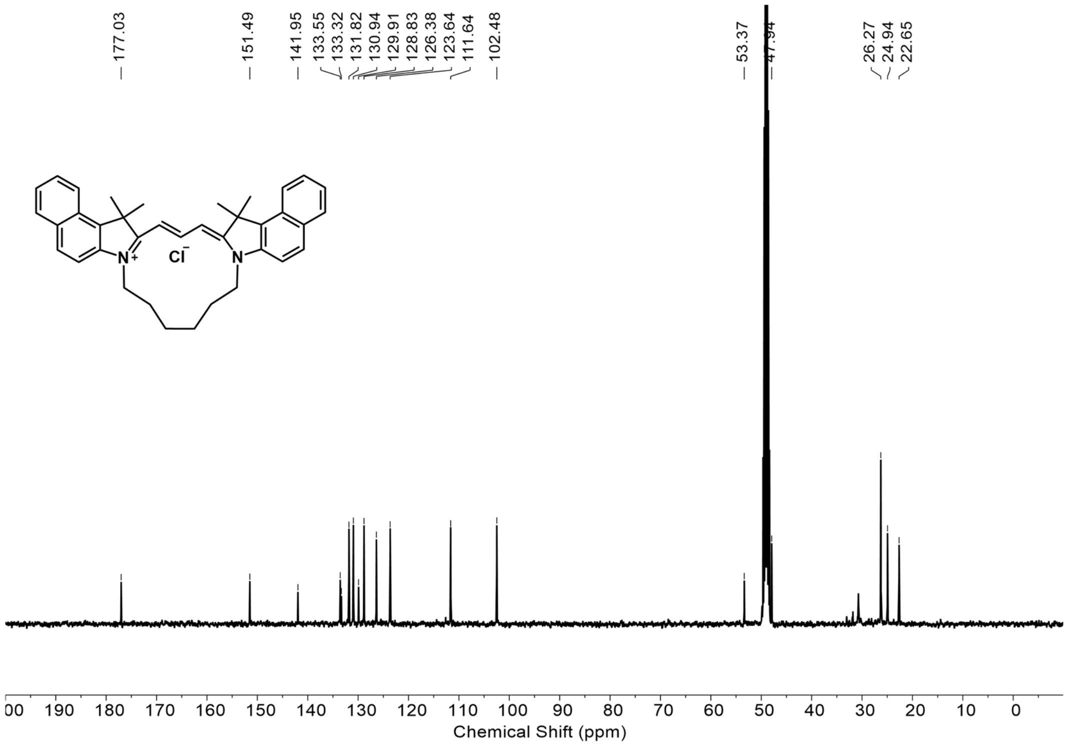

Figure S43. ^{13}C NMR spectrum of **CY-1** in CD_3OD-*d4* (101 MHz).

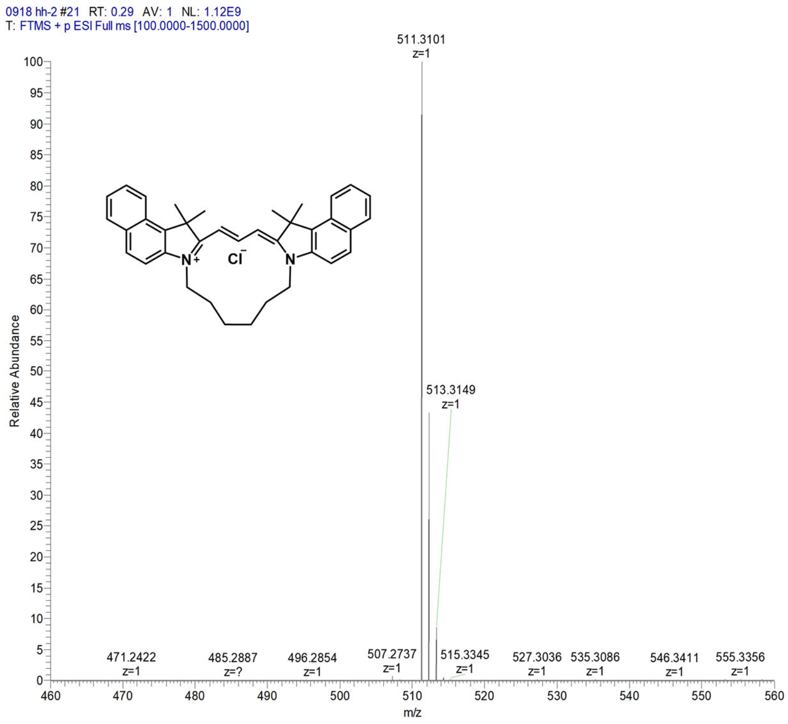

Figure S44. HRMS spectrum of **CY-1**.

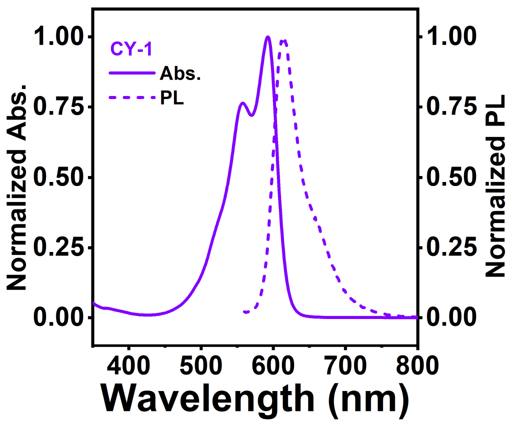

Figure S45. Normalized UV-Vis absorption and fluorescence spectra of **CY-1** in methanol.

Table S1. Reaction yield of metal-catalyzed CCClick reaction[a].

Entry	Metal salt	Equiv	Temperature (°C)	Entry	Yield[b] (%)
**1**	—	—	70	60 min	4
**2**	$CuCl_2$	0.5	70	40 min	49
**3**	CuCl	0.5	70	40 min	54
**4**	$FeCl_3$	1	70	20 min	54
**5**	$FeCl_2$	1	70	20 min	50
**6**	Hemin	1	70	60 min	85
**7**	—	—	37	12 h	6
**8**	$CuCl_2$	0.5	37	12 h	44
**9**	CuCl	0.5	37	12 h	48
**10**	$FeCl_3$	1	37	12 h	44
**11**	$FeCl_2$	1	37	12 h	42
**12**	Hemin	1	37	12 h	90

[a]Reaction conditions: **FB1** (1 mM), FA (10 mM) and diverse metal salt were stirred in DMF until reaction completion.

[b]Reaction yield was monitored by UV-vis absorption spectrum.

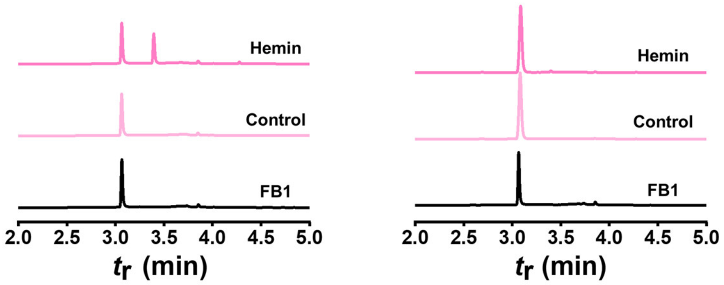

Figure S46. UPLC analysis (PDA 254 nm) of reaction mixture after dilution with H_2O/acetonitrile (V/V, 1:1). Reaction: **FB1** (1 mM), acetaldehyde (10 mM, left), benzaldehyde (10 mM, right) with/without hemin (1 mM) in DMF at 37°C for 4 h.

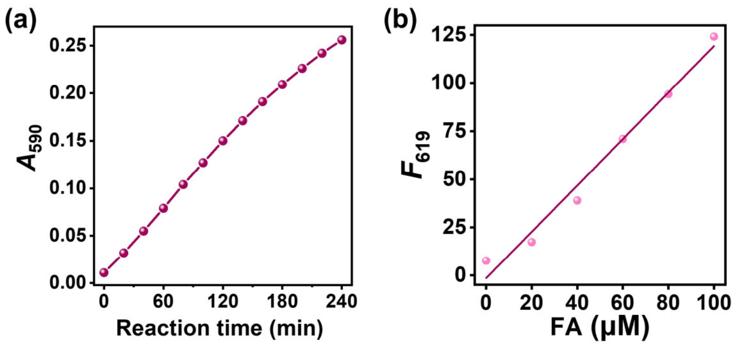

Figure S47. (a) Plot of A_{590} versus different reaction time. Reaction: **FB1** (30 μM) and FA (300 μM) with $FeCl_3$ (15 μM) in DMF at 37°C for 0–4 h. (b) Plot of $\boldsymbol{F_{619}}$ versus different FA concentrations. Reaction: **FB1** (10 μM) and FA (0–100 μM) with $FeCl_3$ (5 μM) in DMF at 37°C for 2 h.

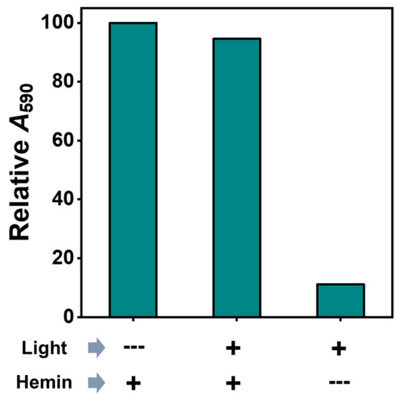

Figure S48. Relative absorption intensity at 590 nm (A_{590}) of solutions after continuous irradiation under white light (20 mW cm^{-2}, 4 h). Reaction: **FB1** (1 mM), FA (10 mM) and hemin (1 mM) in DMF at 37°C.

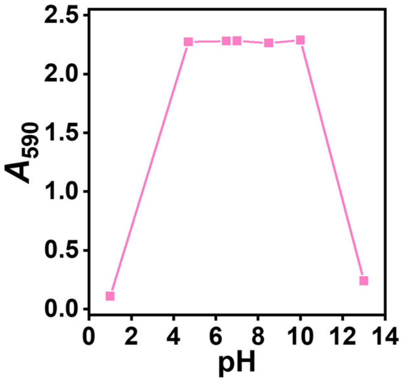

Figure S49. Absorption intensity at 590 nm (A_{590}) of solutions after 33.3-fold dilution of the reaction mixture with methanol as a function of pH. Reaction: **FB1** (1 mM), FA (10 mM) and hemin (1 mM) in 10% (*V*/*V*) water/DMF solvent at 37°C for 4 h.

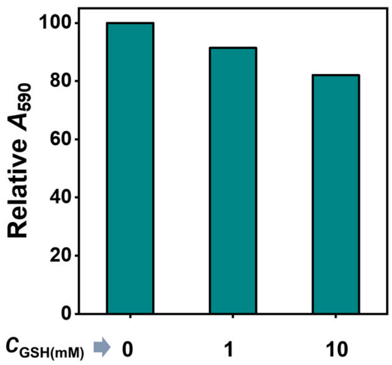

Figure S50. Relative absorption intensity at 590 nm (A_{590}) of solutions after 33.3-fold dilution of the reaction mixture with methanol. Reaction: **FB1** (1 mM), FA (10 mM), hemin (1 mM) and GSH (0−10 mM) in DMF at 37°C for 4 h.

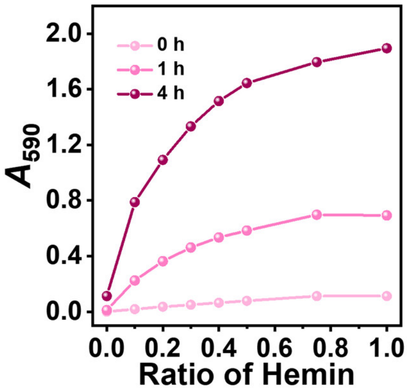

Figure S51. A_{590} of solutions after 33.3-fold dilution of the reaction mixture with methanol as a function of $R_{Hemin/FB1}$. Reaction: **FB1** (1 mM), FA (10 mM) and hemin (0-1 mM) in DMF at 37°C for 4 h.

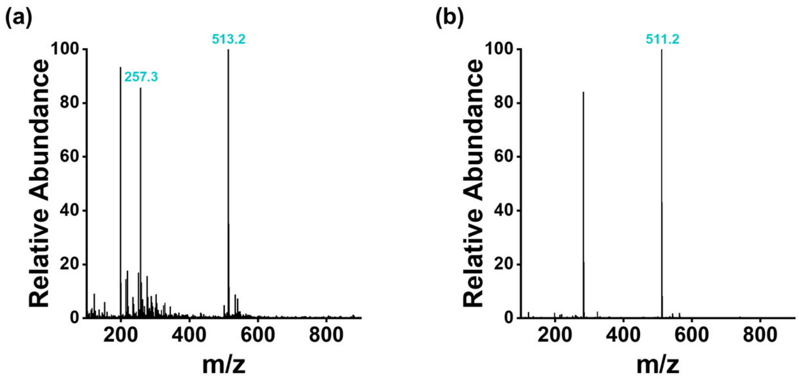

Figure S52. MS Spectra of **HCY-1** (a) and **CY-1** (b) recorded in UPLC-MS.

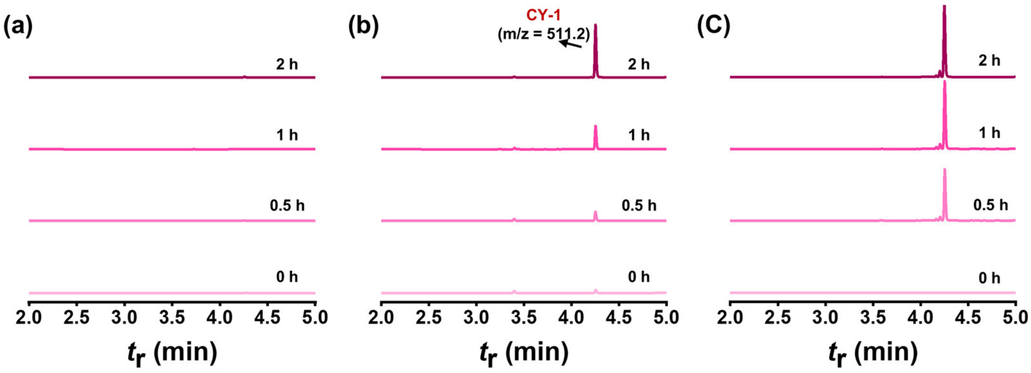

Figure S53. UPLC-MS analysis (PDA 590 nm) of reaction mixture diluted with H_2O/acetonitrile (V/V, 1:1). Reaction: **FB1** (1 mM) and FA (10 mM) without metal (a) /with hemin (0.5 mM, b) and $FeCl_3$ (0.5 mM, c) in DMF at 37°C.

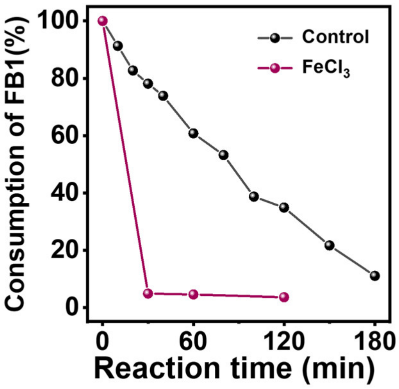

Figure S54. The consumption of **FB1** characterized by UPLC (PDA 254 nm). Reaction mixture was diluted with H_2O/acetonitrile (V/V, 1: 1) before analysis. Reaction: **FB1** (1 mM) and FA (10 mM) with/without $FeCl_3$ (0.5 mM) at 37°C for 0–180 min.

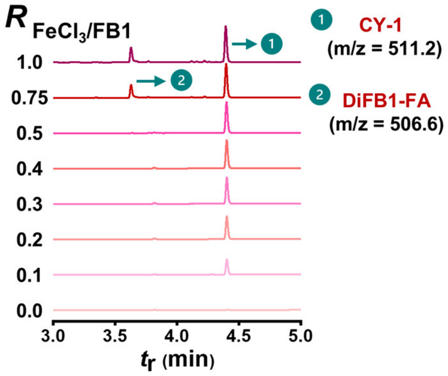

Figure S55. UPLC-MS analysis (590 nm) of reaction mixtures containing **FB1** (1 mM), FA (1 mM) and $FeCl_3$ (0-1 mM) in DMF at 37°C for 0.5 h.

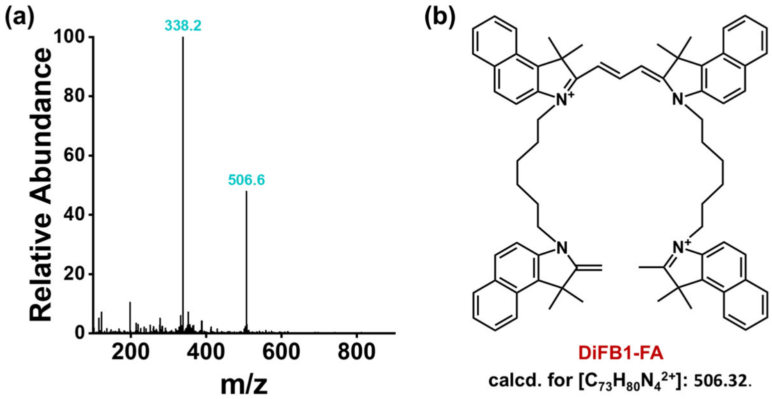

Figure S56. (a) MS Spectra of **DiFB1-FA** recorded in UPLC-MS. (b) The structure of **DiFB1-FA** adduct.

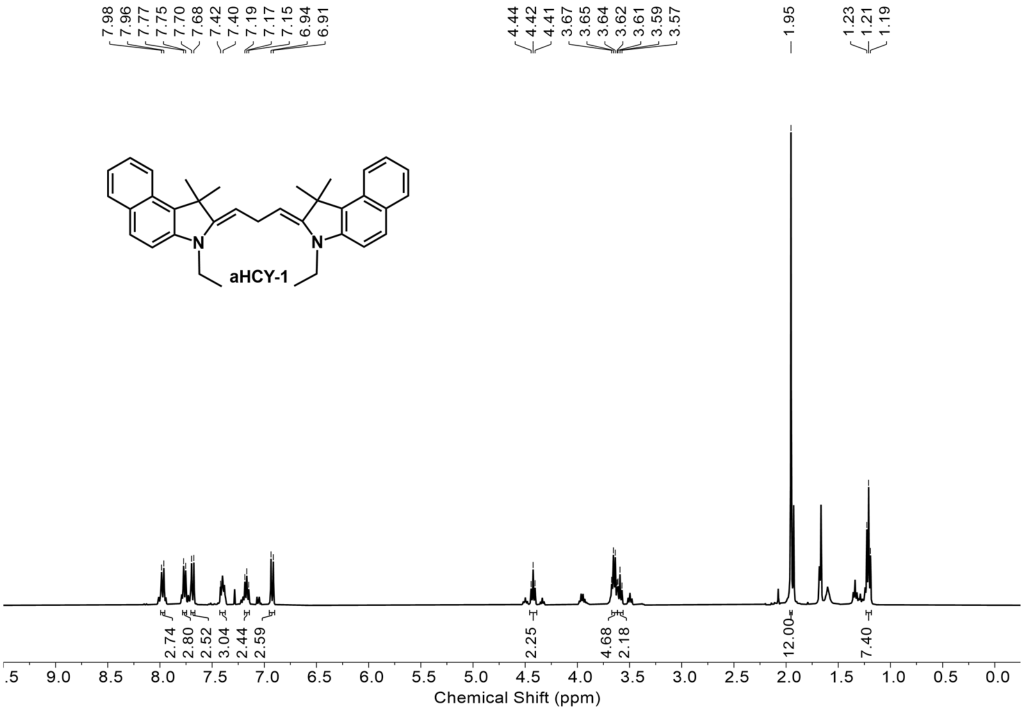

Figure S57. ^{1}H NMR spectrum of **aHCY-I** in $CDCl_3$ (400 MHz).

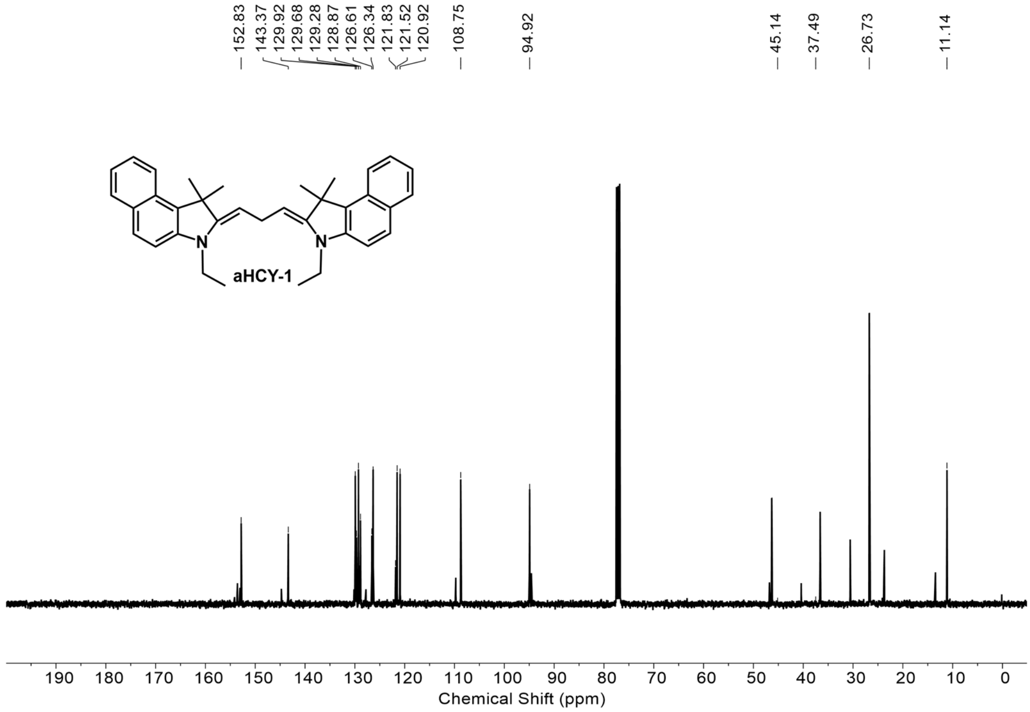

Figure S58. ^{13}C NMR spectrum of **aHCY-I** in $CDCl_3$ (101 MHz).

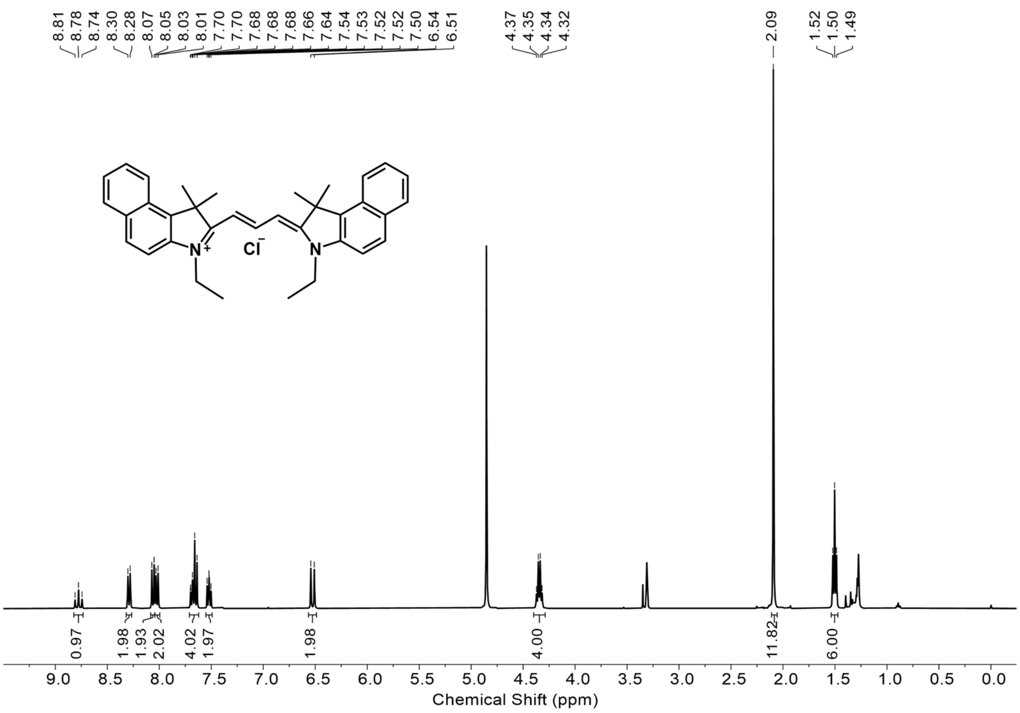

Figure S59. ^{1}H NMR spectrum of **aCY-1** in CD_3OD-*d4* (400 MHz).

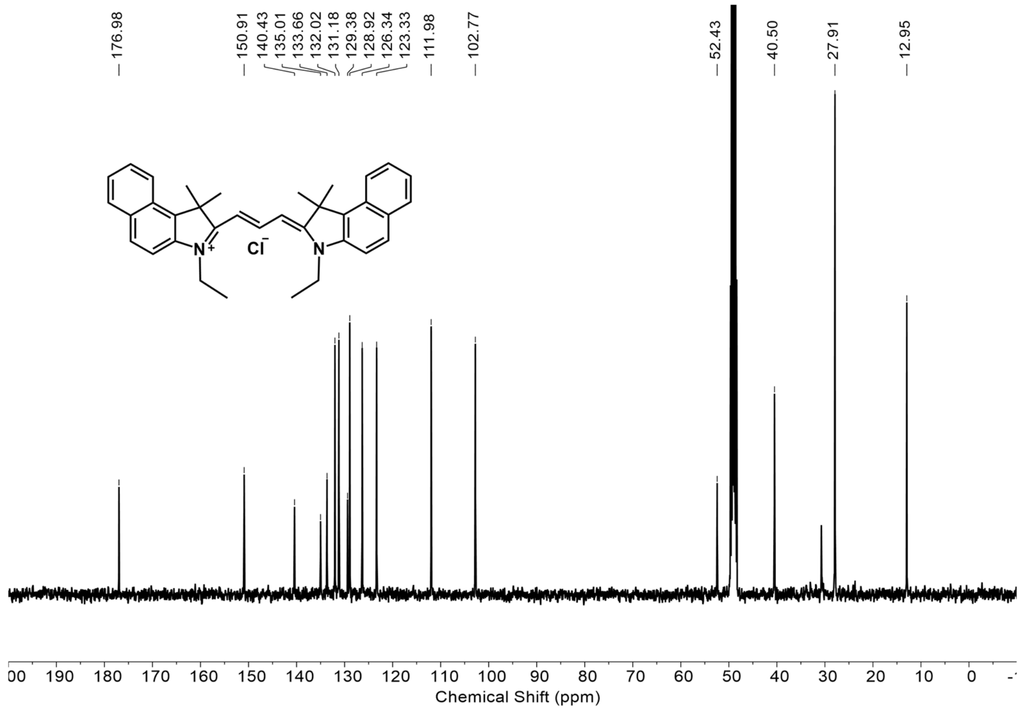

Figure S60. ^{13}C NMR spectrum of **aCY-1** in CD_3OD-*d4* (101 MHz).

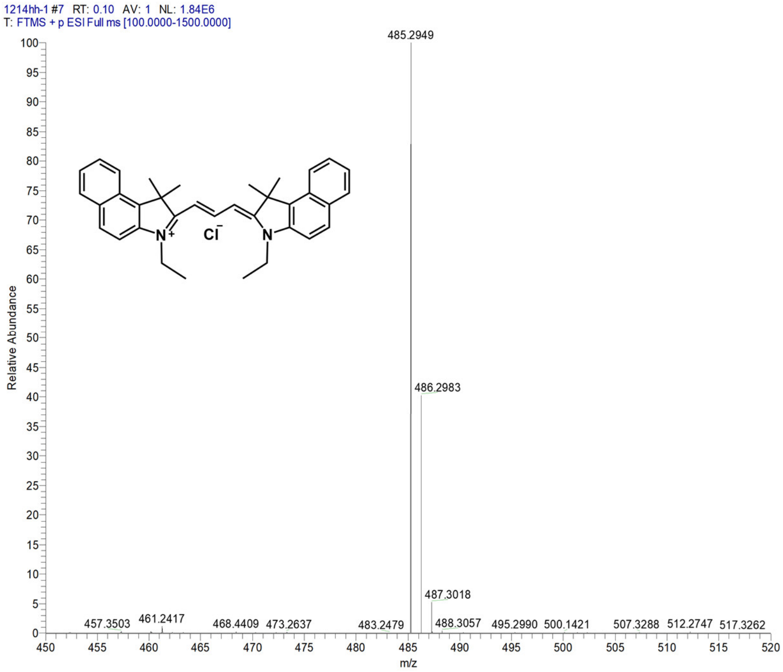

Figure S61. HRMS spectrum of **aCY-1**.

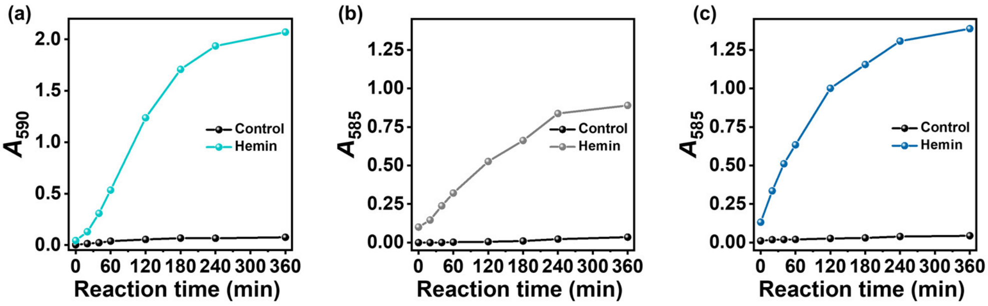

Figure S62. (a) The A_{590} of the reaction mixture containing FB1 (1 mM), FA (10 mM) and hemin (1 mM) in DMF at 37°C for different times and 33.3-fold diluted with methanol. (b) The A_{585} of the reaction mixture containing uniFB1 (2 mM), FA (10 mM) and hemin (1 mM) in DMF at 37°C for different times and 66.7-fold diluted with methanol. (c) The A_{585} of the reaction mixture containing aHCY-1 (1 mM) and hemin (1 mM) in DMF at 37°C for different times and 66.7-fold diluted with methanol.

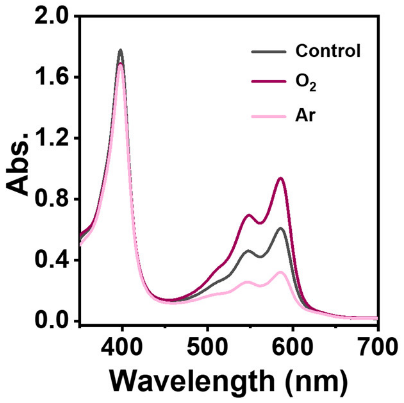

Figure S63. The UV-Vis absorption spectra of the reaction mixture after 66.7-fold dilution with methanol. Reaction: **aHCY-1** (1 mM) and hemin (1 mM) in DMF at 37°C for 1 h with different oxygen content.

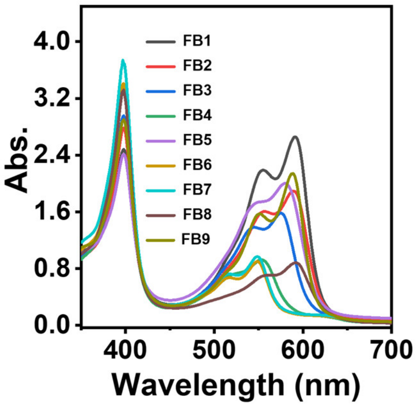

Figure S64. The UV-Vis absorption spectra of the reaction mixture after 33.3-fold dilution with methanol. Reaction: **FB1−FB9** (1 mM), FA (10 mM) and hemin (1 mM) in DMF at 37°C for 12 h.

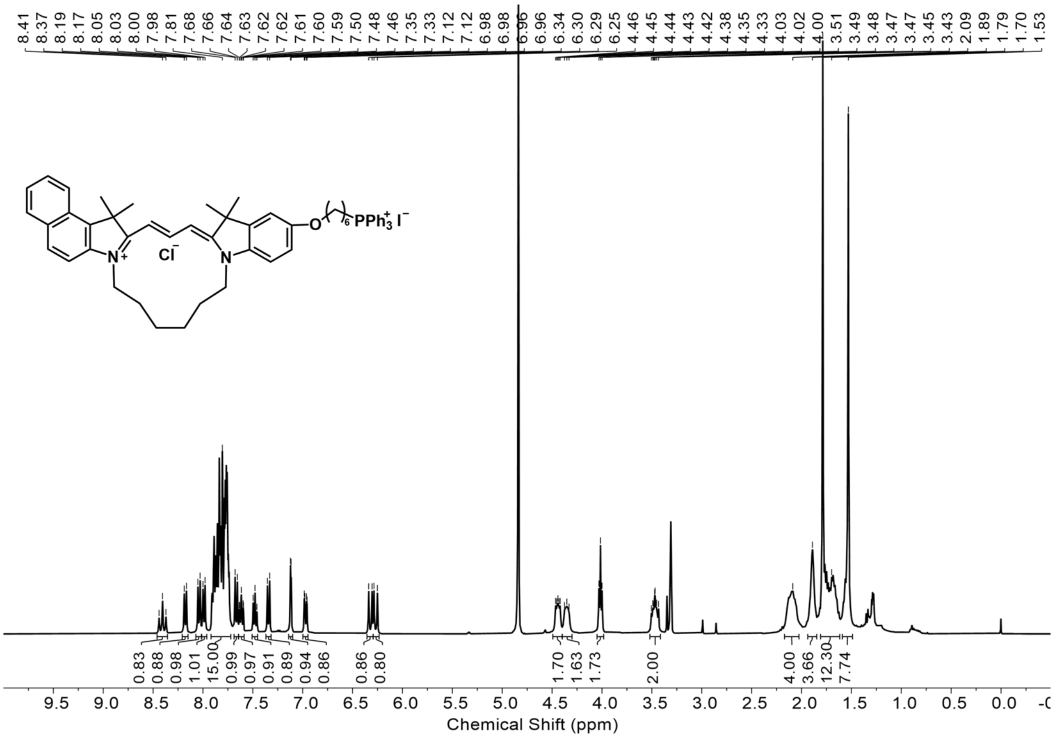

Figure S65. ^{1}H NMR spectrum of **CY-2** in CD_3OD-*d4* (400 MHz).

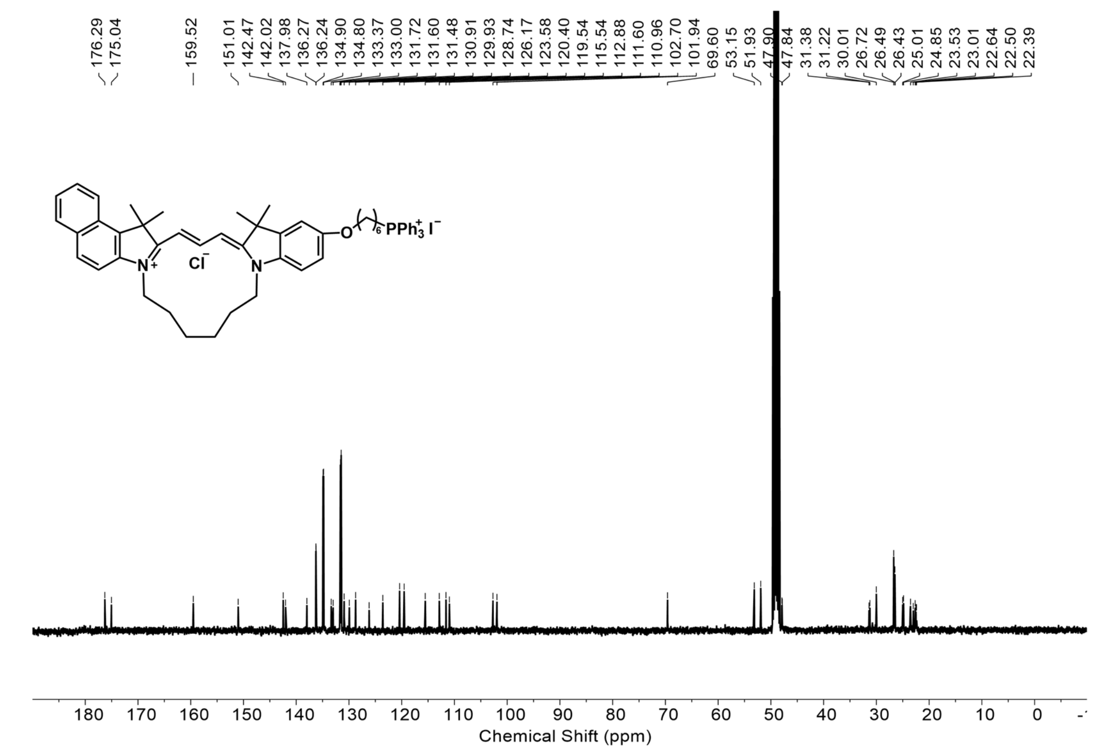

Figure S66. ^{13}C NMR spectrum of **CY-2** in CD_3OD-*d4* (101 MHz).

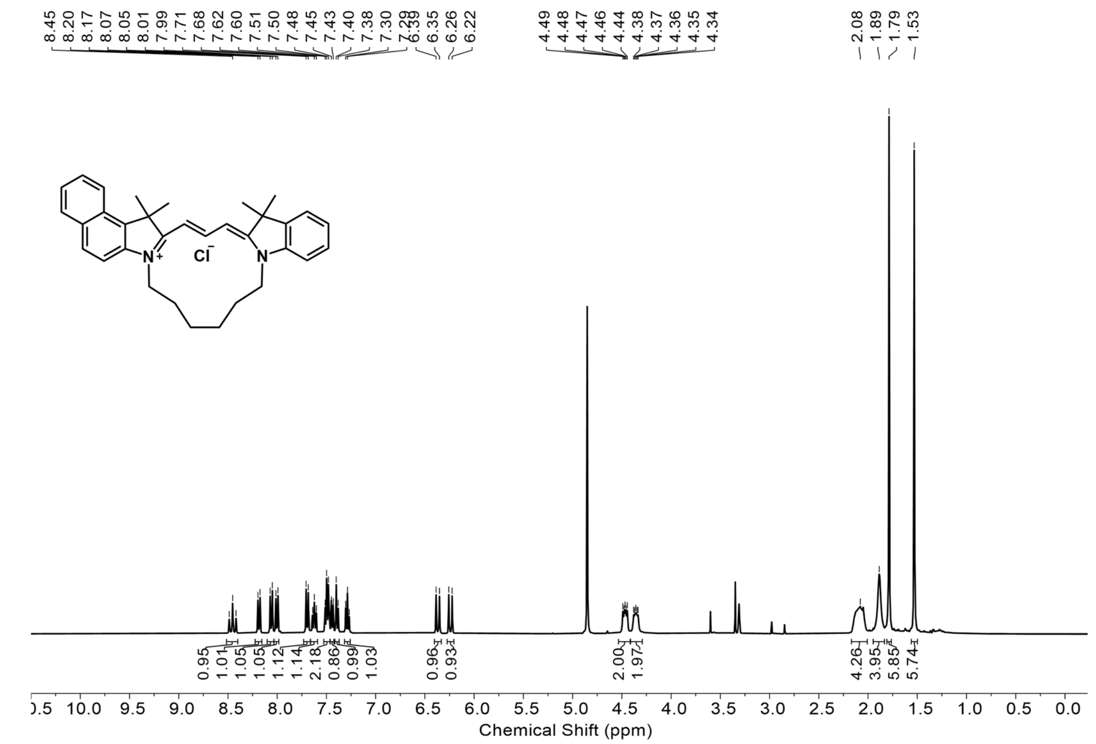

Figure S67. ^{1}H NMR spectrum of **CY-3** in CD_3OD-*d4* (400 MHz).

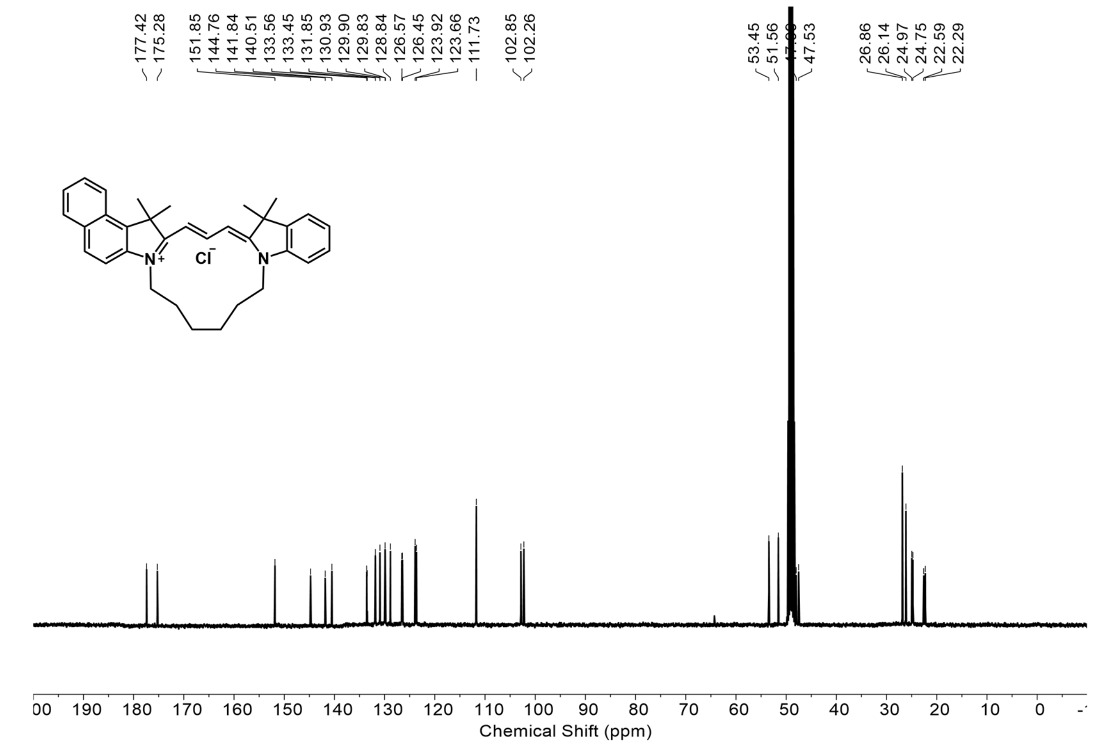

Figure S68. ^{13}C NMR spectrum of **CY-3** in CD_3OD-*d4* (101 MHz).

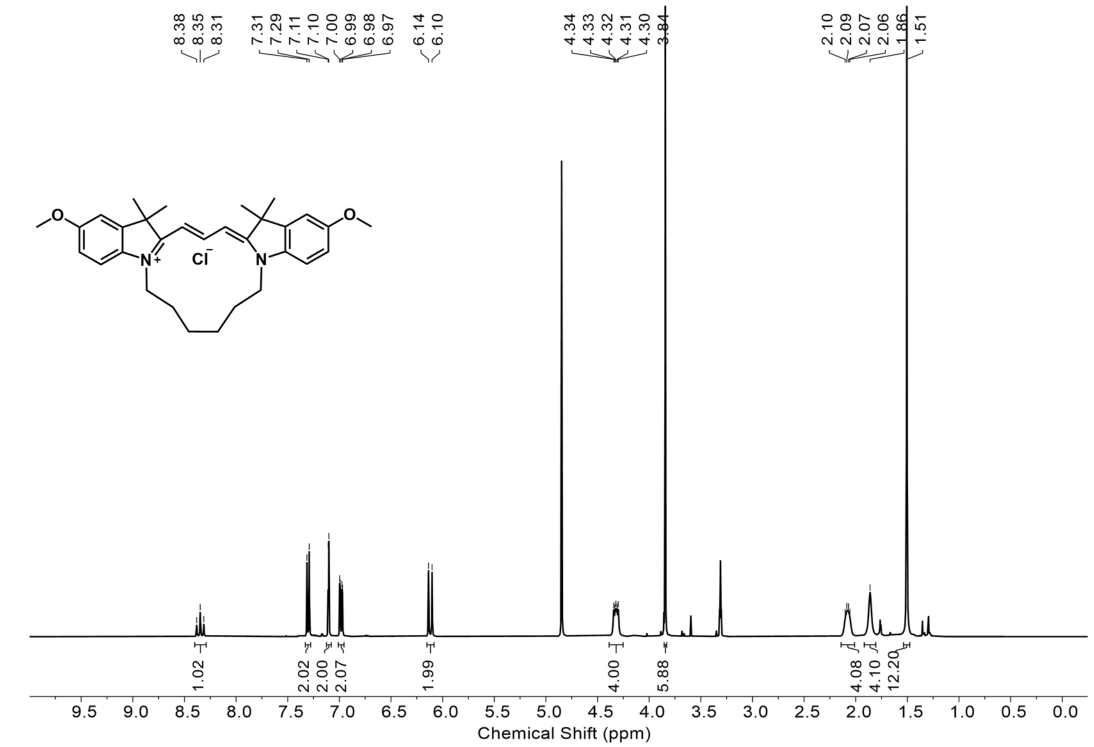

Figure S69. ^{1}H NMR spectrum of **CY-4** in CD_3OD-*d4* (400 MHz).

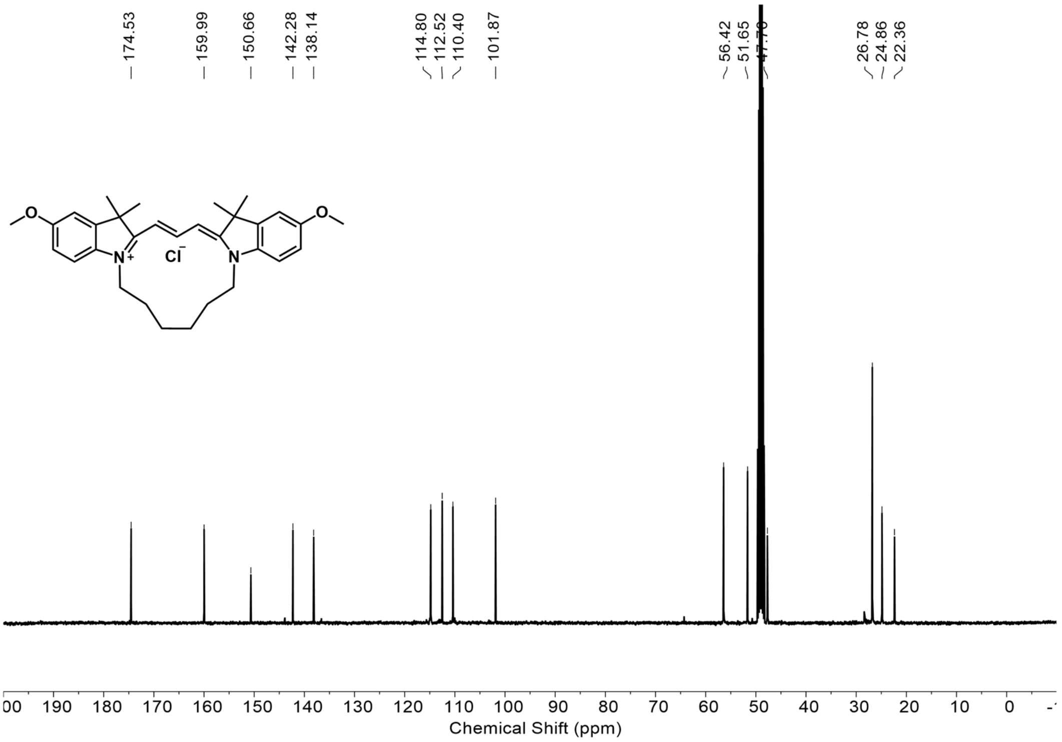

Figure S70. ^{13}C NMR spectrum of **CY-4** in CD_3OD-*d4* (101 MHz).

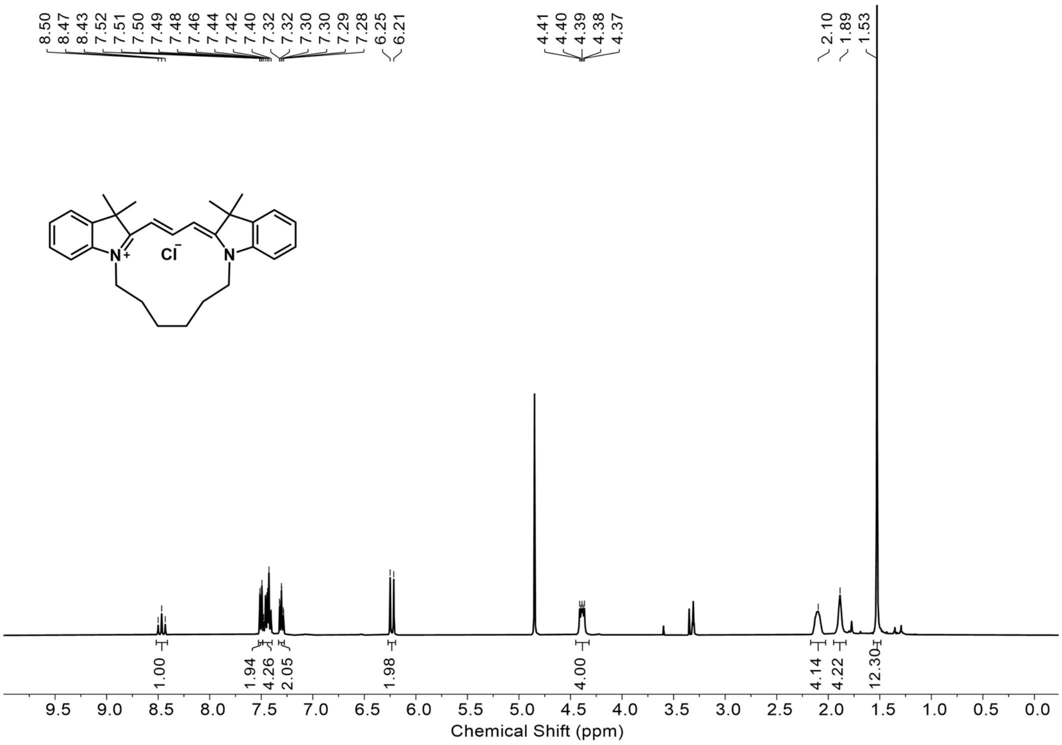

Figure S71. ^{1}H NMR spectrum of **CY-5** in CD_3OD-*d4* (400 MHz).

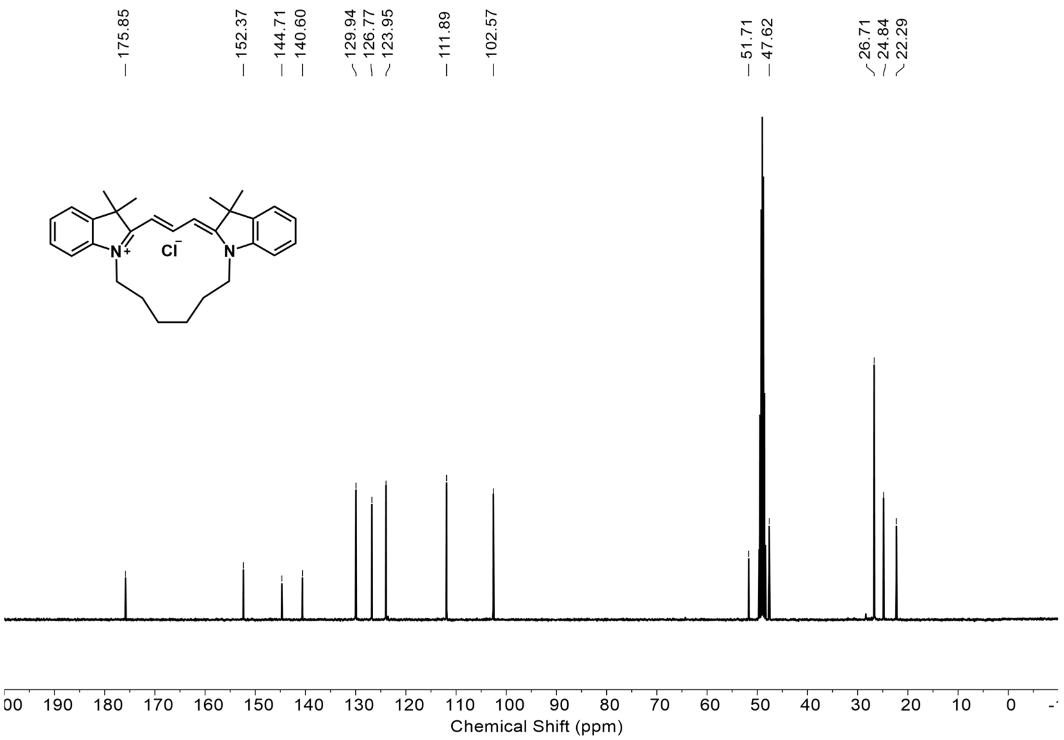

Figure S72. ^{13}C NMR spectrum of **CY-5** in CD_3OD-*d4* (101 MHz).

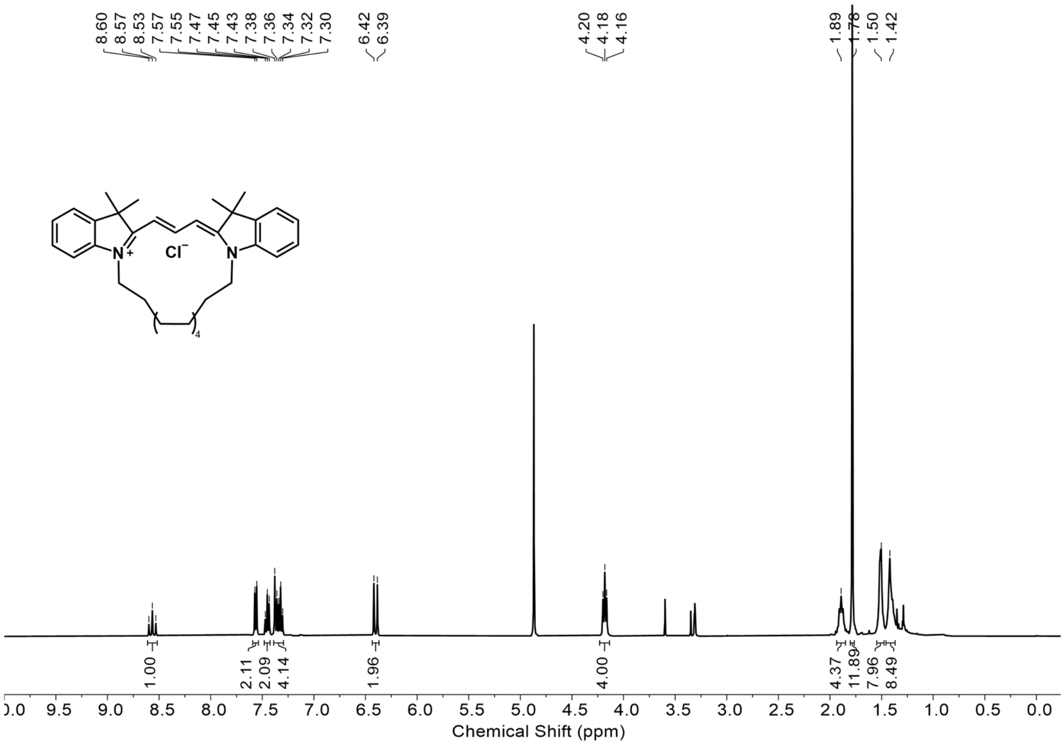

Figure S73. ^{1}H NMR spectrum of **CY-6** in CD_3OD-*d4* (400 MHz).

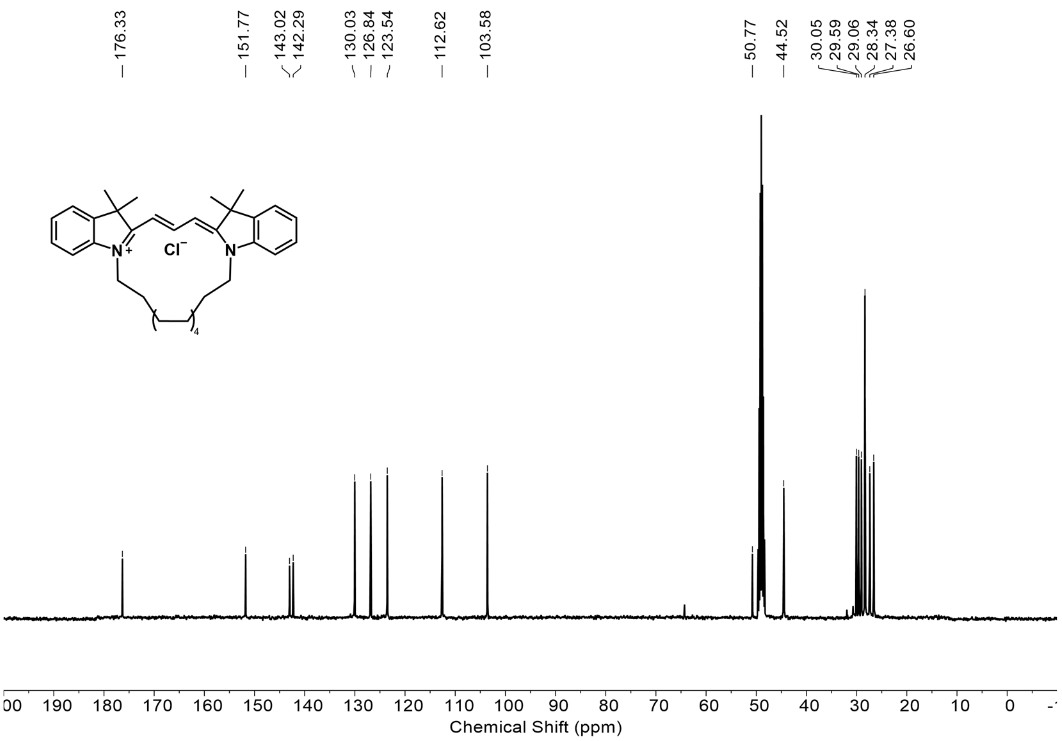

Figure S74. ^{13}C NMR spectrum of **CY-6** in CD_3OD-*d4* (101 MHz).

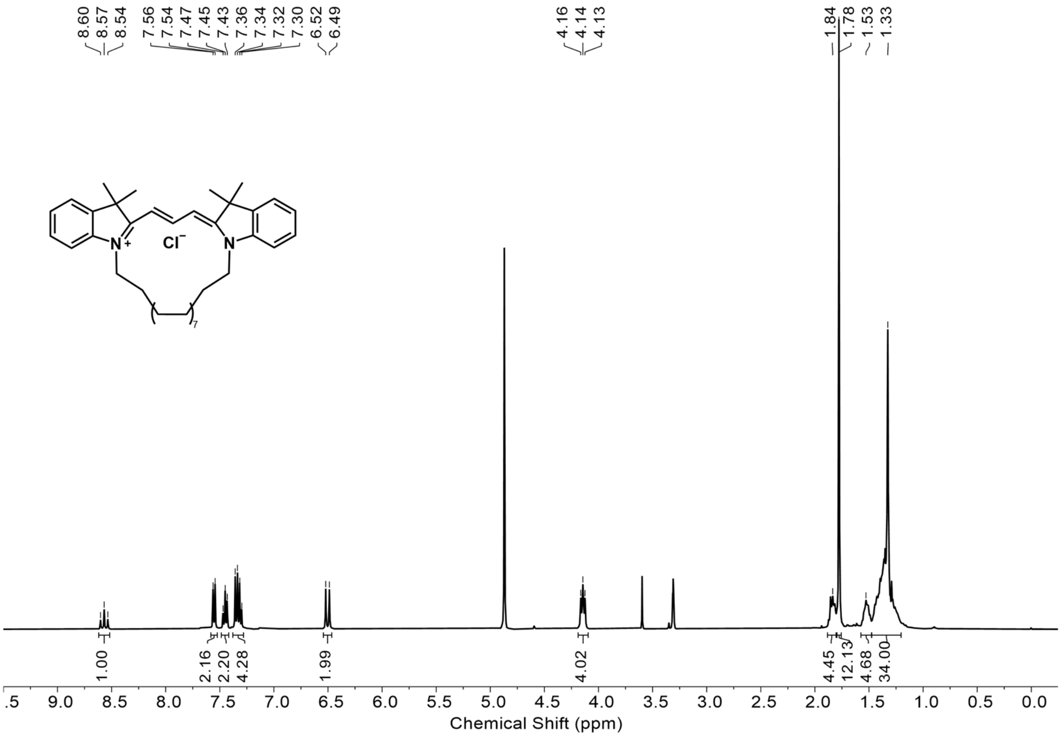

Figure S75. ^{1}H NMR spectrum of **CY-7** in CD_3OD-*d4* (400 MHz).

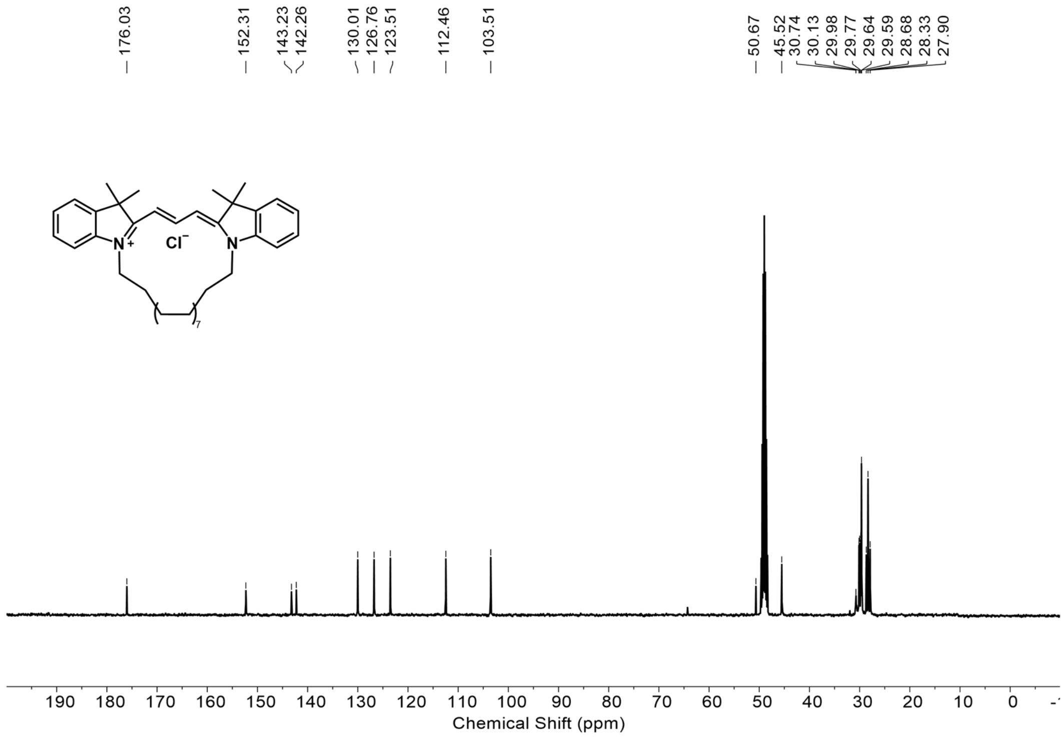

Figure S76. ^{13}C NMR spectrum of **CY-7** in CD_3OD-*d4* (101 MHz).

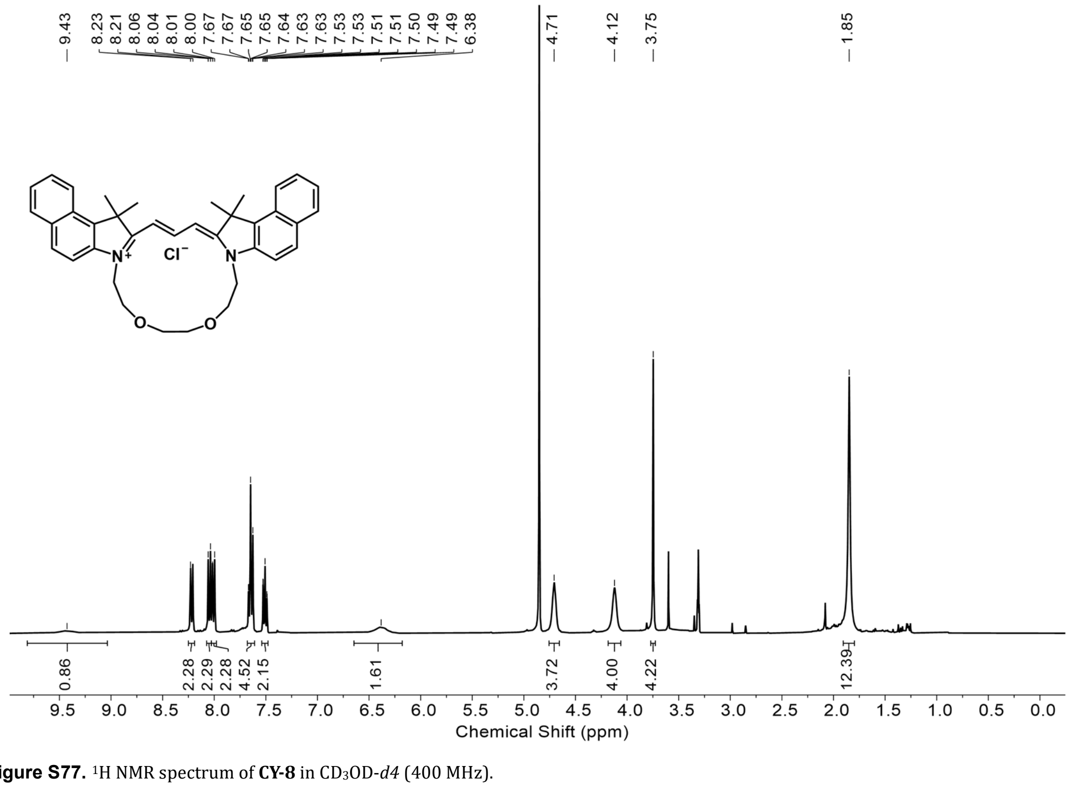

Figure S77. ^{1}H NMR spectrum of **CY-8** in CD_3OD-*d4* (400 MHz).

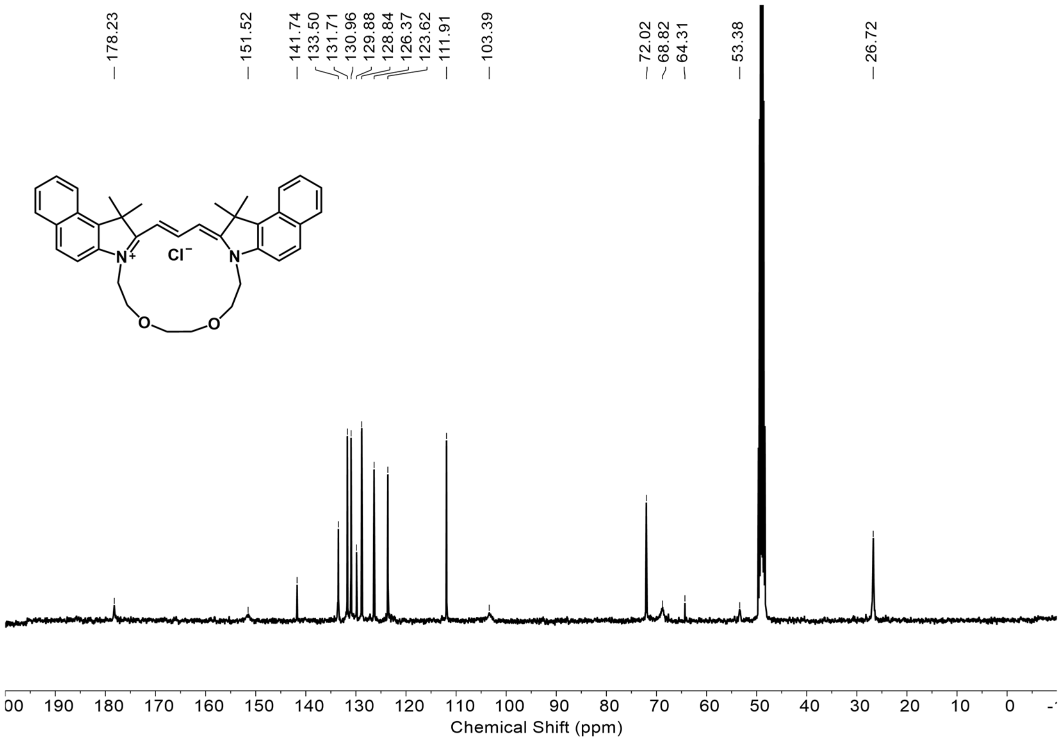

Figure S78. ^{13}C NMR spectrum of **CY-8** in CD_3OD-*d4* (101 MHz).

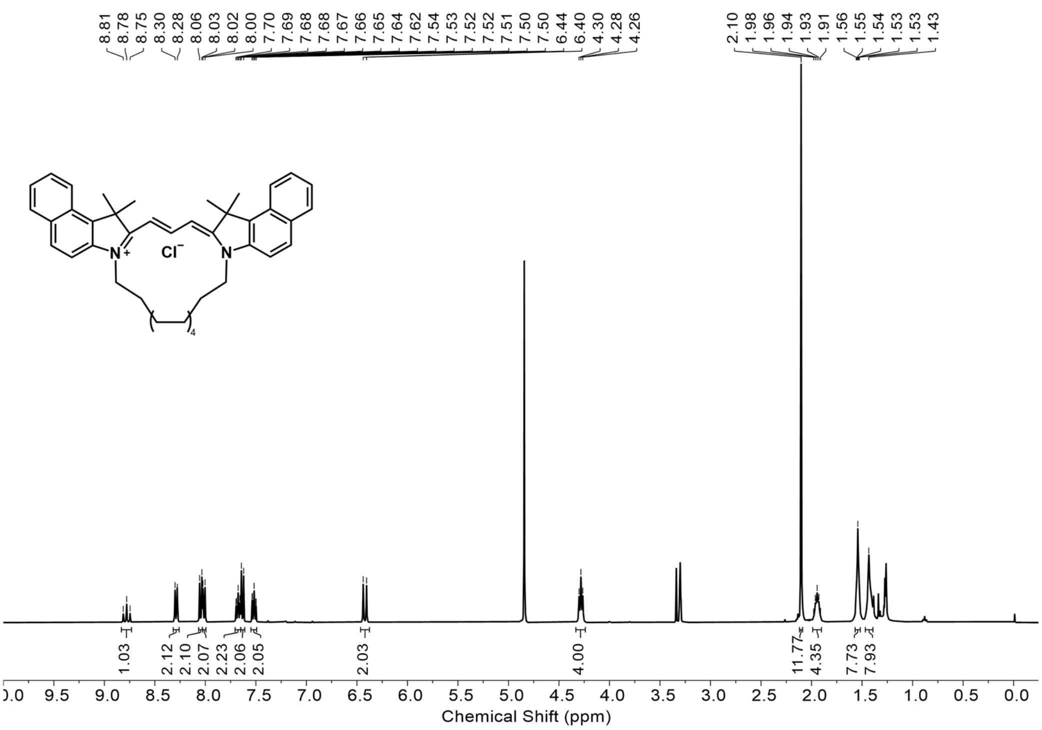

Figure S79. ^{1}H NMR spectrum of **CY-9** in CD_3OD-*d4* (400 MHz).

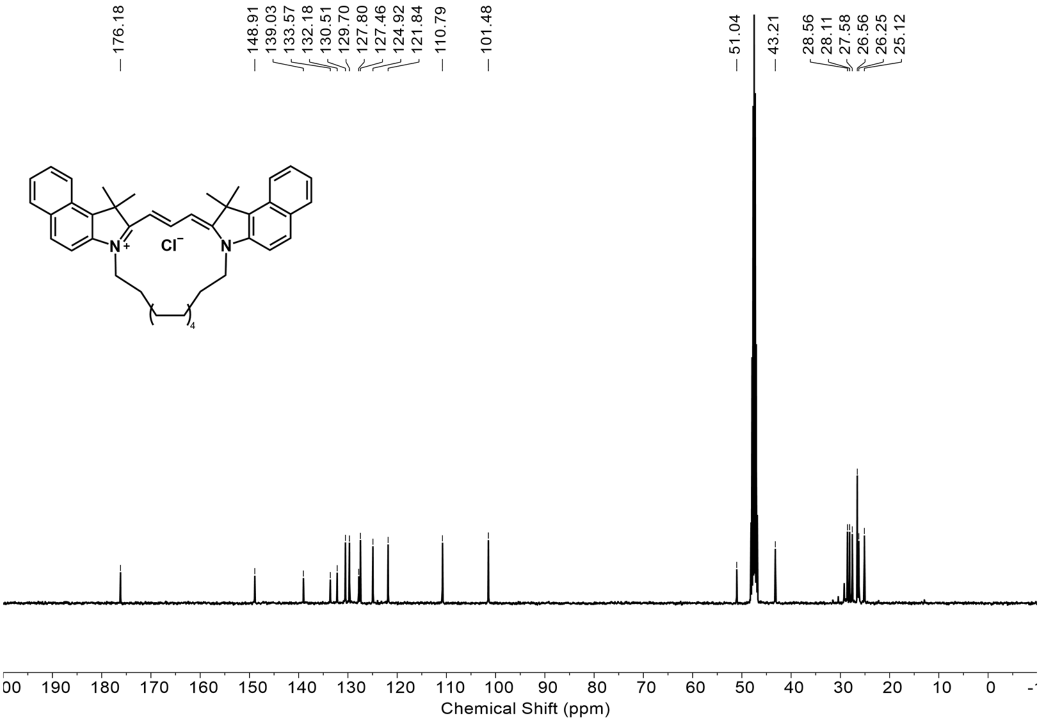

Figure S80. ^{13}C NMR spectrum of **CY-9** in CD_3OD-*d4* (101 MHz).

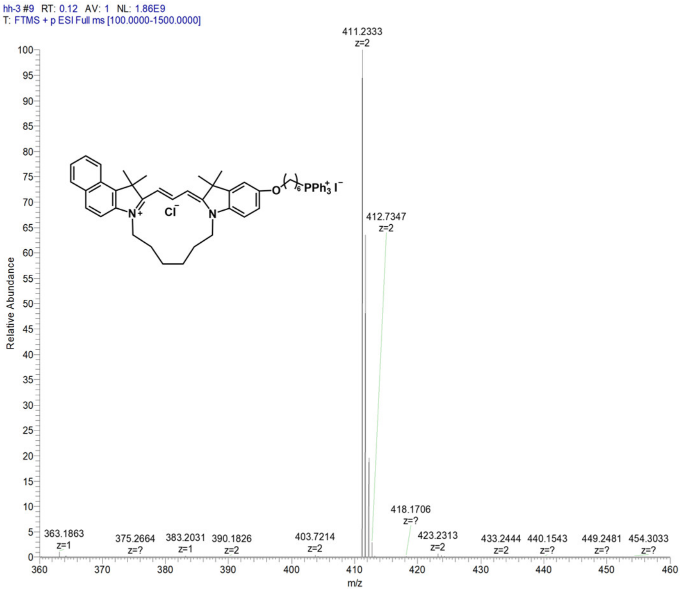

Figure S81. HRMS spectrum of **CY-2**.

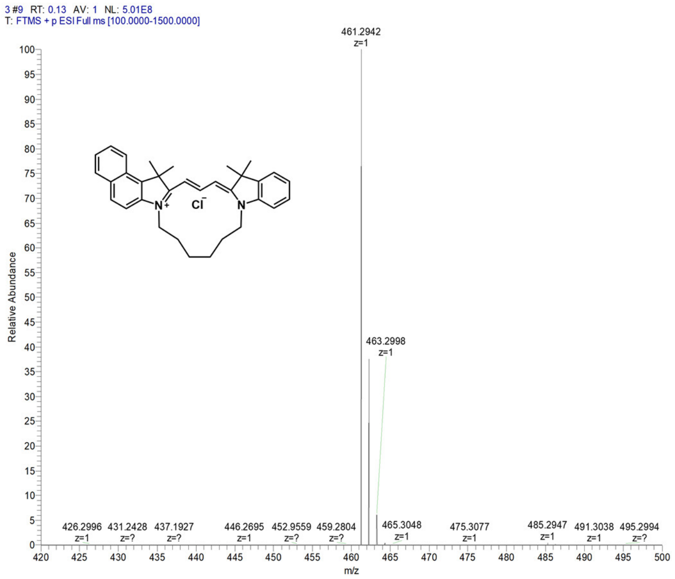

Figure S82. HRMS spectrum of **CY-3**.

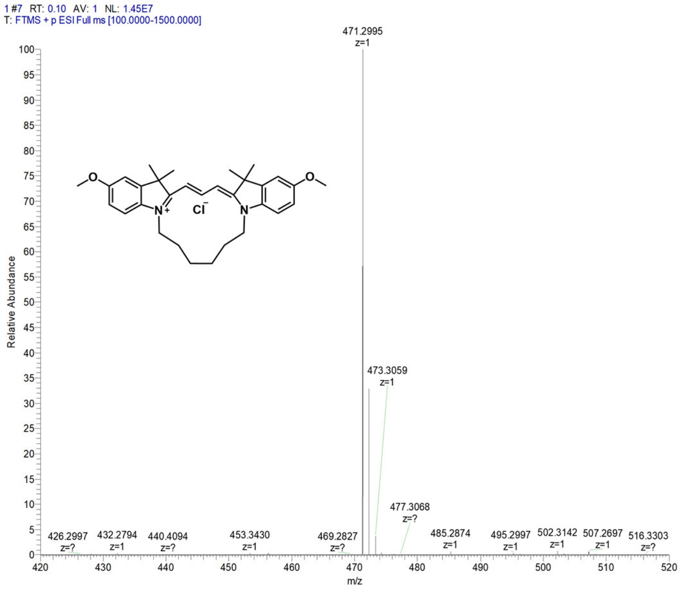

Figure S83. HRMS spectrum of **CY-4**.

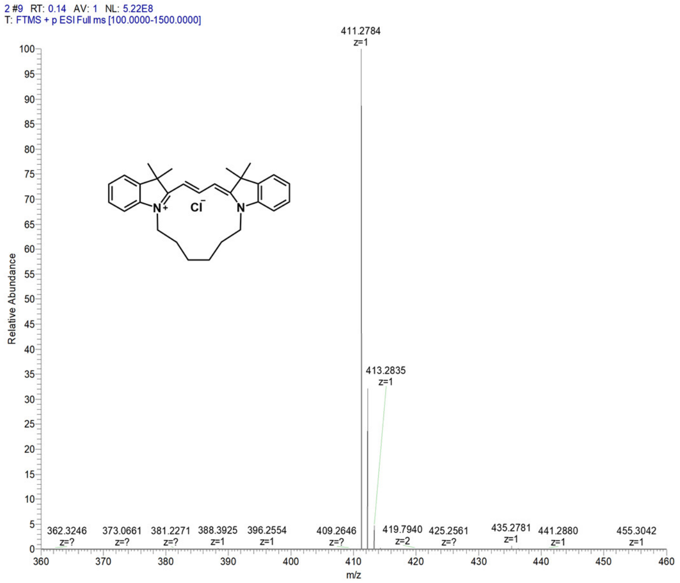

Figure S84. HRMS spectrum of **CY-5**.

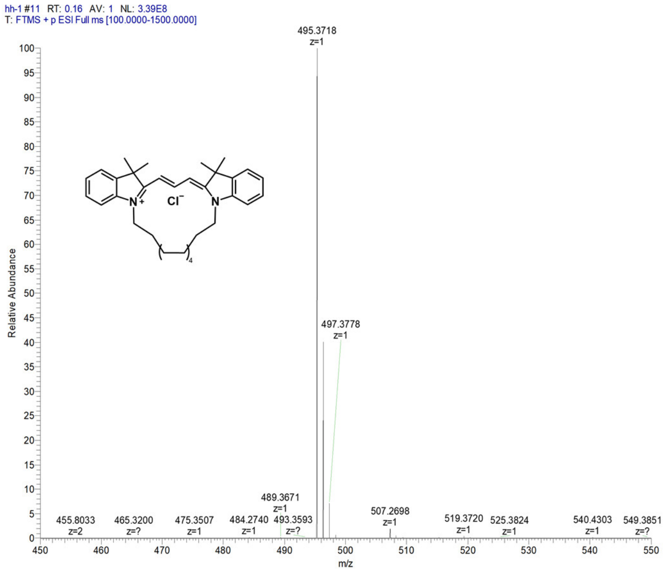

Figure S85. HRMS spectrum of **CY-6**.

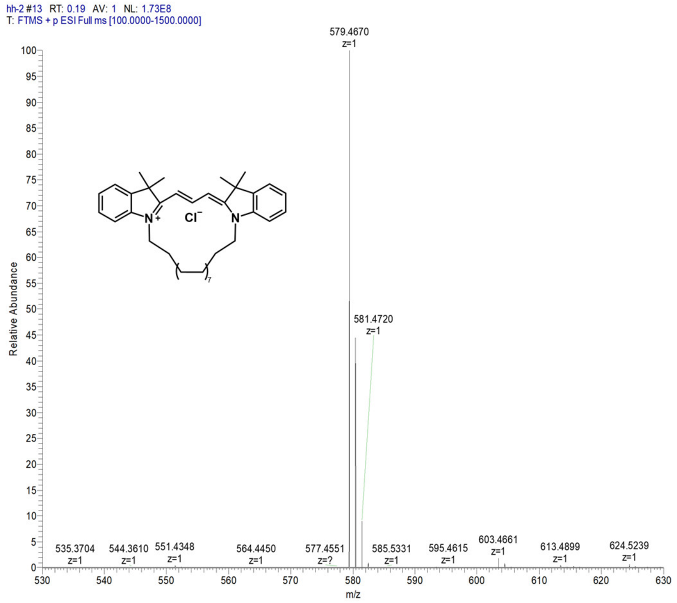

Figure S86. HRMS spectrum of **CY-7**.

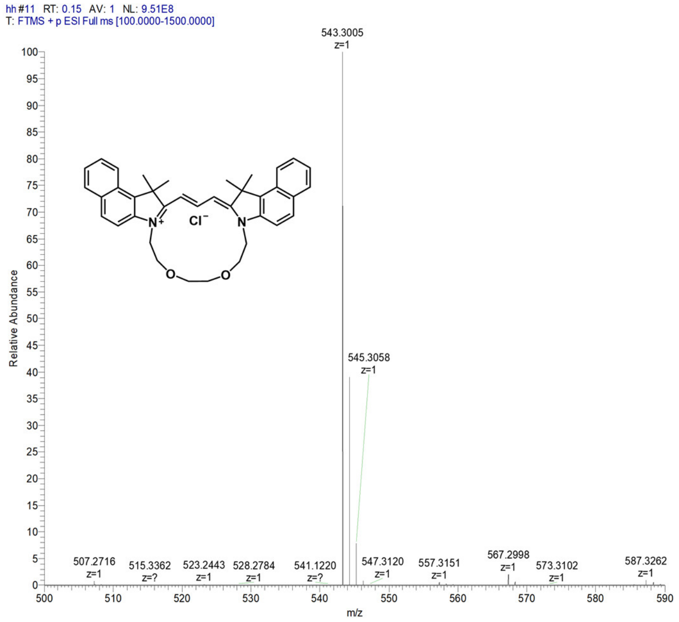

Figure S87. HRMS spectrum of **CY-8**.

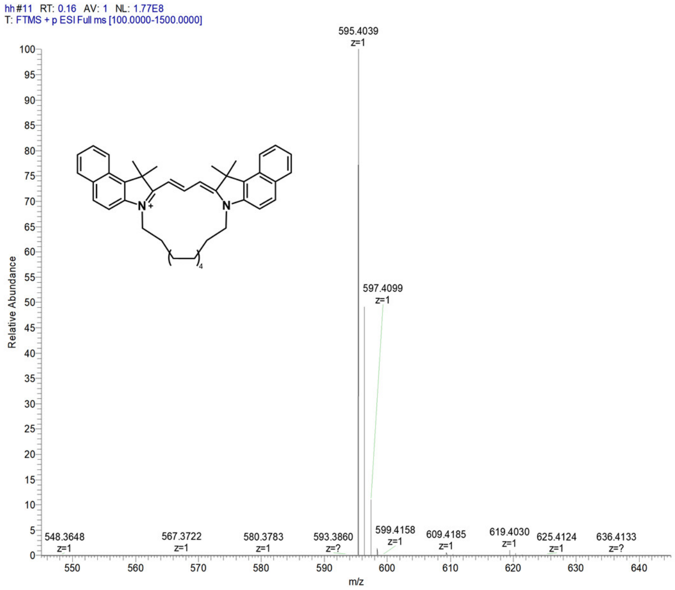

Figure S88. HRMS spectrum of **CY-9**.

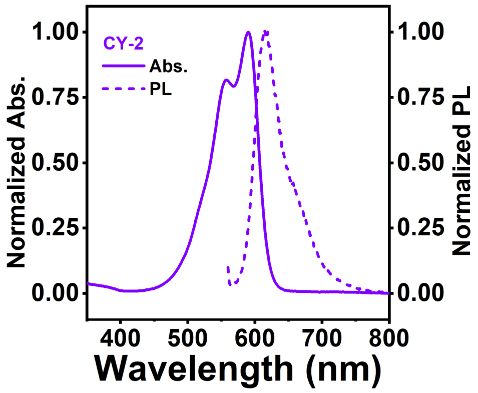

Figure S89. Normalized UV-Vis absorption and fluorescence spectra of **CY-2** in methanol.

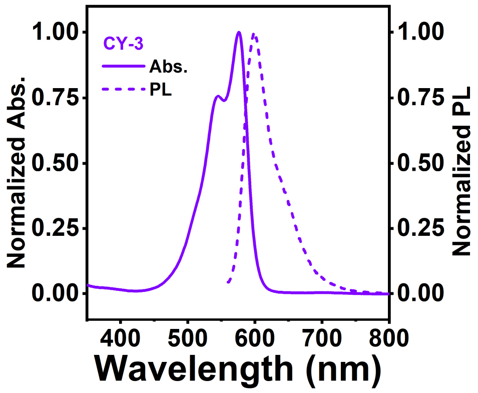

Figure S90. Normalized UV-Vis absorption and fluorescence spectra of **CY-3** in methanol.

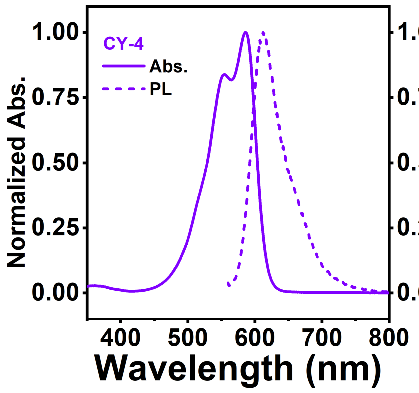

Figure S91. Normalized UV-Vis absorption and fluorescence spectra of **CY-4** in methanol.

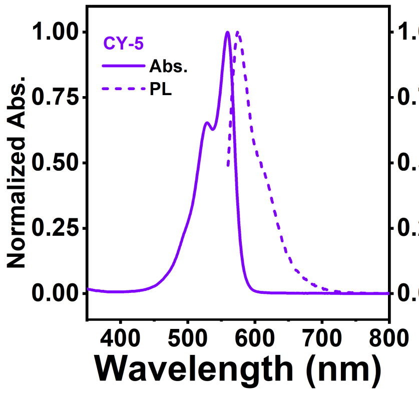

Figure S92. Normalized UV-Vis absorption spectra and fluorescence spectra of **CY-5** in methanol.

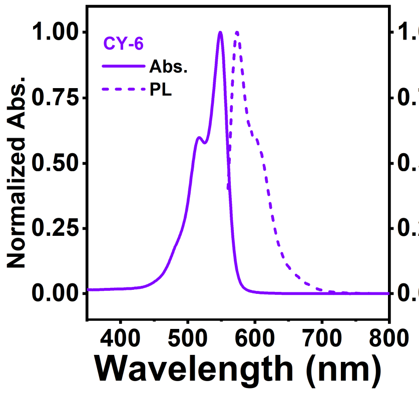

Figure S93. Normalized UV-Vis absorption and fluorescence spectra of **CY-6** in methanol.

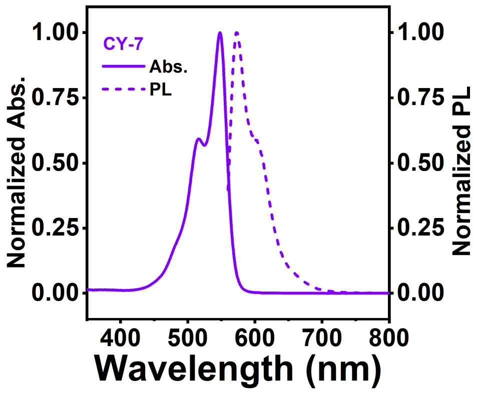

Figure S94. Normalized UV-Vis absorption and fluorescence spectra of **CY-7** in methanol.

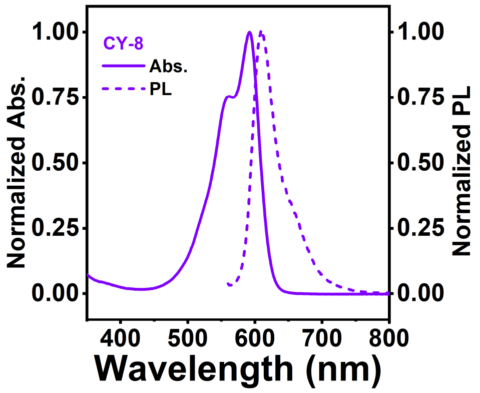

Figure S95. Normalized UV-Vis absorption and fluorescence spectra of **CY-8** in methanol.

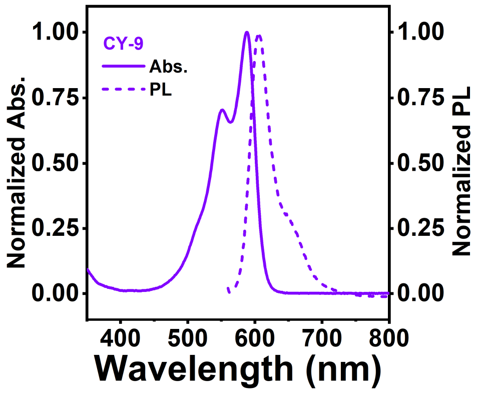

Figure S96. Normalized UV-Vis absorption and fluorescence spectra of **CY-9** in methanol.

Table S2. Photophysical Properties of Cycic Cyanines in Methanol.

Cyanine	λ_{abs}, nm	λ_{em}, nm	ε, $M^{-1}\cdot cm^{-1}$	Φ_f(%)[a]
**CY-1**	590	615	9.78	27
**CY-2**	590	619	6.65	7.9
**CY-3**	576	599	10.6	15
**CY-4**	586	615	7.50	8.6
**CY-5**	559	575	9.10	8.8
**CY-6**	548	573	9.99	13
**CY-7**	548	573	11.5	12
**CY-8**	591	610	7.70	11
**CY-9**	588	605	8.81	36

[a]Fluorescence quantum yield of **CY-1−CY-4 and CY-8−CY-9** were measured in DCM using Cresyl Violet (Φ_F = 0.54 in methanol at 25°C) as the reference; fluorescence quantum yield of **CY-5−CY-7** were measured in DCM using Rhodamine B (Φ_F = 0.66 in ethanol at 25°C) as the reference.[4]

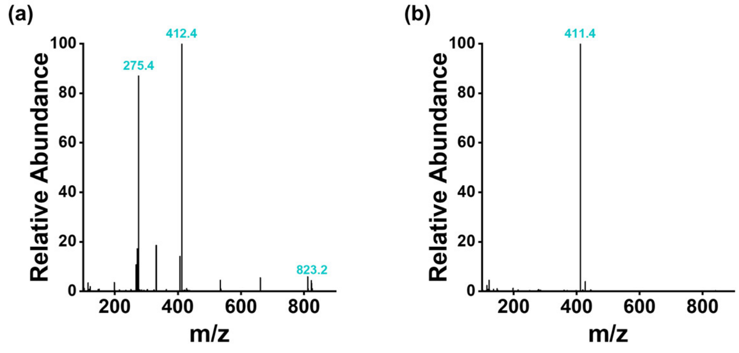

Figure S97. MS spectra of **HCY-2** (a) and **CY-2** (b) recorded by UPLC-MS.

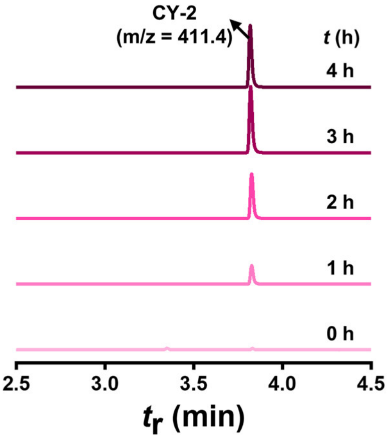

Figure S98. UPLC-MS analysis of reaction mixture recorded in a 590 nm channel PDA detector. Reaction: **FB2** (1 mM) and FA (10 mM) with hemin (1 mM) in DMF at 37°C for 0−4 h.

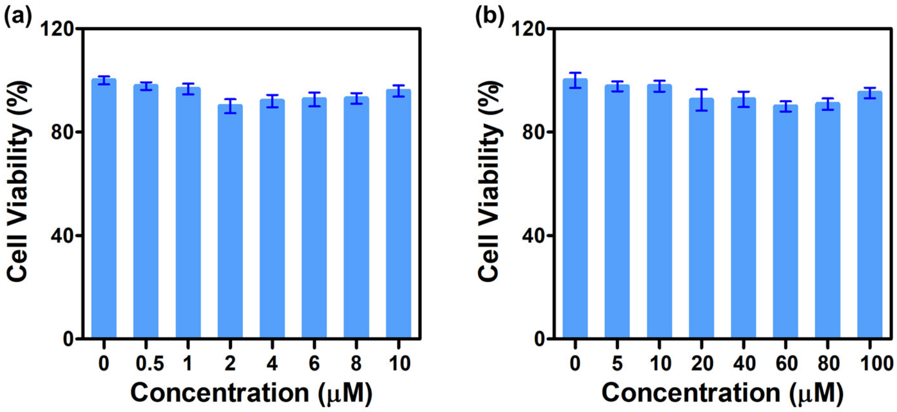

Figure S99. Viabilities of PC12 cells incubated with different concentrations of hemin (a) or FA (b) for 24 h.

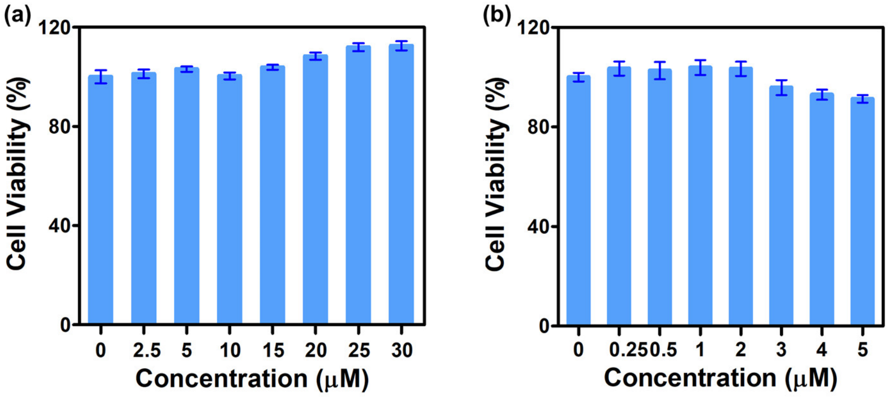

Figure S100. Viabilities of PC12 cells incubated with different concentrations of **FB1** (a) or **FB2** (b) for 24 h.

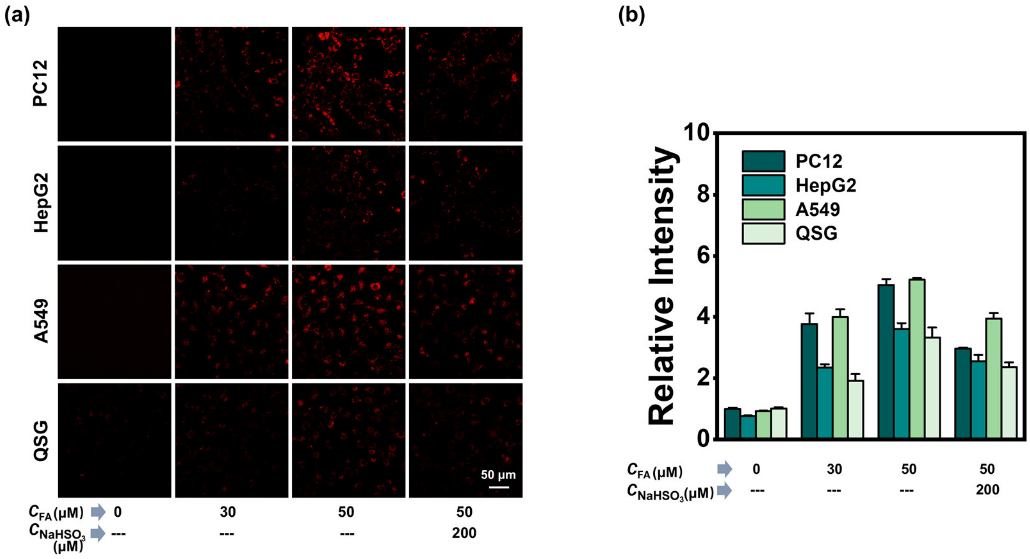

Figure S101. (a) Fluorescence image of different cells treated with **FB2** (5 μM, 2 h) or sodium bisulfite (200 μM, 2 h), and FA (0-50 μM, 0.5 h) at 37°C. Scale bar: 50 μm. (b) Mean relative fluorescence intensities of different cells. The error bar represents the standard deviation of relative fluorescence intensity measurements in independent cell (mean±S.D.), n=3.

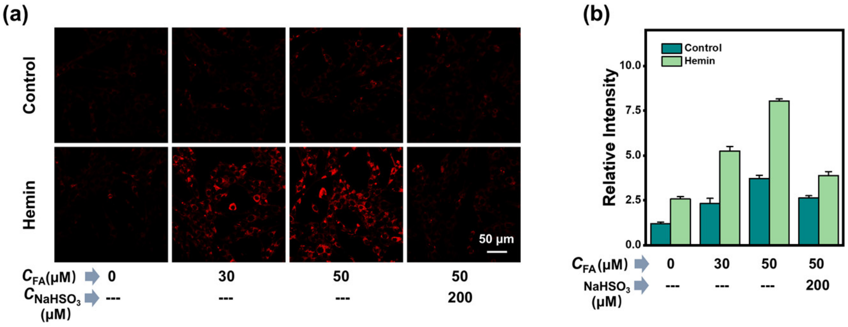

Figure S102. (a) Fluorescence image of PC12 cells treated with hemin (0 or 5 μM, 4.5 h), **FB1** (5 μM, 2 h) or sodium bisulfite (200 μM, 2 h), and FA (0-50 μM, 0.5 h) at 37°C. Scale bar: 50 μm. (b) Mean relative fluorescence intensities of PC12 cells. The error bar represents the standard deviation of relative fluorescence intensity measurements in independent cell (mean±S.D.), n=3.

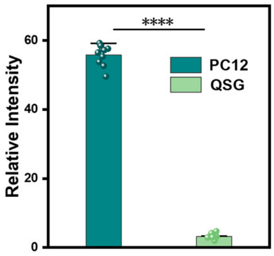

Figure S103. Mean relative fluorescence intensities of co-cultured PC12/QSG cells. The cells were pretreated with hemin (5 μM, 4.5 h), **FB2** (2 μM, 2 h), and FA (50 μM, 0.5 h) at 37°C. The error bar represents the standard deviation of relative fluorescence intensity measurements in independent cell (mean±S.D.), n=10, **** p<0.0001.

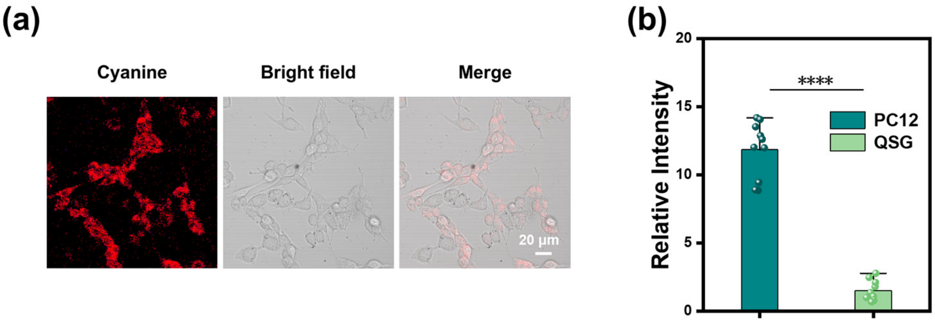

Figure S104. (a) CLSM images of PC12 cells co-cultured with QSG cells. The cells were pretreated with hemin (5 μM, 4.5 h), **FB1** (2 μM, 2 h), and FA (50 μM, 0.5 h) at 37°C. Scale bar: 20 μm. (b) Mean relative fluorescence intensities of co-cultured PC12/QSG cells. The error bar represents the standard deviation of relative fluorescence intensity measurements in independent cell (mean±S.D.), n=10, **** p<0.0001.

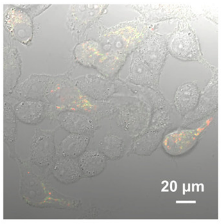

Figure S105. CLSM images of co-cultured HepG2/QSG cells stained with JC-1 in bright field.

4. X-Ray Crystal Structure Analysis

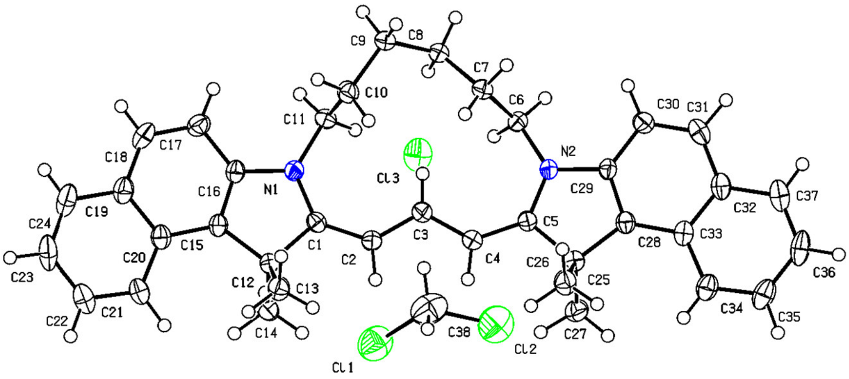

Figure S106. X-ray crystal structure of **CY-1**.

Table S3. Crystalographic data and structure refinement for **CY-1.**

Identification code	**CY-1**
Empirical formula	$C_{38}H_{41}Cl_3N_2$
Formula weight	632.08
Temperature/K	193.00
Crystal system	monoclinic
Space group	$P2_1/n$
a/Å	11.3572(10)
b/Å	13.1763(10)
c/Å	22.3783(15)
α/°	90
β/°	103.751(3)
γ/°	90
Volume/Å^3	3252.8(4)
Z	4
ρ_{calc}g/cm^3	1.291
μ/mm^{-1}	0.312
F(000)	1336.0

Crystal size/mm^3	0.16 × 0.12 × 0.1
Crystal colour	purple
Radiation	MoKα (λ = 0.71073)
2Θ range for data collection/°	3.614 to 54.988
Index ranges	-11 ≤ h ≤ 14, -17 ≤ k ≤ 17, -29 ≤ l ≤ 28
Reflections collected	30457
Independent reflections	7454 [R_{int} = 0.0817, R_{sigma} = 0.0747]
Data/restraints/parameters	7454/0/552
Goodness-of-fit on F^2	1.038
Final R indexes [I>=2σ (I)]	R_1 = 0.0565, wR_2 = 0.1106
Final R indexes [all data]	R_1 = 0.1111, wR_2 = 0.1346
Largest diff. peak/hole / e Å^{-3}	0.28/-0.41

Table S4. Fractional Atomic Coordinates (×10^4) and Equivalent Isotropic Displacement Parameters (Å^2×10^3) for **CY-1**. U_{eq} is defined as 1/3 of the trace of the orthogonalised U_{IJ} tensor.

**Atom**	***x***	***y***	***z***	**U(eq)**
Cl2	614.1(9)	5175.5(7)	1143.6(5)	69.9(3)
Cl1	-423.6(9)	5525.1(7)	2196.9(4)	69.7(3)
N1	3897.2(18)	6596.3(14)	4032.8(8)	23.6(4)
N2	5387.8(19)	5432.1(14)	1697.3(8)	24.0(4)
C16	3717(2)	6734.8(18)	4632.7(10)	24.7(5)
C5	4691(2)	4717.0(18)	1872.4(10)	23.5(5)
C15	3041(2)	5950.9(18)	4775.2(10)	23.9(5)
C12	2780(2)	5205.1(17)	4246.8(10)	23.1(5)
C29	5843(2)	5081.1(18)	1195.8(10)	22.8(5)
C33	5666(2)	3650.3(18)	505.3(10)	25.5(5)
C1	3415(2)	5705.8(17)	3793.7(10)	22.6(5)
C6	5533(2)	6473.1(18)	1929.5(11)	25.7(5)
C28	5385(2)	4132.5(18)	1021.4(10)	24.2(5)
C3	4055(2)	5434.4(19)	2798.1(10)	25.1(5)
C11	4601(2)	7303.9(19)	3747.2(11)	26.5(5)
C2	3457(2)	5208.8(19)	3253.3(10)	25.9(5)
C20	2761(2)	5903.0(18)	5360.1(10)	27.1(5)
C25	4636(2)	3786.3(18)	1456.1(10)	24.1(5)
C4	4087(2)	4724.9(19)	2342.5(10)	26.5(5)
C32	6471(2)	4168.0(19)	206.2(10)	28.6(6)
C13	3393(3)	4178(2)	4441.4(12)	30.3(6)
C30	6637(2)	5600(2)	911.5(11)	28.6(6)
C26	5210(3)	2876(2)	1847.9(13)	32.6(6)

C7	6459(2)	6603(2)	2540.0(11)	28.1(6)
C21	2062(2)	5125(2)	5546.8(11)	31.4(6)
C14	1428(2)	5084(2)	3943.7(12)	31.2(6)
C19	3243(3)	6686.1(19)	5788.5(11)	32.1(6)
C10	5919(2)	6993(2)	3869.5(11)	28.8(6)
C34	5184(3)	2701(2)	261.2(11)	32.2(6)
C17	4174(3)	7523.3(19)	5045.3(12)	31.2(6)
C8	6266(3)	7624(2)	2834.5(11)	29.9(6)
C18	3930(3)	7476(2)	5613.0(12)	35.3(7)
C9	6674(3)	7656(2)	3538.6(11)	31.4(6)
C27	3309(3)	3551(2)	1143.5(13)	33.4(6)
C31	6949(2)	5128(2)	427.9(11)	32.8(6)
C37	6767(3)	3708(2)	-314.2(12)	37.4(7)
C22	1872(3)	5116(2)	6127.0(12)	38.6(7)
C35	5466(3)	2304(2)	-252.4(12)	38.5(7)
C36	6272(3)	2808(2)	-538.4(12)	40.3(7)
C24	3027(3)	6636(2)	6389.2(12)	45.1(8)
C23	2367(3)	5874(2)	6550.9(13)	47.1(8)
C38	936(3)	5342(3)	1955.1(18)	62.8(10)
Cl3	2746.3(7)	7719.9(6)	2179.7(3)	47.3(2)

5. References

1 O. V. Dolomanov, L. J. Bourhis, R. J. Gildea, J. A. K. Howard and H. Puschmann, *J. Appl. Crystallogr.,* 2009, **42**, 339-341.

2 L. J. Bourhis, O. V. Dolomanov, R. J. Gildea, J. A. K. Howard and H. Puschmann, *Acta Crystallogr., Sect. A: Found. Adv.,* 2015, **71**, 59-75.

3 G. M. Sheldrick, *Acta Crystallogr., Sect. C: Struct. Chem.,* 2015, **71**, 3-8.

4 a) X. Ren, C. Wang, X. Wu, M. Rong, R. Huang, Q. Liang, T. Shen, H. Sun, R. Zhang, Z. Zhang, X. Liu, X. Song and J. W. Foley, *J. Am. Chem. Soc.*, 2024, **146**, 6566-6579; b) X.-F. Zhang, Y. Zhang and L. Liu, *J. Lumin.,* 2014, **145**, 448-453.

5 Z. Zhang, C. Gao, Z. Lu, X. Xie, J. You and Z. Li, *Biosens. Bioelectron.,* 2023, **237**, 115485-115495.
